# Design, synthesis, and antiproliferative activity of new 2-amino-pyrano[3,2-*c*]quinoline-3-carbonitriles as potential EGFR, BRAF^V600E^, and HER-2 inhibitors

**DOI:** 10.1039/d5ra04276c

**Published:** 2025-10-13

**Authors:** Lamya H. Al-Wahaibi, Aliaa M. Mohassab, Safwat M. Rabea, Bahaa G. M. Youssif, Stefan Bräse, Essmat M. El-Sheref

**Affiliations:** a Department of Chemistry, College of Sciences, Princess Nourah bint Abdulrahman University Riyadh 11671 Saudi Arabia; b Department of Medicinal Chemistry, Faculty of Pharmacy, Minia University Minia 61519 Egypt; c Department of Pharmaceutical Organic Chemistry, Faculty of Pharmacy, Assiut University Assiut 71526 Egypt bgyoussif2@gmail.com +20-01098294419; d Institute of Biological and Chemical Systems, IBCS-FMS, Karlsruhe Institute of Technology 76131 Karlsruhe Germany braese@kit.edu; e Chemistry Department, Faculty of Science, Minia University El Minia 61519 Egypt +20-01064890489

## Abstract

A novel series of pyrano-quinoline compounds 5a–l was designed, synthesized, and investigated for antiproliferative efficacy as multi-EGFR/HER-2/BRAF^V600E^ inhibitors. This work addresses the reaction between 4-hydroxy-2-oxo-1,2-dihydroquinolines and 2-benzylidenemalononitriles, which produces a new series of 2-amino-5-oxo-4-phenyl-5,6-dihydro-4*H*-pyrano[3,2-*c*]quinoline-3-carbonitrile derivatives 5a–l, giving good yields. The suggested mechanism was considered. The structures of 5a–l were elucidated using NMR spectroscopy, mass spectrometry, and elemental analysis. The cell viability assay of 5a–l against a normal cell line showed that none of the studied compounds exhibited cytotoxicity, and all hybrids retained above 90% cell viability at a dose of 50 μM. The antiproliferative activity of 5a–l was assessed against a panel of four cancer cell lines using the MTT assay. Compounds 5e and 5h had the most antiproliferative activity, with GI_50_ values of 26 and 28 nM, respectively, making them more efficient than erlotinib (GI_50_ = 33 nM). Inhibitory assays on EGFR, HER-2, and BRAF^V600E^ indicated that compounds 5e and 5h were the most efficacious derivatives, with IC_50_ values of 71 nM (EGFR), 62 nM (BRAF^V600E^), and 21 nM (HER-2) for compound 5e, whereas compound 5h displayed IC_50_ values of 75 nM (EGFR), 67 nM (BRAF^V600E^), and 23 nM (HER-2). Molecular docking studies were conducted on a series of quinoline-based compounds to evaluate their binding affinity with EGFR and HER-2 kinases. Compound 5e showed promising interactions, forming stable complexes with key residues like Met769 (EGFR) and Asp863 (HER-2). The docking simulations revealed critical hydrogen bonding, π–π stacking, and hydrophobic interactions, supporting its potential as a kinase inhibitor for cancer treatment.

## Abstract 2

A novel series of quinolines 5a–l was investigated for antiproliferative efficacy as multi-EGFR/HER-2/BRAF^V600E^ inhibitors. This work addresses the synthesis of a new series of pyrano[3,2-*c*]quinoline-3-carbonitriles 5a–l.

## Graphical abstract

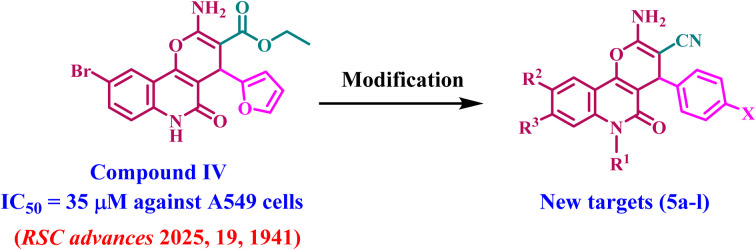

## 1. Introduction

Cancer is a complex disease whose increasing prevalence is significantly impacting human health.^1^ Chemotherapy, a pivotal therapeutic modality for cancer, has consistently garnered significant attention from researchers and clinicians.^2^ The advancement of anticancer drugs exhibiting enhanced therapeutic efficacy and reduced clinical adverse effects has garnered increasing interest from medicinal chemists.^3,4^ Oncology has experienced significant breakthroughs since establishing targeted therapy utilizing small-molecule inhibitors.^5,6^ The latter refers to the treatment that directly targets the primary causes of cancer formation, which may include dysregulated enzymes and proteins.

The protein kinase family, recognized as a prominent class of carcinogenic drug targets, is responsible for the phosphorylation reaction essential in various physiological functions.^7,8^ The overexpression, disruption, mutations, and translocation of these proteins led to various disorders, particularly cancer.^9,10^ Recently, 80 small compounds that inhibit various kinases have received FDA approval, establishing kinase family proteins among the most targeted proteins in cancer research.^11,12^

Quinoline has emerged as a significant scaffold in drug development in recent decades, especially within cancer research. Quinoline, a nitrogen-containing heterocyclic molecule, has a variety of biological activities.^13–17^ Compounds containing quinoline exhibit markedly increased basicity owing to the presence of nitrogen atoms. Clinical trials are presently investigating numerous anticancer agents that feature the quinoline moiety.^18–20^ Quinoline derivatives effectively combat cancer by multiple mechanisms, including inhibiting protein kinase, epidermal growth factor receptor (EGFR), and mitogen-activated protein kinases.^20–22^ Anticancer agents developed from quinoline encompass bosutinib, lenvatinib, and cabozantinib, all of which function as protein kinase inhibitors.^23^ Quinoline derivatives have demonstrated potential in several cancer cell lines, including those originating from the breast, colon, lung, colorectal, and renal tissues.^24^

**Fig. 1:**
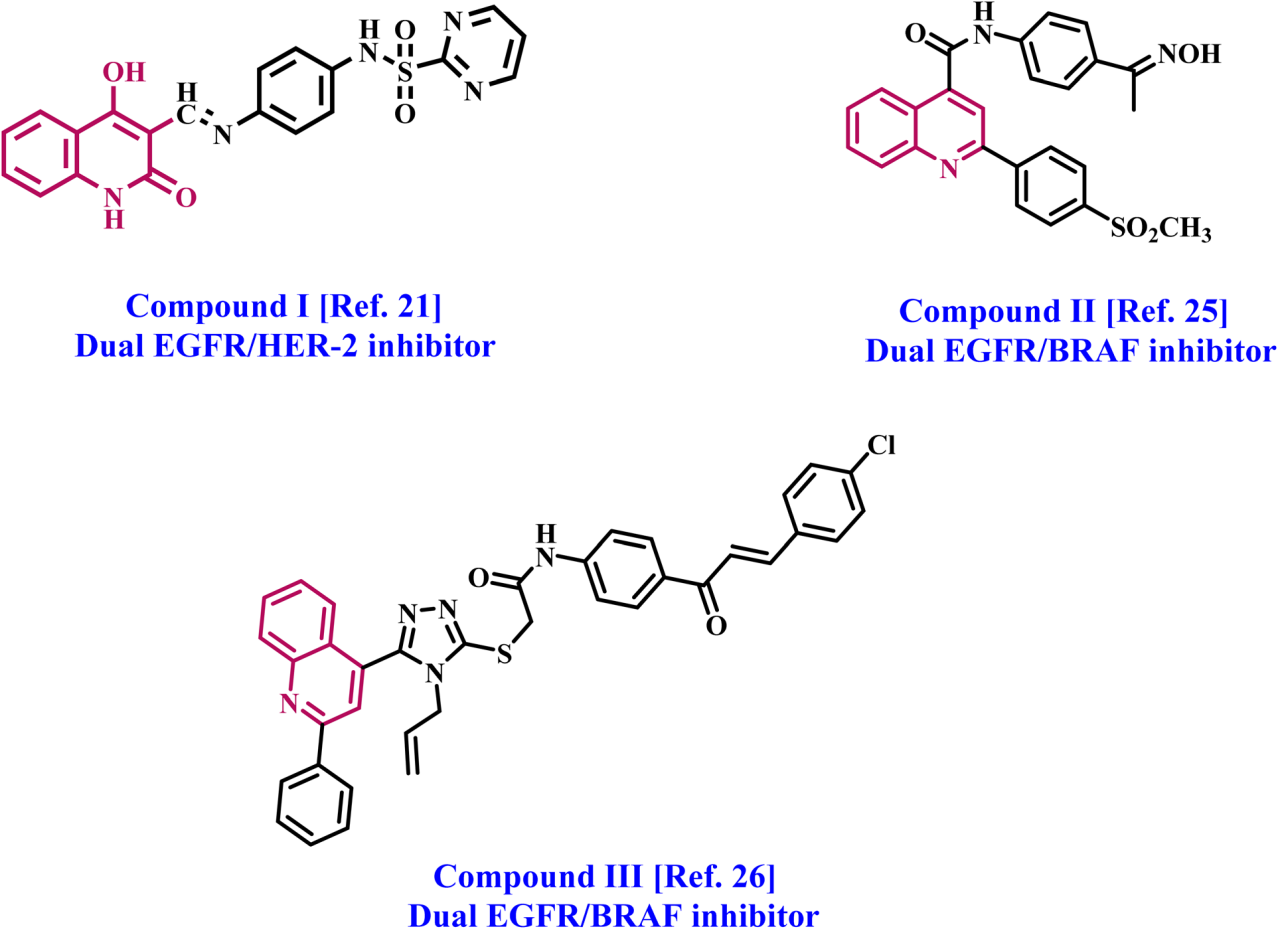
Structures of compounds I–III, quinoline-based moieties, as inhibitors of protein kinase.

We recently^21^ reported the design and synthesis of a new class of EGFR/HER-2 dual-target inhibitors derived from quinoline compounds. The new compounds were evaluated for antiproliferative efficacy against four cancer cell lines, demonstrating considerable antiproliferative activity, particularly in breast (MCF-7) and lung (A-549) cancer cell lines, which exhibited the highest sensitivity. Compound I (Fig. 1) had the most pronounced antiproliferative effect. Compound I demonstrated the highest efficacy as a dual-target inhibitor of EGFR and HER-2, with inhibitory (IC_50_) values of 71 and 31 nM, respectively. It surpassed the reference erlotinib (IC_50_ = 80 nM) as an EGFR inhibitor but was comparable to the clinically utilized drug lapatinib (IC_50_ = 26 nM) as a HER-2 inhibitor. Furthermore, results show that compound I promotes apoptosis by activating caspase-3, 8, and Bax while downregulating the expression of the anti-apoptotic protein Bcl2.

In a different study completed in our laboratory,^25^ we continued developing and synthesizing innovative quinoline-derived compounds as prospective antiproliferative medicines. We assessed the antiproliferative effect of the newly synthesized compounds against four human cancer cell lines. Compound II (Fig. 1) demonstrated superior efficacy to the standard medication doxorubicin against the four cancer cell lines (GI_50_ = 1.40 μM *vs.* 1.20 μM for II). Compound II exhibited the highest efficacy in inhibiting EGFR and BRAF^V600E^, with IC_50_ values of 105 ± 10 and 140 ± 12 nM, respectively. The values were comparable to those of the conventional medication erlotinib, which exhibited IC_50_ values of 80 ± 10 and 60 ± 10 nM, respectively.

In 2021,^26^ we detailed synthesizing a new series of quinoline-based compounds employed as antiproliferative agents targeting EGFR and BRAF^V600E^. Compound III (Fig. 1) exhibited enhanced antiproliferative efficacy relative to doxorubicin (GI_50_ = 1.15 μM). It demonstrated a GI_50_ value of 3.30 μM against four human cancer cell lines. Compound III demonstrated inhibitory effectiveness against EGFR and BRAF^V600E^, with IC_50_ values of 1.30 ± 0.12 μM and 3.80 ± 0.15 μM, respectively. In contrast, the reference erlotinib exhibited IC_50_ values of 0.08 ± 0.005 μM for EGFR and 0.06 ± 0.01 μM for BRAF^V600E^.

### 1.1. Rational design

**Fig. 2:**
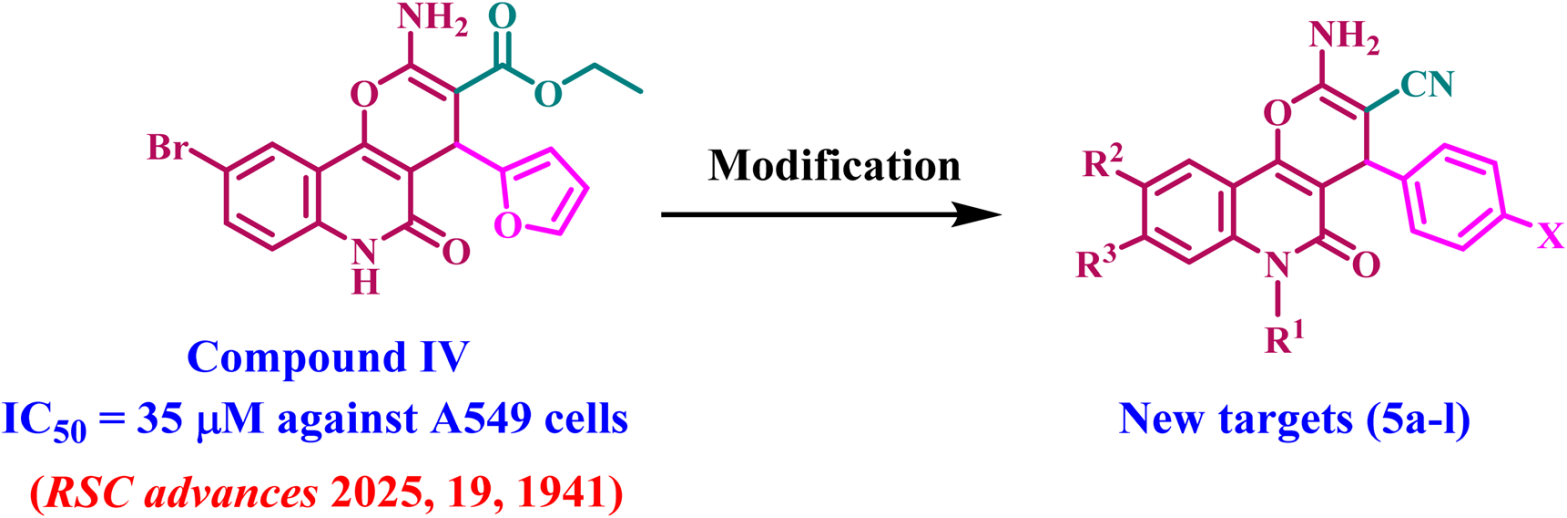
Structures of previously described compound IV and newly developed compounds 5a–l.

Aly *et al.*,^27^ reported the synthesis and anticancer efficacy of a novel series of 2-amino-4-(furan-2-yl)-4*H*-pyrano[3,2-*c*]quinoline-3-carboxylates. Compound IV (Fig. 2) has been identified as the most efficient derivative, with an IC_50_ value of 35 μM against A-549 lung epithelial cancer cells. The *in vitro* inhibitory profile of topoisomerase II for the most efficient derivative, compound IV, was examined. Compound IV had moderate to weak inhibitory action with an IC_50_ value of 45.19 μM.

The present study continues our endeavor to develop dual or multi-targeted protein kinase inhibitors,^21,22,28,29^ presenting the design, synthesis, and antiproliferative effects of a novel series of pyrano-quinolines 5a–l, Fig. 2. The newly synthesized compounds were developed through structural changes of compound IV, as illustrated in Fig. 2. The ester group at position 3 of 2-amino-pyrano[3,2-*c*]quinoline was substituted with a nitrile group. The carbonitrile group has notable and varied biological actions, with anticancer properties being the most prominent. It is defined by its rigidity, stability under *in vivo* conditions, capacity for hydrogen bonding with diverse protein targets, and moderate dipole features.^30^ The second modification involves substituting the furan moiety with a benzene ring. These modifications were incorporated, hopefully, to enhance the pharmacokinetics and/or pharmacodynamics of novel 5a–l compounds.

## 2. Experimental

### 2.1. Chemistry

General details: see Appendix A (SI).

4-Hydroxy-2-oxo-1,2-dihydroquinoline derivatives 1a–e,^31^ and 2-benzylidenemalononitriles 4a–c (ref. 32) were prepared according to reported procedures.

#### 2.1.1. Synthesis of 2-amino-5-oxo-5,6-dihydro-4*H*-pyrano[3,2-*c*]quinoline-3-carbonitriles 5a–l

##### 2.1.1.1. Methods I and II

A mixture of 1a–e (1 mmol) and 4a–c (1 mmol) in 50 mL of absolute ethanol and a few drops of Et_3_N (method I) or anhydrous K_2_CO_3_ (method II) (1.5 mmol, 0.192 g) was refluxed for 12 h. The reaction was observed *via* TLC analysis. Upon completion of the reaction, allow it to cool to room temperature. The resultant compounds 5a–l were further filtered and washed several times with ethanol (method I) or a mixture of ethanol and water (method II to eliminate K_2_CO_3_) and then dried. The obtained products were recrystallized from the specified solvents to yield pure 5a–l.

##### 2.1.1.2. Method III

A mixture of 1a–e (1 mmol) and 4a–c (1 mmol) in 40 mL of DMF, together with anhydrous K_2_CO_3_ (1.5 mmol, 0.192 g), was stirred at ambient temperature for 24 h. The reaction was observed *via* TLC analysis. Upon completion of the reaction, 100 g of crushed ice was introduced with stirring, and the products were filtered, washed repeatedly with water, and dried. The resultant products were recrystallized from the specified solvents to yield pure 5a–l.

###### 2.1.1.2.1. 2-Amino-5-oxo-4-phenyl-5,6-dihydro-4*H*-pyrano[3,2-*c*]quinoline-3-carbonitrile (5a)

Colorless crystals (EtOH) (80%); m.p.: 304–306 °C; ^1^H NMR (DMSO-*d*_6_): *δ*_H_ = 11.77 (s, 1H, NH), 7.93 (d, *J* = 8.0 Hz; 1H), 7.58 (t, *J* = 7.7 Hz, 1H), 7.42–7.06 (m, 9H, Ph-H, Q-H and NH_2_), 4.51 ppm (s, 1H, H-4); ^13^C NMR (DMSO-*d*_6_): *δ*_C_ = 160.94 (CO), 159.45 (C-2), 151.69 (C-10b), 144.84 (Ph-C), 138.26 (C-6a), 131.69, 128.86, 127.85, 127.20, 122.46, 122.25 (Ar-CH), 120.31 (CN), 115.83 (C-10a), 112.48 (C-4a), 110.07 (C-7), 58.28 (C-3), 37.19 ppm (C-4): anal. calcd for C_19_H_13_N_3_O_2_: C, 72.37; H, 4.16; N, 13.33; found: C, 72.47; H, 4.21; N, 13.49.

###### 2.1.1.2.2. 2-Amino-9-methyl-5-oxo-4-phenyl-5,6-dihydro-4*H*-pyrano[3,2-*c*]quinoline-3-carbonitrile (5b)

Colorless crystals (EtOH) (90%); m.p.: 309–311 °C; ^1^H NMR (DMSO-*d*_6_): *δ*_H_ = 11.68 (s, 1H, NH), 7.73 (s, 1H, H-10), 7.42 (d, *J* = 6 Hz, 1H, H-8), 7.25 (m, 8H, H-7, NH_2_, Ph-CH), 4.49 (s, 1H, H-4), 2.40 ppm (s, 3H, Me); ^13^C NMR (DMSO-*d*_6_): *δ*_C_ = 160.80 (CO), 159.50 (C-2), 151.56 (C-10b), 144.91 (Ph-C), 136.30 (C-6a), 132.89 (C-9), 131.60 (C-8), 128.85, 127.81, 127.18, 121.70 (Ph-CH), 120.30 (CN), 115.78 (C-10a), 112.38 (C-7), 110.02 (C-4a), 58.30 (C-3), 37.20 (C-4), 21.13 ppm (Me): anal. calcd for C_20_H_15_N_3_O_2_: C, 72.94; H, 4.59; N, 12.76; found: C, 72.88; H, 4.77; N, 12.71.

###### 2.1.1.2.3. 2-Amino-9-methoxy-5-oxo-4-phenyl-5,6-dihydro-4*H*-pyrano[3,2-*c*]quinoline-3-carbonitrile (5c)

Colorless crystals (EtOH) (92%); m.p.: 312–314 °C; ^1^H NMR (DMSO-*d*_6_): *δ*_H_ = 11.66 (s, 1H, NH), 7.44 (d, *J* = 2.6 Hz, 1H, H-8), 7.32–7.18 (m, 9H, q-H-7,10, Ph-H, NH_2_), 4.51 (s, 1H, H-4), 3.85 ppm (s, 3H, OCH_3_); ^13^C NMR (DMSO-*d*_6_): *δ*_C_ = 159.99 (CO), 159.06 (C-2), 154.42 (C-9), 150.88 (C-10b), 144.42 (Ph-C), 132.33 (C-6a), 128.42, 127.39, 126.74, 120.46 (Ph-CH, q-C-7,10), 120.00 (CN), 116.86 (C-10a), 112.50 (C-8), 109.92 (C-4a), 57.68 (C-3), 55.57 (OMe), 36.76 ppm (C-4): anal. calcd for C_20_H_15_N_3_O_3_: C, 69.56; H, 4.38; N, 12.17; found: C, 69.74; H, 4.29; N, 12.09.

###### 2.1.1.2.4. 2-Amino-8-methyl-5-oxo-4-phenyl-5,6-dihydro-4*H*-pyrano[3,2-*c*]quinoline-3-carbonitrile (5d)

Colorless crystals (EtOH) (90%); m.p.: 300–302 °C; ^1^H NMR (DMSO-*d*_6_): *δ*_H_ = 11.74 (s, 1H, NH), 7.74–7.19 (m, 10H, H-7,9,10, Ph-CH, NH_2_), 4.53 (s, 1H, H-4), 2.78 ppm (s, 3H, Me); ^13^C NMR (DMSO-*d*_6_): *δ*_C_ = 160.94 (CO), 159.45 (C-2), 151.69 (C-10b), 144.84 (Ph-C), 138.26 (C-8), 131.69 (C-6a), 128.86, 127.85, 127.20, 122.66, 122.46 (Ar-CH), 120.31 (CN), 115.93 (C-7), 112.88 (C-10a), 110.07 (C-4a), 58.28 (C-3), 37.19 (C-4), 21.23 ppm (Me): anal. calcd for C_20_H_15_N_3_O_2_: C, 72.94; H, 4.59; N, 12.76; found: C, 73.01; H, 4.66; N, 12.61.

###### 2.1.1.2.5. 2-Amino-6-methyl-5-oxo-4-phenyl-5,6-dihydro-4*H*-pyrano[3,2-*c*]quinoline-3-carbonitrile (5e)

Colorless crystals (EtOH) (78%); m.p.: 290–292 °C; ^1^H NMR (DMSO-*d*_6_): *δ*_H_ = 8.03 (d, *J* = 8.0 Hz, 1H), 7.70 (t, *J* = 7.9 Hz, 1H), 7.55 (d, *J* = 8.6 Hz, 1H), 7.39 (t, *J* = 7.6 Hz, 1H), 7.27 (d, *J* = 5.5 Hz, 4H), 7.20 (dd, *J* = 11.7, 7.1 Hz, 1H, NH_2_), 4.52 (s, 1H, H-4), 3.53 ppm (s, 3H, N–Me); ^13^C NMR (DMSO-*d*_6_): *δ*_C_ = 160.84 (CO), 159.45 (C-2), 151.64 (C-10b), 144.84 (Ph-C), 138.26 (C-6a), 131.69, 128.80, 127.85, 127.24, 122.46, 122.25 (Ar-CH), 120.30 (CN), 115.83 (C-10a), 112.48 (C-4a), 110.07 (C-7), 58.20 (C-3), 37.30 (C-4), 29.69 ppm (N–Me): anal. calcd for C_20_H_15_N_3_O_2_: C, 72.94; H, 4.59; N, 12.76; found: C, 72.87; H, 4.76; N, 12.88.

###### 2.1.1.2.6. 2-Amino-4-(4-methoxylphenyl)-5-oxo-5,6-dihydro-4*H*-pyrano[3,2-*c*]quinoline-3-carbonitrile (5f)

Yellow crystals (EtOH) (86%); m.p.: 301–303 °C; ^1^H NMR (DMSO-*d*_6_): *δ*_H_ = 11.76 (s, 1H, q-NH), 7.92 (d, *J* = 8.0 Hz, 1H, q-H-7), 7.57 (t, *J* = 7.7 Hz, 1H, q-H-8), 7.37–7.18 (m, 4H, q-H-9,10, NH_2_), 7.14 (d, *J* = 8.6 Hz, 2H, Ph-H-*o*), 6.85 (d, *J* = 8.6 Hz, 2H, Ph-H-*m*), 4.47 (s, 1H, H-4), 3.71 ppm (s, 3H, OCH_3_); ^13^C NMR (DMSO-*d*_6_): *δ*_C_ = 160.56 (CO), 158.95 (C-2), 158.11 (Ph-C-OMe), 150.96 (C-10b), 137.75 (Ph-C), 136.52 (C-6a), 131.17, 128.55 (Ph-CH), 121.99, 121.79 (q-CH), 120.02 (CN), 115.38 (C-10a), 113.77 (C-7), 109.93 (C-4a), 58.05 (C-3), 55.05 (OMe), 35.96 ppm (C-4): anal. calcd for C_20_H_15_N_3_O_3_: C, 69.56; H, 4.38; N, 12.17; found: C, 69.43; H, 4.44; N, 12.29.

###### 2.1.1.2.7. 2-Amino-4-(4-methoxylphenyl)-9-methyl-5-oxo-5,6-dihydro-4*H*-pyrano[3,2-*c*]quinoline-3-carbonitrile (5g)

Yellow crystals (EtOH) (89%); m.p.: 317–319 °C; ^1^H NMR (DMSO-*d*_6_): *δ*_H_ = 11.67 (s, 1H, q-NH), 7.73 (s, 1H, q-H-10), 7.40 (d, *J* = 8.3 Hz, 1H, q-H-8), 7.31–7.16 (m, 3H, q-H-7, NH_2_), 7.12 (d, *J* = 8.5 Hz, 2H, Ph-H-*o*), 6.85 (d, *J* = 8.5 Hz, 2H, Ph-H-*m*), 4.44 (s, 1H, H-4), 3.70 (s, 3H, OCH_3_), 2.39 ppm (s, 3H, CH_3_); ^13^C NMR (DMSO-*d*_6_): *δ*_C_ = 160.40 (CO), 158.99 (C-2), 158.08 (Ph-C-OMe), 150.80 (C-10b), 136.59 (Ph-C), 135.79 (C-6a), 132.35 (C-9), 131.09 (Ph-CH), 128.50, 121.22 (q-CH), 120.01 (CN), 115.30 (C-10a), 113.75 (C-7), 111.97 (Ph-CH), 109.86 (C-4a), 58.02 (C-3), 55.03 (OMe), 35.96 (C-4), 2070 ppm (Me): anal. calcd for C_21_H_17_N_3_O_3_: C, 70.18; H, 4.77; N, 11.69; found: C, 69.99; H, 4.70; N, 11.86.

###### 2.1.1.2.8. 2-Amino-9-methoxyl-4-(4-methoxylphenyl)-5-oxo-5,6-dihydro-4*H*-pyrano[3,2-*c*]quinoline-3-carbonitrile (5h)

Colorless crystals (EtOH) (90%); m.p.: 320–322 °C; ^1^H NMR (DMSO-*d*_6_): *δ*_H_ = 11.64 (s, 1H, q-NH), 7.42 (d, *J* = 2.5 Hz, 1H, q-H-7), 7.29 (s, 1H, q-H-10), 7.27 (bs, 2H, NH_2_), 7.25 (d, *J* = 8.7, 1H, q-H-8), 7.12 (d, *J* = 8.6 Hz, 2H, Ph-H-*o*), 6.85 (d, *J* = 8.6 Hz, 2H, Ph-H-*m*), 4.44 (s, 1H, H-4), 3.84 (s, 3H, OCH_3_), 3.71 ppm (s, 3H, OCH_3_); ^13^C NMR (DMSO-*d*_6_): *δ*_C_ = 160.01 (CO), 159.00 (C-2), 158.07 (Ph-C-OMe), 154.40 (C-9), 150.57 (C-10b), 136.50 (Ph-C), 132.25 (C-6a), 128.48 (Ph-CH), 120.36 (q-C-7), 120.00 (CN), 116.82 (q-C-8), 113.74 (Ph-CH), 112.54 (q-C-10), 110.23 (C-4a), 58.00 (C-3), 55.55 (OMe), 55.04 (OMe), 35.96 ppm (C-4): anal. calcd for C_21_H_17_N_3_O_4_: C, 67.19; H, 4.56; N, 11.19; found: C, 67.28; H, 4.72; N, 11.13.

###### 2.1.1.2.9. 2-Amino-6-methyl-4-(4-methoxylphenyl)-5-oxo-5,6-dihydro-4*H*-pyrano[3,2-*c*]quinoline-3-carbonitrile (5i)

Colorless crystals (EtOH) (78%); m.p.: 296–298 °C; ^1^H NMR (DMSO-*d*_6_): *δ*_H_ = 8.02 (d, *J* = 7.2 Hz, 1H, q-H-10), 7.70 (t, *J* = 8.2 Hz, 1H, q-H-8), 7.55 (d, *J* = 8.5 Hz, 1H, q-H-7), 7.39 (t, *J* = 7.5, 1H, q-H-8), 7.24 (s, 2H, NH_2_), 7.13 (d, *J* = 8.6 Hz, 2H, Ph-H-*o*), 6.84 (d, *J* = 8.6 Hz, 2H, Ph-H-*m*), 4.47 (s, 1H, H-4), 3.71 (s, 3H, OCH_3_), 3.54 ppm (s, 3H, N–CH_3_); ^13^C NMR (DMSO-*d*_6_): *δ*_C_ = 159.77 (CO), 158.8 (C-2), 158.08 (Ph-C-OMe), 149.80 (C-10b), 138.50 (q-C-6a), 136.45 (Ph-C), 131.46 (Ph-CH), 128.65, 121.10 (q-CH), 119.98 (CN), 114.82 (Ph-CH), 113.67 (C-7), 109.25 (C-4a), 58.16 (C-3), 55.01 (OMe), 36.54 (C-4), 29.22 ppm (N–Me): anal. calcd for C_21_H_17_N_3_O_3_: C, 70.18; H, 4.77; N, 11.69; found: C, 70.31; H, 4.63; N, 11.55.

###### 2.1.1.2.10. 2-Amino-4-(4-chlorophenyl)-5-oxo-5,6-dihydro-4*H*-pyrano[3,2-*c*]quinoline-3-carbonitrile (5j)

Colorless crystals (EtOH) (70%); m.p.: 298–300 °C; ^1^H NMR (DMSO-*d*_6_): *δ*_H_ = 11.79 (s, 1H, NH), 7.92 (d, *J* = 7.9 Hz, 1H, q-H-10), 7.58 (t, *J* = 7.6 Hz, 1H, q-H-7), 7.32 (dd, *J* = 18.1, 5.9 Hz, 6H, Ph-H, NH_2_), 7.26 (t, *J* = 8.1, 2H, q-H-8,9), 4.53 ppm (s, 1H, H-4). Anal. calcd for C_19_H_12_ClN_3_O_2_: C, 65.24; H, 3.46; N, 12.01; found: C, 65.37; H, 3.39; N, 12.18.

###### 2.1.1.2.11. 2-Amino-9-methyl-4-(4-chlorophenyl)-5-oxo-5,6-dihydro-4*H*-pyrano[3,2-*c*]quinoline-3-carbonitrile (5k)

Colorless crystals (EtOH) (68%); m.p.: 300–302 °C; ^1^H NMR (DMSO-*d*_6_): *δ*_H_ = 11.71 (s, 1H, NH), 7.73 (s, 1H, q-H-10), 7.42 (dd, *J* = 8.5, 1.8 Hz, 1H, q-H-8), 7.35 (d, *J* = 8.5 Hz, 2H, ph-H-*o*), 7.25 (dd, *J* = 16.1, 9.6 Hz, 5H, q-H-7, Ph-H-m, NH_2_), 4.51 (s, 1H, H-4), 2.40 ppm (s, 3H, Me); ^13^C NMR (DMSO-*d*_6_): *δ*_C_ = 160.30 (CO), 158.97 (C-2), 151.12 (C-10b), 143.48 (Ph-C), 135.92 (C-6a), 132.55 (C-9), 131.27 (Ph-C-Cl), 131.16, 129.36 (Ph-CH), 128.35 (q-C-8), 121.28 (q-C-7), 119.75 (CN), 115.36 (C-10a), 109.00 (C-4a), 57.27 (C-3), 36.28 (C-4), 20.70 ppm (Me): anal. calcd for C_20_H_14_ClN_3_O_2_: C, 66.03; H, 3.88; N, 11.55; found: C, 65.91; H, 4.02; N, 11.49.

###### 2.1.1.2.12. 2-Amino-9-methoxyl-4-(4-chlorophenyl)-5-oxo-5,6-dihydro-4*H*-pyrano[3,2-*c*]quinoline-3-carbonitrile (5l)

Colorless crystals (EtOH) (65%); m.p.: 310–312 °C; ^1^H NMR (DMSO-*d*_6_): *δ*_H_ = 11.68 (s, 1H, NH), 7.33 (d, *J* = 2.5 Hz, 1H, q-H-7), 7.37–7.21 (m, 8H, q-H-8,9,10, ph-H, NH_2_), 4.52 (s, 1H, H-4), 3.85 ppm (s, 3H, OMe); ^13^C NMR (DMSO-*d*_6_): *δ*_C_ = 159.97 (CO), 159.02 (C-2), 154.46 (C-9), 150.93 (C-10b), 143.43 (Ph-C), 132.40 (Ph-C-Cl), 131.30 (q-C-6a), 129.38, 128.34 (Ph-CH), 120.56 (q-C-7), 119.77 (CN), 116.90 (C-10a), 112.45 (q-C-8), 109.37 (C-4a), 57.22 (C-3), 55.58 (OMe), 36.31 ppm (C-4): anal. calcd for C_20_H_14_ClN_3_O_3_: C, 63.25; H, 3.72; N, 11.06; found: C, 63.33; H, 3.89; N, 10.92.

### 2.2. Biology

#### 2.2.1. Cell viability assay

The human mammary gland epithelial (MCF-10A) normal cell line was used to examine the viability effects of new derivatives 5a–l using the MTT test.^33,34^ Refer to Appendix A for more details.

#### 2.2.2. Antiproliferative assay

The MTT assay was used to assess the antiproliferative activity of 5a–l against four human cancer cell lines, with erlotinib serving as a control.^35,36^ Appendix A has more information.

#### 2.2.3. EGFR inhibitory assay

The EGFR-TK assay^37^ assessed the inhibitory activity of the most potent antiproliferative derivatives, 5a, 5d, 5e, 5h, and 5i, against the EGFR. For more details, see Appendix A.

#### 2.2.4. BRAF^V600E^ inhibitory assay

Compounds 5a, 5d, 5e, 5h, and 5i were assessed for their ability to inhibit BRAF^V600E^, with erlotinib as the reference agent.^38^ The outcomes are presented as IC_50_ values. Appendix A outlines additional experimental details.

#### 2.2.5. HER-2 inhibitory assay

The kinase assay^39^ was used to assess the inhibitory activity of compounds 5a, 5d, 5e, 5h, and 5i against HER-2. The results are presented as IC_50_ values. Lapatinib served as the reference medication. Appendix A describes more experimental details.

## 3. Results and discussion

### 3.1. Chemistry

**Scheme 1:**
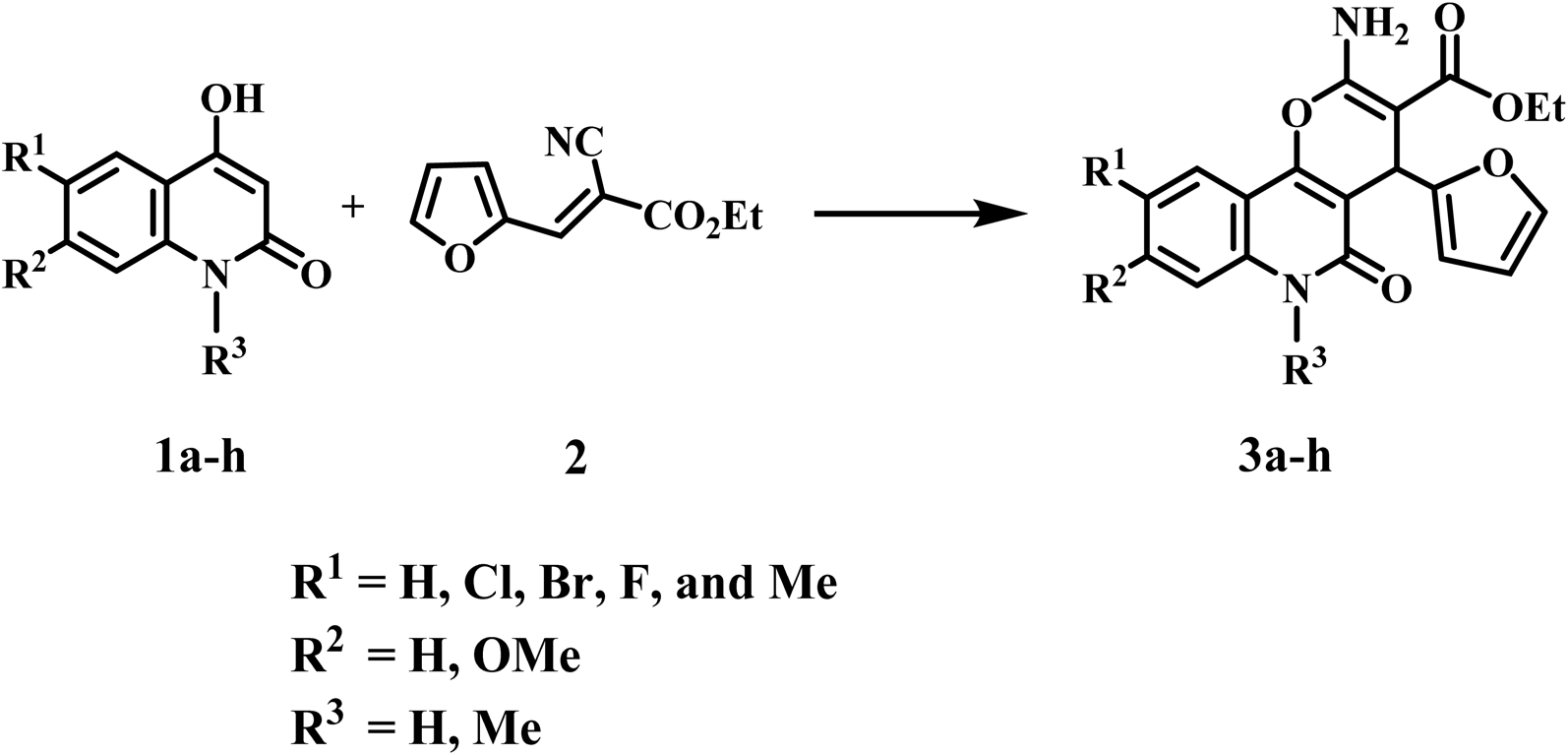
Synthesis of pyrano[3,2-*c*]quinoline-3-carboxylates 3a–h.

Aly *et al.*,^27^ previously reported the synthesis of pyrano[3,2-*c*]quinoline-3-carboxylate derivatives. The synthesis of compounds 3a–h was achieved through the reaction of 4-hydroxy-2-oxo-1,2-dihydroquinoline derivatives 1a–h with ethyl (*E*)-2-cyano-3-(furan-2-yl)acrylate (2), and their biological properties were investigated as dual-function anticancer and antibacterial drugs, potentially serving as topoisomerase II and DNA-gyrase inhibitors (Scheme 1).

**Scheme 2:**
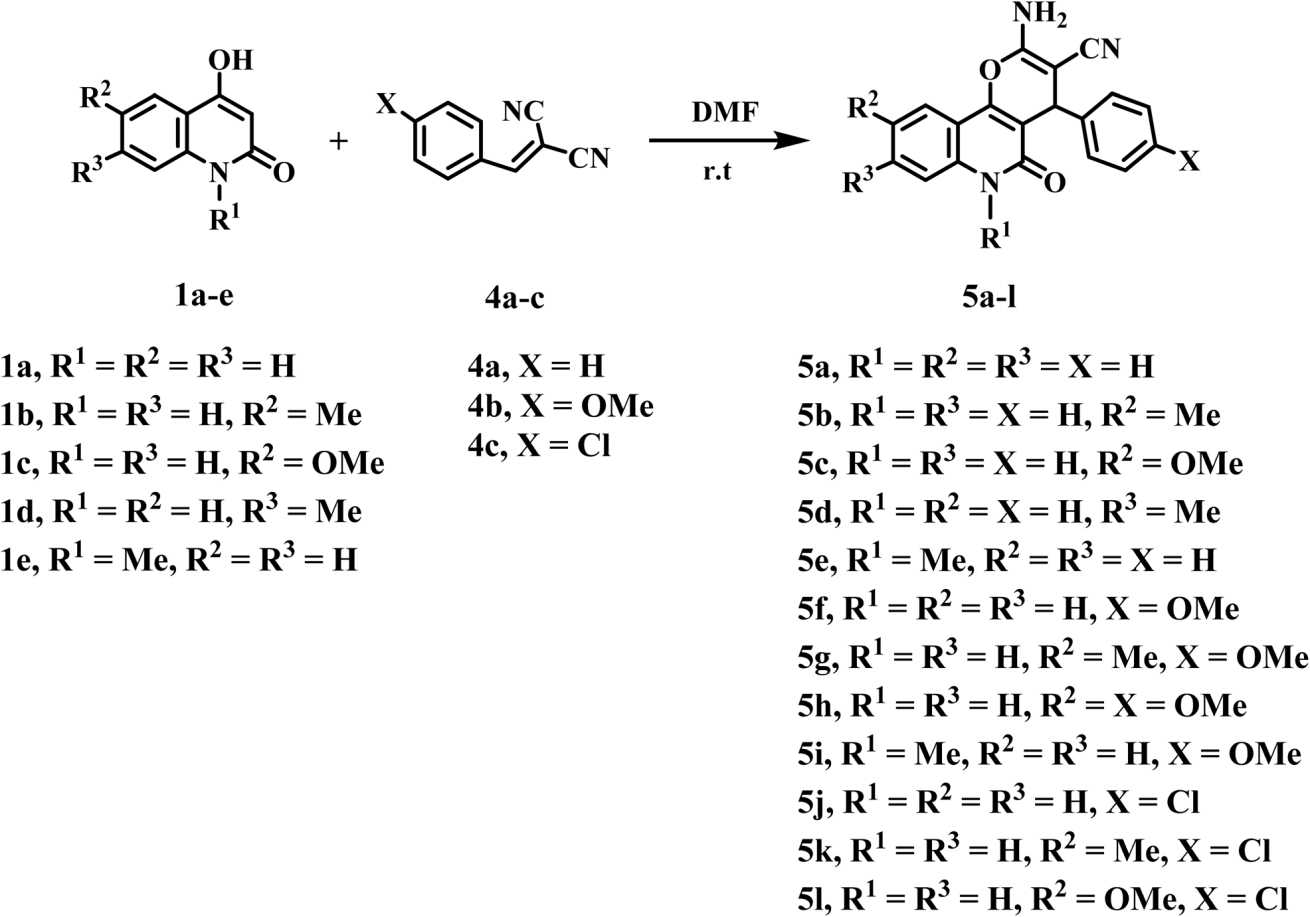
Synthesis of 2-amino-pyrano[3,2-*c*]quinoline-3-carbonitriles 5a–l.

Also, Kadu *et al.*, obtained the similar 4*H*-pyrano[3,2-*c*]quinoline-3-carbonitriles *via* three-component multicomponent reactions.^40^ In this manuscript, to prevent the possibility of side reactions, between quinolones-C-3 and aldehyde, we developed a new series of 2-amino-5-oxo-4-phenyl-5,6-dihydro-4*H*-pyrano[3,2-*c*]quinoline-3-carbonitrile derivatives 5a–l*via* direct interaction between 4-hydroxy-2-oxo-1,2-dihydroquinoline derivatives 1a–e and 2-benzylidenemalononitriles 4a–c (Scheme 2).^41^ The reaction was conducted under several conditions: ethanol/Et_3_N under reflux (method I), ethanol/K_2_CO_3_ (method II), and DMF with stirring at room temperature (method III) (Scheme 2). Nevertheless, when the reaction was conducted using method III, the optimal approach afforded substantial yields of products 5a–l with exceptional purity (65–92%). Additionally, all the reactions were carried out under the same conditions and through three different methods, but a higher yield was obtained when using method III. In general, across the three methods used, there was a clear effect on the yield percentage in the case of substitutes with electron-withdrawing group's 5j–l comparing with others obtained compounds 5a–i as shown in Table 1.

**Table 1:** Yields of 2-amino-6-methyl-4-(subst. phenyl)-5-oxo-5,6-dihydro-4*H*-pyrano-[3,2-*c*]quinoline-3-carbonitriles using methods I, II and III

Compound	Method I	Method II	Method III
5a	60%	70%	80%
5b	63%	77%	90%
5c	65%	78%	92%
5d	67%	80%	90%
5e	57%	66%	78%
5f	70%	70%	86%
5g	61%	77%	89%
5h	69%	79%	90%
5i	57%	67%	78%
5j	50%	65%	70%
5k	48%	63%	68%
5l	45%	61%	65%

**Fig. 3:**
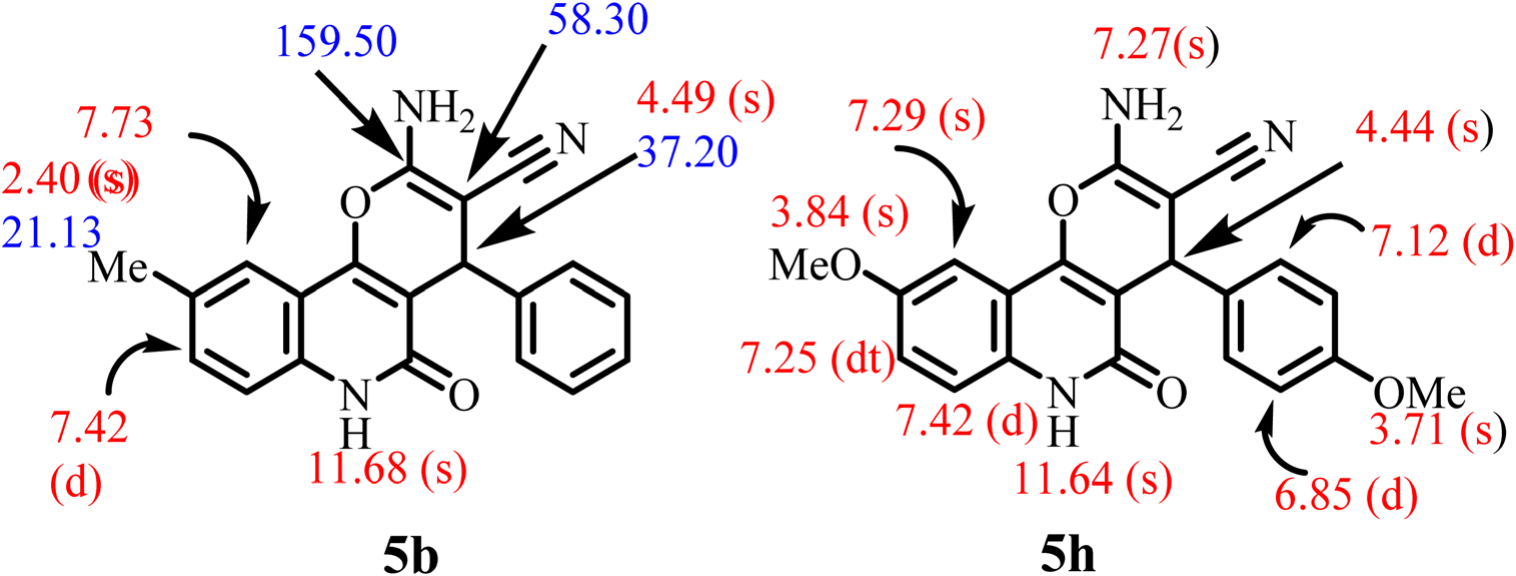
Distinctive carbons and protons of compounds 5b and 5h.

NMR spectroscopy, elemental analysis, and mass spectrometry were performed on all samples to validate the structures of our new compounds. For example, compound 5b was assigned as 2-amino-9-methyl-5-oxo-4-phenyl-5,6-dihydro-4*H*-pyrano[3,2-*c*]quinoline-3-carbonitrile and 5h, designated as 2-amino-9-methoxy-4-(4-methoxyphenyl)-5-oxo-5,6-dihydro-4*H*-pyrano[3,2-*c*]quinoline-3-carbonitrile (Fig. 3). The elemental analysis and mass spectrometry of 5b gave its molecular weight as *m*/*z* 329, with the molecular formula C_20_H_15_N_3_O_2_. The ^1^H NMR spectrum for 5b showed four singlet signals at *δ*_H_ = 2.40, 4.49, 7.73, and 11.68 ppm, which were assigned as CH_3_ group, H-4, H-10, and quinolinone-NH, respectively, in addition to aromatic protons. Another doublet signal at *δ*_H_ = 7.42 ppm (*J* = 8.4 Hz; 1H) as H-8. The NH_2_ protons resonated as a broad singlet at *δ*_H_ = 7.27 ppm, which appeared clearly in compound 5h (Fig. 3). As ^13^C NMR spectrum revealed the methyl carbon signals at *δ*_C_ = 21.13 (CH_3_) and 58.30 ppm (C-3), while the CH-pyran appeared as a singlet at *δ*_C_ = 37.20 ppm (C-4). The two signals resonated at *δ*_C_ = 160.80 and 159.50 ppm, were assigned as carbonyl group and pyran-C-2, respectively.

Compound 5h gives similar chemical shifts for all protons, unlike the aromatic ring, which contains a doublet of doublet system due to a 1,4-disubstituted benzene ring at *δ*_H_ = 7.12 (d, *J* = 8.6 Hz; 2H), 6.85 ppm (d, *J* = 8.6 Hz; 2H). Also, the ^1^H NMR spectrum for 5h clarified five singlet signals at *δ*_H_ = 11.64, 7.29, 7.27, 4.44 and 3.84 ppm, which were assigned as NH, quinolinone-H-10, amino-group, pyran-CH and methoxy group, respectively. However, the signal resonated at *δ*_H_ = 7.25 ppm, which appeared as dt-coupling with two different *J*-coupling (*J* = 8.7, 6.7 Hz, 1H), was assigned quinolinone-H-8, resulting two different coupling system, the first one as AB-coupling system with the quinolinone-H-7 and the second coupling as AX-coupling system with two methoxy-proton in the same direction due to the sp^3^-configuration of methoxy group. The ^13^C NMR spectrum of compound 5b confirmed its ^1^H NMR spectral data.

For example, the ^13^C NMR spectrum revealed the methyl carbon signals at *δ*_C_ = 21.13 (CH_3_) and 37.20 (C-4), in addition to the carbonyl group and cyano-group, which appeared as a singlet at *δ*_C_ = 160.80 and 115.78 ppm, respectively. Also, the pyran-C-2 and C-3 are resonated at *δ*_C_ = 159.50 and 58.30 ppm, respectively, by observed trends in *d* values for C-atoms in push–pull alkenes.^42^ However, elemental analyses and mass spectrometry for compound 5h revealed that it was formed through an interaction between one molecule of 4-hydroxy-6-methoxyquinolin-2(1*H*)-one 1c and another molecule of 2-(4-methoxybenzylidene)malononitrile 4b without any elimination, with the chemical formula C_21_H_17_N_3_O_4_ and molecular weight *m*/*z* = 375.

**Scheme 3:**
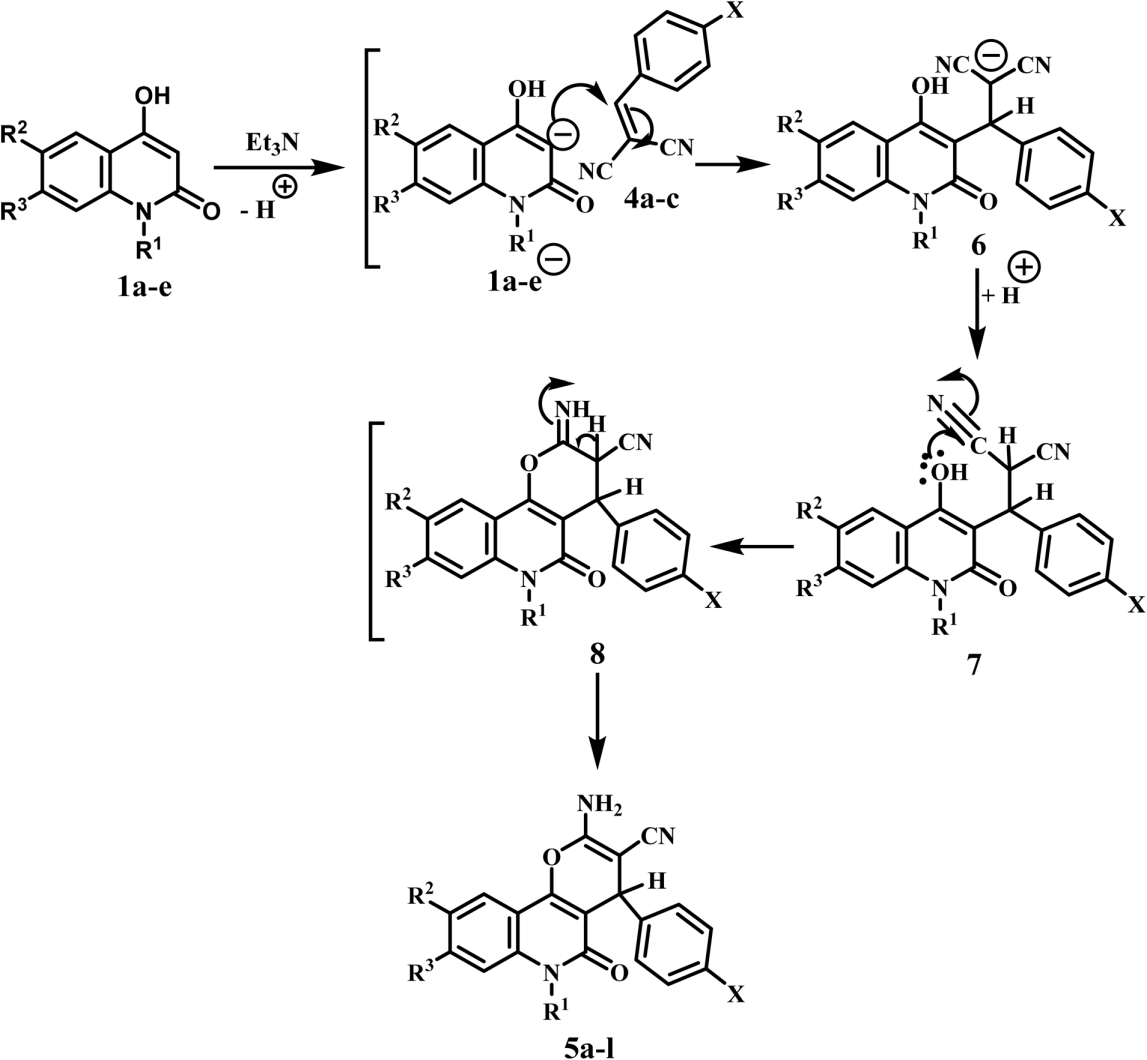
Suggested mechanism for pyrano[3,2-*c*]quinoline-3-carbonitriles 5a–l.

The proposed mechanism for the obtained products 5a–l begins with a nucleophilic attack of the active C-3, which is formed by triethylamine 1a–e, to bond in compound 4a–c*via* Michael addition to produce intermediates 6. Further inter-nucleophilic attack of the hydroxyl lone pair, followed by cyclization occurs and forms the intermediate 8. Finally, the rearrangement of the intermediate 8 gives the final products 5a–l (Scheme 3).

### 3.2. Biology

#### 3.2.1. Cell viability assay

The human mammary gland epithelium (MCF-10A) normal cell line was employed to evaluate the viability effects of novel derivatives 5a–l. The cell viability of 5a–l was assessed *via* the MTT assay following four days of incubation with 50 μM of each compound on MCF-10A cells.^33,34^ The results in Table 2 indicate that none of the investigated compounds exhibited cytotoxicity, and all hybrids maintained over 90% cell viability at a concentration of 50 μM.

**Table 2:** IC_50_ values of compounds 5a–l and erlotinib against four cancer cell lines

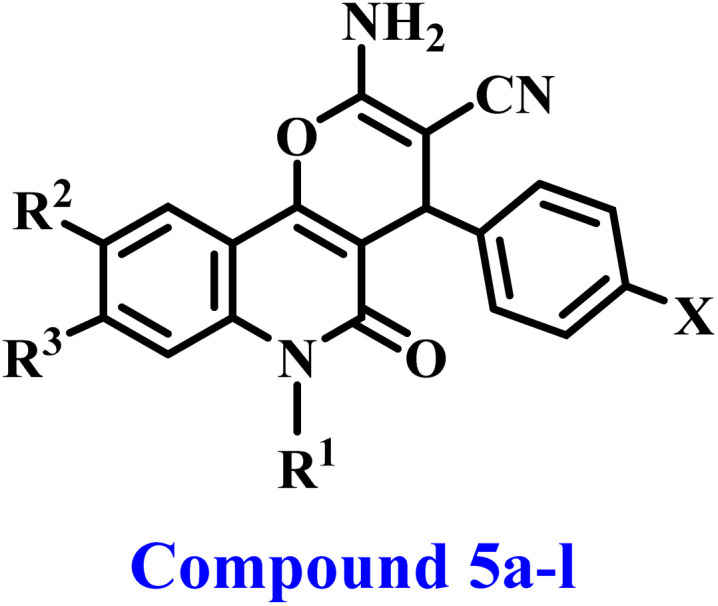										
Comp.	Cell viability (%)	R^1^	R^2^	R^3^	X	Antiproliferative activity IC_50_ ± SEM (nM)				
						A-549	MCF-7	Panc-1	HT-29	Average (GI_50_)
5a	92	H	H	H	H	32 ± 2	27 ± 2	32 ± 2	32 ± 2	31
5b	90	H	Me	H	H	48 ± 4	44 ± 4	48 ± 4	50 ± 5	48
5c	91	H	OMe	H	H	66 ± 6	59 ± 5	68 ± 6	70 ± 6	66
5d	93	H	H	Me	H	35 ± 3	31 ± 2	36 ± 3	37 ± 3	35
5e	90	Me	H	H	H	26 ± 2	24 ± 2	28 ± 2	27 ± 2	26
5f	92	H	H	H	OMe	54 ± 4	50 ± 4	56 ± 5	56 ± 5	54
5g	93	H	Me	H	OMe	71 ± 6	67 ± 6	74 ± 6	74 ± 6	72
5h	91	H	OMe	H	OMe	28 ± 2	26 ± 2	29 ± 2	29 ± 2	28
5i	90	Me	H	H	OMe	30 ± 2	27 ± 2	31 ± 2	33 ± 2	30
5j	91	H	H	H	Cl	43 ± 4	38 ± 3	44 ± 4	46 ± 4	43
5k	93	H	Me	H	Cl	39 ± 3	34 ± 3	42 ± 3	42 ± 3	39
5l	92	H	OMe	H	Cl	60 ± 5	56 ± 5	62 ± 5	63 ± 5	61
Erlotinib	N.D.	—	—	—	—	30 ± 3	40 ± 3	30 ± 3	30 ± 3	33

#### 3.2.2. Antiproliferative assay

The MTT assay was employed to evaluate the antiproliferative effects of targeting 5a–l, using erlotinib as a reference, against four human cancer cell lines: colon cancer (HT-29), pancreatic cancer (Panc-1), lung cancer (A-549), and breast cancer (MCF-7).^35,36^Table 2 presents each compound's median inhibitory concentration (IC_50_) and GI_50_ (mean IC_50_) against the four cancer cell lines.

Target 5a–l displayed significant antiproliferative efficacy, with GI_50_ values ranging from 26 nM to 72 nM, compared to the reference erlotinib (GI_50_ = 33 nM). Furthermore, all evaluated compounds showed pronounced sensitivity to the breast cancer (MCF-7) cell line relative to other cell lines examined. Compounds 5a, 5d, 5e, 5h, and 5i exhibited the highest antiproliferative potency, with GI_50_ values of 31, 35, 26, 28, and 30 nM, rendering these compounds (except 5d) more effective than erlotinib. Furthermore, these five derivatives exhibited superior efficacy to erlotinib against the MCF-7 cancer cell line, with IC_50_ values between 24 nM and 31 nM, while erlotinib's IC_50_ value is 40 nM.

Compound 5e (R^1^ = Me, R^2^ = R^3^ = X = H), N–Me derivative, was the most effective of all the synthesized derivatives, with a GI_50_ value of 26 nM. It was 1.3 times more effective than erlotinib (GI_50_ = 33 nM) against the four cancer cell lines that were tested. Furthermore, 5e exhibited an IC_50_ value of 24 nM against the MCF-7 breast cancer cell line, demonstrating a potency 1.7-fold greater than erlotinib (IC_50_ = 40 nM) against the MCF-7 cancer cell type. Also, compound 5e has marginally greater effectiveness than erlotinib against the other three cell lines, as shown in Table 2. These findings countered recent findings from related compounds, showing that *N*-alkyl derivatives had the lowest activity relative to derivatives containing a free nitrogen atom at quinoline position-1.^21^

The antiproliferative efficacy of compounds 5a–l is markedly influenced by the substitution pattern at positions one (*N* − 1) and six of the quinoline scaffold. For instance, compound 5a (R^1^ = R^2^ = R^3^ = X = H), a derivative containing a free nitrogen atom, had inferior efficacy as an antiproliferative agent compared to the *N*-methyl derivative, 5e (R^1^ = Me, R^2^ = R^3^ = X = H). Compound 5a had a GI_50_ value of 31 nM against four cancer cell lines, placing it fourth in activity compared to 5e (GI_50_ = 26 nM). An additional example is the 6-methyl derivative, compound 5b (R^2^ = Me, R^1^ = R^3^ = X = H), and the 6-methoxy derivative, 5c (R^2^ = OMe, R^1^ = R^3^ = X = H), both of which were demonstrated to be less efficacious than the unsubstituted derivative, 5a (R^1^ = R^2^ = R^3^ = X = H). Compounds 5b and 5c demonstrate IC_50_ values of 48 and 66 nM, respectively, which are 1.5 and 2.1 times less efficient than 5a (GI_50_ = 31 nM). The findings indicate that derivatives with an unsubstituted phenyl group of the quinoline moiety exhibit greater efficiency than those substituted with electron-donating methyl and methoxy groups.

Also, the substitution pattern at position 4 of the phenyl group in the pyran moiety may significantly influence the antiproliferative activity of these compounds. Compound 5h (R^1^ = R^3^ = H, R^2^ = X = OMe) exhibited the second highest activity, with a GI_50_ value of 26 nM, demonstrating a potency 1.25-fold greater than the reference erlotinib, which has a GI_50_ value of 33 nM. Compound 5h exhibits antiproliferative activity comparable to that of 5e, with both demonstrating more potency against the MCF-7 cell line than the reference erlotinib, Table 2.

Compounds 5c (R^1^ = R^3^ = H, R^2^ = OMe, X = H) and 5l (R^1^ = R^3^ = H, R^2^ = OMe, X = Cl) possess identical structural characteristics to compound 5h, differing just in the substitutions at the four position of the phenyl group of the pyran moiety, with hydrogen for 5c and chlorine for 5l. Compounds 5c and 5l exhibited GI_50_ values of 66 and 61 nM, respectively, demonstrating a potency reduction of 2.5- and 2.3-fold compared to 5h. This underscores the significance of the substitution pattern at this position on antiproliferative efficacy, with increasing activity in the sequence OMe > Cl > H.

Finally, the substitution of the 6-OMe group in compound 5h with a 6-Me group, as observed in compound 5g (R^1^ = R^3^ = H, R^2^ = Me, X = OMe), led to a notable reduction in antiproliferative activity. Compound 5g demonstrated a GI_50_ value of 72 nM, making it the least effective derivative and 2.6-fold less potent than 5h. These findings indicate that the methoxy group is more tolerated at the six-position for antiproliferative action than the methyl group.

#### 3.2.3. Assay for EGFR inhibitory activity

The EGFR-TK assay^37^ has been performed to evaluate the inhibitory efficacy of the most potent antiproliferative derivatives 5a, 5d, 5e, 5h, and 5i against EGFR, with results (IC_50_ values) presented in Table 3. The results from this *in vitro* assay were consistent with those from the antiproliferative assay. Compound 5e (R^1^ = Me, R^2^ = R^3^ = X = H), the most effective antiproliferative derivative, exhibited the highest efficiency as an EGFR inhibitor with an IC_50_ value of 71 nM, demonstrating 1.2-fold more potency than the reference medication erlotinib, which has an IC_50_ value of 80 nM. Compounds 5h (R^1^ = R^3^ = H, R^2^ = X = OMe) and 5i (R^1^ = Me, R^2^ = R^3^ = H, X = OMe) exhibited second and third-highest EGFR inhibitory activity, with IC_50_ values of 75 and 78 nM, respectively. Both compounds exhibit lower potency than 5e, although they remain more potent than the reference erlotinib, as indicated in Table 3.

**Table 3:** IC_50_ values of compounds 5a, 5d, 5e, 5h, and 5i against EGFR, BRAF^V600E^, and HER-2

Compound	EGFR inhibition IC_50_ ± SEM (nM)	BRAF^V600E^ inhibition IC_50_ ± SEM (nM)	HER-2 inhibition IC_50_ ± SEM (nM)
5a	84 ± 5	79 ± 5	36 ± 2
5d	87 ± 5	83 ± 5	39 ± 2
5e	71 ± 4	62 ± 3	21 ± 1
5h	75 ± 4	67 ± 3	23 ± 1
5i	78 ± 4	74 ± 4	31 ± 2
Erlotinib	80 ± 5	60 ± 3	—
Lapatinib	—	—	26 ± 1

Compound 5a (R^1^ = R^2^ = R^3^ = X = H), featuring a free nitrogen atom at position-1 of the quinoline moiety, exhibited lower efficacy as an EGFR inhibitor than the *N*-methyl derivative, compound 5e. Compound 5a demonstrated an IC_50_ value of 84 nM, rendering it 1.2-fold less potent than 5e and even less potent than the reference erlotinib (IC_50_ = 80 nM), suggesting that the presence of a free nitrogen atom in the quinoline moiety does not confer any benefits for the antiproliferative or anti-EGFR activities of this class of compounds. Compound 5d (R^1^ = R^2^ = X = H, R^3^ = Me) exhibited significant inhibitory activity as an EGFR inhibitor, with an IC_50_ value of 87 nM. These data indicated that compounds 5e, 5h, and 5i displayed significant antiproliferative activity and may function as EGFR inhibitors.

#### 3.2.4. Assay for BRAF^V600E^ inhibitory action

The *in vitro* anti-BRAF^V600E^ efficacy of compounds 5a, 5d, 5e, 5h, and 5i was evaluated.^38^ The enzyme assays indicated that the evaluated hybrids exhibited substantial BRAF^V600E^ inhibitory activity, with IC_50_ values between 62 and 83 nM, as presented in Table 3. In every instance, the IC_50_ of the examined compounds exceeds that of the reference erlotinib (IC_50_ = 60 nM).

Once again, compound 5e, the most effective antiproliferative and EGFR inhibitor, demonstrated superior efficacy as a mutant-BRAF (BRAF^V600E^) inhibitor, with an IC_50_ value of 62 nM, equivalent to the reference erlotinib (IC_50_ = 60 nM). Compounds 5h and 5i exhibited remarkable anti-BRAF^V600E^ inhibitory efficacy, with IC_50_ values of 67 and 74 nM, respectively. Compounds 5h and 5i displayed 1.1- and 1.3-fold reduced potency compared to the reference erlotinib as BRAF^V600E^ inhibitors. Although the IC_50_ values of the studied compounds as BRAF^V600E^ inhibitors are lower than their IC_50_ values as EGFR inhibitors, all investigated compounds had lesser potency as BRAF^V600E^ inhibitors than erlotinib, necessitating additional structural modifications to improve their efficacy against BRAF^V600E^.

#### 3.2.5. HER-2 inhibitory assay

Compounds 5a, 5d, 5e, 5h, and 5i were evaluated for their capacity to inhibit HER-2 by the kinase assay.^39^ The findings are displayed in Table 3. Lapatinib functioned as the reference drug.

The findings indicated that the investigated compounds markedly suppressed HER-2, exhibiting IC_50_ values between 21 and 39 nM, compared to lapatinib's IC_50_ of 26 nM. Compounds 5e and 5h exhibited the highest potency as HER-2 inhibitors, with IC_50_ values of 21 and 23 nM, respectively, surpassing the reference lapatinib, which has an IC_50_ value of 26 nM. Compounds 5a, 5d, and 5i exhibited HER-2 activity with IC_50_ values of 36, 39, and 31 nM, respectively, which are less efficient than the reference compound lapatinib. These findings indicated that compounds 5e and 5h may serve as lead compounds exhibiting significant antiproliferative activity, potentially operating as multi-targeted protein kinase inhibitors.

### 3.3. Molecular docking studies

**Fig. 4:**
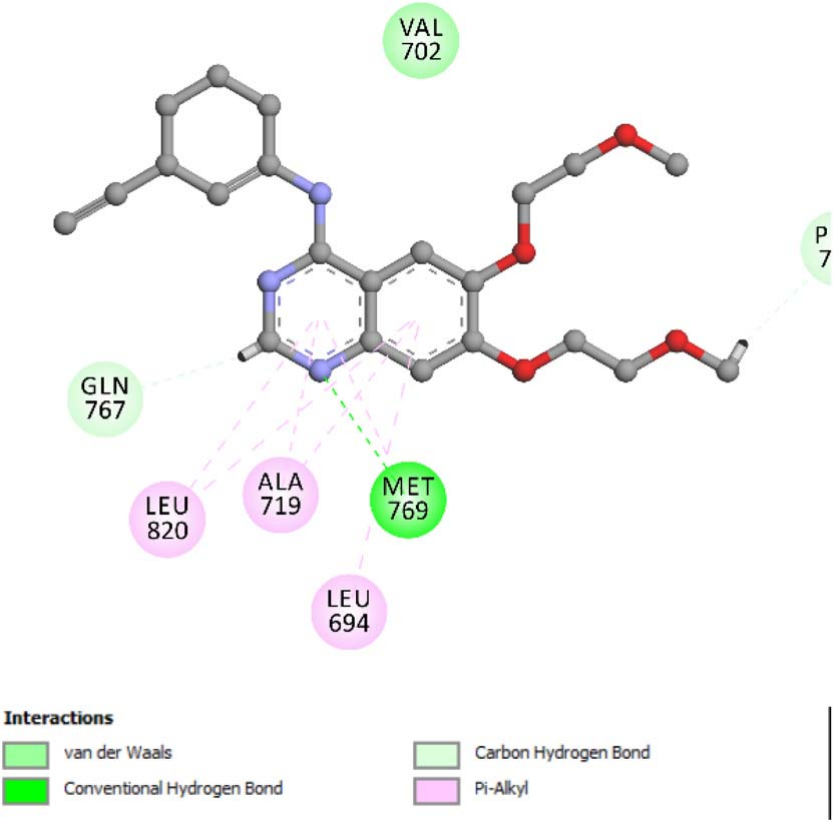
Two-dimensional schematic illustration depicting the binding orientation of erlotinib within the EGFR active site, highlighting key interactions that stabilize the ligand in the binding pocket.

To explore the molecular interactions of compound 5e with EGFR and HER-2 kinases, docking simulations were carried out utilizing the crystal structures of EGFR (PDB ID: 1M17) and HER-2 (PDB ID: 3PP0).^43^ These structural templates served as the basis for *in silico* analysis using Discovery Studio.^44^ Erlotinib and lapatinib were selected as benchmark inhibitors for EGFR and HER-2, respectively, to provide a comparative assessment of binding efficiency. Prior to docking, comprehensive preparation of the protein structures was conducted, including protonation state adjustments and energy minimization steps to optimize the geometry. The OPLS-AA force field was employed during the minimization phase to ensure stable ligand–protein conformations and improve docking precision. To confirm the robustness of the docking protocol, erlotinib, co-crystallized in the EGFR structure, was subjected to a re-docking procedure. The reproduced binding pose showed strong concordance with the crystallographic orientation, yielding a binding affinity score of −8.56 kcal mol^−1^ and a root-mean-square deviation (RMSD) of 0.95 Å. This level of agreement validated the reliability of the docking workflow. Importantly, the re-docked pose highlighted a key hydrogen bond between the pyrimidine moiety of erlotinib and the Met769 residue in the EGFR active site, a critical interaction for ligand stabilization (Fig. 4).

**Fig. 5:**
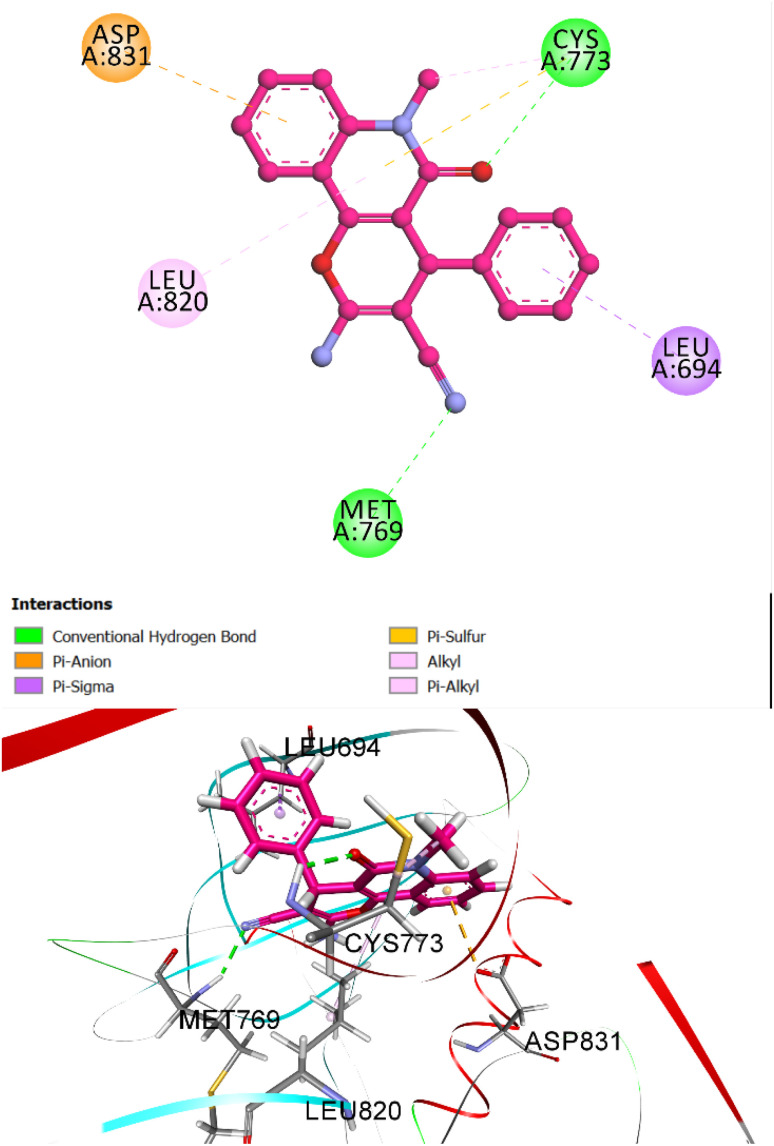
Two- and three-dimensional representations of the binding mode of compound 5e within the EGFR active site. The ligand forms a key hydrogen bond with Met769, along with additional interactions including π–alkyl contacts with Leu694 and Leu820, π–sulfur and π–anion interactions with Cys773 and Asp831, respectively, and a π–sigma interaction contributing to overall binding stability.

The docking evaluation of compound 5e within the ATP-binding cleft of EGFR yielded a favorable binding pose, reflected by a docking score of −7.76 kcal mol^−1^ and an RMSD value of 1.40 Å. These results support the reliability of the computational approach and suggest a meaningful correlation between the predicted binding conformation and potential biological activity. Compound 5e adopts a stable orientation in the active site. The cyano group of pyran ring forms a key conventional hydrogen bond with the Met769 residue, a hallmark interaction for effective EGFR inhibition. Additionally, the ligand is anchored through multiple non-covalent interactions, including π–alkyl interactions with Leu694 and Leu820. Also, the oxo group of quinolone ring forms essential hydrogen bond with Cys773. In addition, the aromatic system of quinolone moiety forms π–sulfur and π–anion contacts with Cys773 and Asp831, respectively. These contacts collectively enhance the overall binding stability. The spatial accommodation of compound 5e within the EGFR pocket is further reinforced by favorable hydrophobic interactions, supporting its firm positioning within the enzymatic cleft. The binding profile observed in the 2D, and 3D interaction diagrams (Fig. 5) illustrates the multifaceted interaction network contributing to ligand affinity and specificity.

**Fig. 6:**
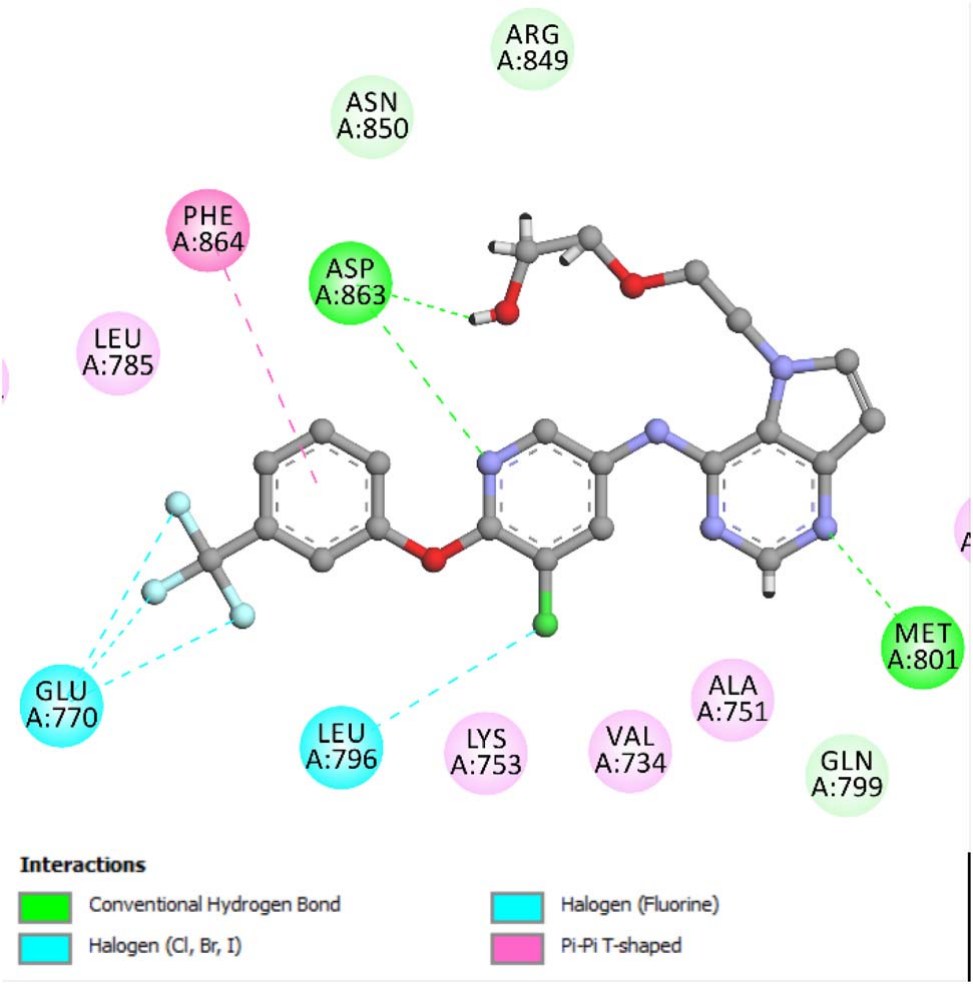
Two-dimensional schematic representation of the binding interactions formed by the validated co-crystallized ligand within the HER-2 active site. Key interactions include hydrogen bonds with Met801 and Asp863, π–π T-shaped stacking with Phe864, and halogen bonding with Glu770 and Leu796, all contributing to the stabilization of the ligand in the binding pocket.

To ensure the reliability of the docking approach for HER-2, the co-crystallized ligand was subjected to a re-docking procedure within the active site. The validation yielded a binding affinity score of −9.21 kcal mol^−1^ and an RMSD of 1.38 Å, indicating strong agreement with the experimentally observed binding pose and confirming the robustness of the docking methodology. The re-docking analysis revealed a key hydrogen bond between the pyrimidine nitrogen of the ligand and the Met801 residue, a critical interaction that contributes to anchoring the ligand within the active site. Additional stabilization was provided through a hydrogen bond between the pyridine nitrogen and Asp863, reinforcing the binding affinity. Furthermore, the simulation identified supplementary non-covalent interactions, including an additional hydrogen bond with Met801, a π–π T-shaped interaction involving Phe864, and halogen bonds with Glu770 and Leu796. These interactions collectively contribute to a stable and well-oriented ligand conformation within the HER-2 binding pocket (Fig. 6).

**Fig. 7:**
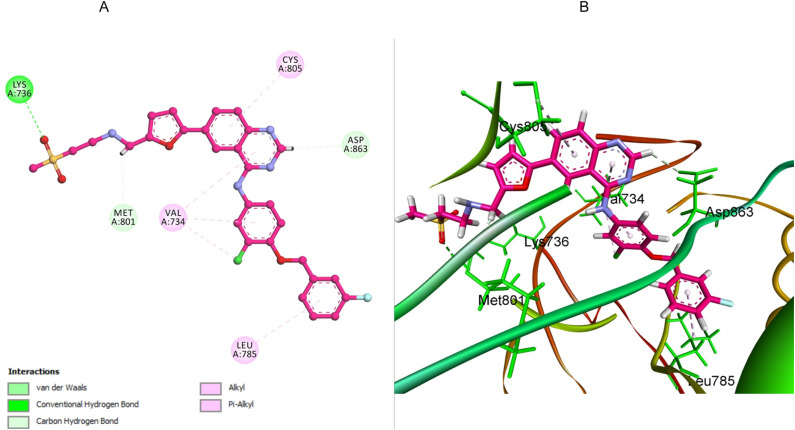
(A) 2D-docking representation of lapatinib within the HER-2 active site, (B) 3D representation, highlighting key interactions including hydrogen bonding with Lys753, carbon–hydrogen bonds with Met801 and Asp863, hydrophobic contacts with Leu785, and π–alkyl interactions with Val734, all contributing to stable conformation and inhibitory activity.

The binding behavior of lapatinib, employed as a reference inhibitor in the *in vitro* evaluation of HER-2, was examined through molecular docking studies. The results revealed a stable binding conformation, with a calculated binding energy of −8.64 kcal mol^−1^ and an RMSD of 1.01 Å, suggesting effective accommodation within the HER-2 active site. A key stabilizing interaction was observed between the sulfone oxygen atoms of lapatinib and the Lys736 residue, forming a crucial hydrogen bond. This was complemented by carbon–hydrogen bonding interactions with both Met801 and Asp863, which further reinforced the positioning. Additionally, lapatinib established extensive hydrophobic interactions, most notably with Leu785, forming a compact hydrophobic environment that enhances overall stability. The presence of π–alkyl interactions with Val734 contributed to the formation of a robust interaction network, securing lapatinib firmly within the HER-2 binding pocket. These collective interactions provide structural insights into the strong binding affinity and inhibitory potential of lapatinib against HER-2 (Fig. 7).

**Fig. 8:**
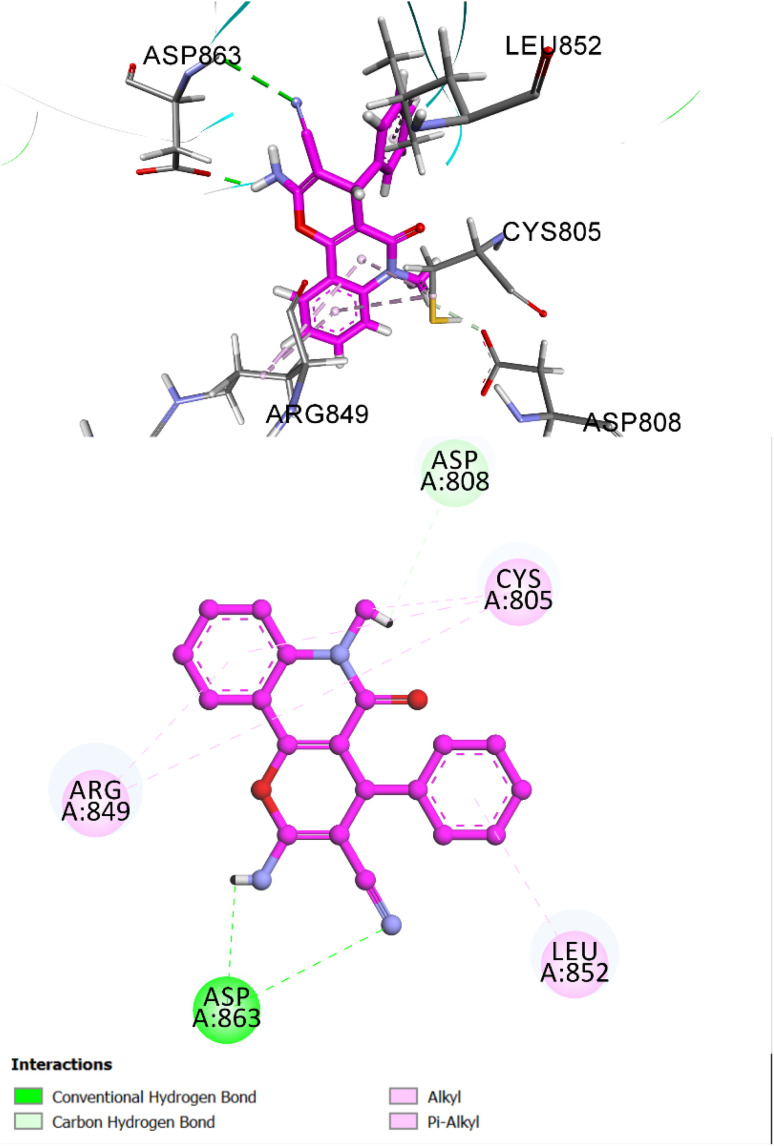
Two- and three-dimensional docking models showing the binding interactions of compound 5e within the HER-2 active site. Key interactions include a conventional hydrogen bond with Asp863, carbon–hydrogen bonding with Asp808, and π–alkyl contacts with Arg849, Leu852, and Cys805. These interactions collectively support stable orientation and potential inhibitory activity against HER-2.

The molecular docking of compound 5e within the HER-2 active site revealed a favorable binding profile, with a calculated binding energy of −7.84 kcal mol^−1^ and an RMSD value of 1.37 Å. These values indicate a stable ligand conformation and reliable fit within the binding pocket. Compound 5e demonstrated a well-anchored pose. The cyano and amino groups of pyran moiety stabilize compound 5e through a conventional hydrogen bond with Asp863. Additional stabilization arose from carbon–hydrogen bonding with Asp808, as well as, the quinolone moiety forms multiple π–alkyl interactions involving residues such as Arg849, and Cys805. These interactions contribute to the formation of a robust interaction network within the binding site. The 3D docking visualization highlights the deep insertion of compound 5e into the HER-2 binding cavity, where it forms several essential contacts that enhance its binding affinity and structural integrity. These interactions collectively suggest that compound 5e may serve as a promising scaffold for HER-2 inhibition (Fig. 8).

These molecular interactions observed in both EGFR and HER-2 highlight the structural compatibility and binding strength of compound 5e, supporting its potential as an effective kinase inhibitor. In conclusion, compound 5e demonstrated stable and favorable binding within both targets, making it a promising lead candidate for the development of EGFR/HER-2-targeted anticancer therapies.

### 3.4. Structural activity relationship (SAR) analysis

The following are some key-points outline the SAR of the newly synthesized compounds 5a–l
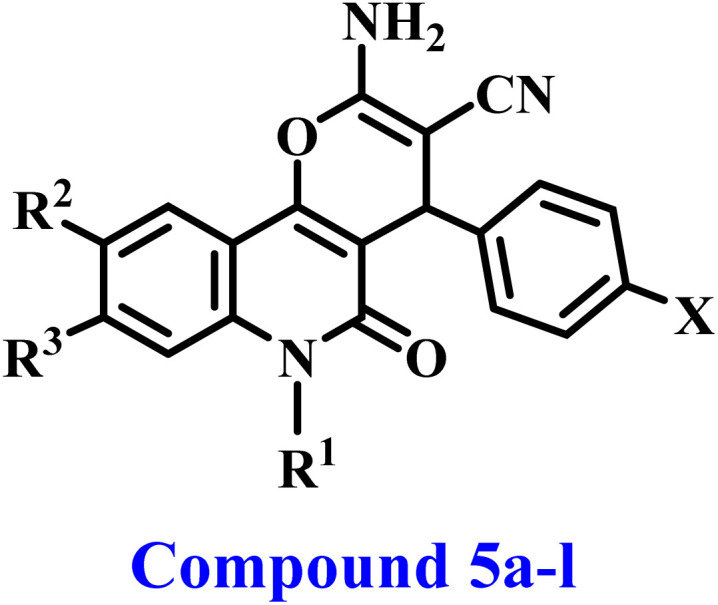


(1) The cyano group of pyran ring forms a key conventional hydrogen bond with the Met769 residue, a crucial interaction for effective EGFR inhibition.

(2) Additionally, the cyano and amino groups of pyran moiety stabilize compound 5e within HER-2 active site through a conventional hydrogen bond with Asp863 as well as carbon–hydrogen bonding with Asp808.

(3) The oxo group of quinolone ring forms essential hydrogen bond with Cys773 which stabilize 5e with EGFR binding site.

(4) The quinolone moiety forms π–sulfur and π–anion contacts with Cys773 and Asp831, respectively in EGFR binding sites, while forms multiple π–alkyl interactions involving residues such as Arg849, and Cys805 in HER-2 active site.

(5) Quinoline derivatives with an unsubstituted phenyl group demonstrate superior efficacy compared to those substituted with electron-donating methyl and methoxy groups.

(6) The substitution pattern of the X group (OMe, H, and Cl) significantly influences activity, with the following order of increasing activity: OMe > Cl > H.

## 4. Conclusion

This study involved the design, synthesis, and biological evaluation of a novel series of 2-amino-pyrano[3,2-*c*]quinoline-3-carbonitriles (5a–l) as multi-target inhibitors with significant antiproliferative activity. Compounds 5e and 5h had the most potent antiproliferative activities (GI_50_ = 26 and 28 nM, respectively), exceeding the efficacy of erlotinib and lapatinib by combined inhibition of EGFR and HER-2. The investigation of structure–activity relationships emphasized the essential function of the quinoline moiety and electron-donating substituents in strengthening activity. The multi-target profile (EGFR/HER-2/BRAF^V600E^ inhibitors) of these compounds highlights their potential as adaptable therapeutic agents for cancer treatment. Docking simulations identified compound 5e as a strong dual-target inhibitor for EGFR and HER-2. Its favorable binding interactions and affinity suggest its potential as an effective therapeutic agent. Additional research is needed to evaluate their *in vivo* effectiveness, pharmacokinetics, and toxicity. Future investigations will be focusing on enhancing BRAF^V600E^ inhibition while preserving the anticancer efficacy of the compounds. Furthermore, these lead compounds will be presented to kinase profiling experiments.

In summary, these quinoline-based compounds constitute a promising category of multi-targeted inhibitors with considerable potential for the development of novel therapeutic medicines for cancer, a serious global health concern. The multi-EGFR/HER-2/BRAF^V600E^ inhibition mechanism puts these compounds as adaptable candidates for advanced development in medicinal chemistry and drug discovery pipelines.

## Author contributions

Bahaa G. M. Youssif, Essmat M. El-Sheref, and Aliaa M. Mohassab: conceptualization, methodology, writing, editing and revision. Safwat M. Rabea: writing, editing and revision. S. Bräse: writing and editing. Lamya H. Al-Wahaibi: funding requirements, editing and revision.

## Conflicts of interest

The authors reported no potential conflicts of interest(s).

## Supplementary Material

RA-015-D5RA04276C-s001

## Data Availability

Samples of compounds 5a–l are available from the authors. Supplementary information is available. See DOI: https://doi.org/10.1039/d5ra04276c.
