# Supplementary material for: Design, synthesis, and antiproliferative activity of new 2-amino-pyrano[3,2-c]quinoline-3-carbonitriles as potential EGFR, BRAFV600E, and HER-2 inhibitors
Source: RSC Adv. 2025 Oct 13;15(45):38229–42. doi: 10.1039/d5ra04276c (PMC12517401; doi:10.1039/d5ra04276c)
Supplement: RA-015-D5RA04276C-s001 [file RA-015-D5RA04276C-s001.pdf]

Supplementary Information (SI) for RSC Advances.
This journal is © The Royal Society of Chemistry 2025

Design, synthesis, and antiproliferative activity of new 2-amino-pyrano[3,2-c]quinoline-3-carbonitriles as multi-targeted protein kinase inhibitors

Lamya H. Al-Wahaibi[1], Aliaa M. Mohassab[2], Safwat M. Rabea[2], Bahaa G. M. Youssif[3], Stefan Bräse[4], Essmat M. El-Sheref[5]

[1]Department of Chemistry, College of Sciences, Princess Nourah bint Abdulrahman University, Riyadh 11671, Saudi Arabia; [2]Department of Medicinal Chemistry, Faculty of Pharmacy, Minia University, Minia 61519, Egypt; [3]Department of Pharmaceutical Organic Chemistry, Faculty of Pharmacy, Assiut University, Assiut 71526, Egypt; [4]Institute of Biological and Chemical Systems, IBCS-FMS, Karlsruhe Institute of Technology, 76131 Karlsruhe, Germany; [5]Chemistry Department, Faculty of Science, Minia University, El Minia, 61519 Egypt.

**To whom correspondence should be addressed:*

Bahaa G. M. Youssif, Ph.D. Pharmaceutical Organic Chemistry Department, Faculty of Pharmacy, Assiut University, Assiut 71526, Egypt.

Tel.: (002)-01098294419

E-mail address: bgyoussif2@gmail.com

Essmat M. El-Sheref, Ph.D. Chemistry Department, Faculty of Science, Minia University, El Minia 61519, Egypt.

Tel.: (002)-01064890489

Stefan Bräse

Institute of Biological and Chemical Systems, IBCS-FMS, Karlsruhe Institute of Technology, 76131 Karlsruhe, Germany. E-mail: braese@kit.edu

Spectral data for compound 5a

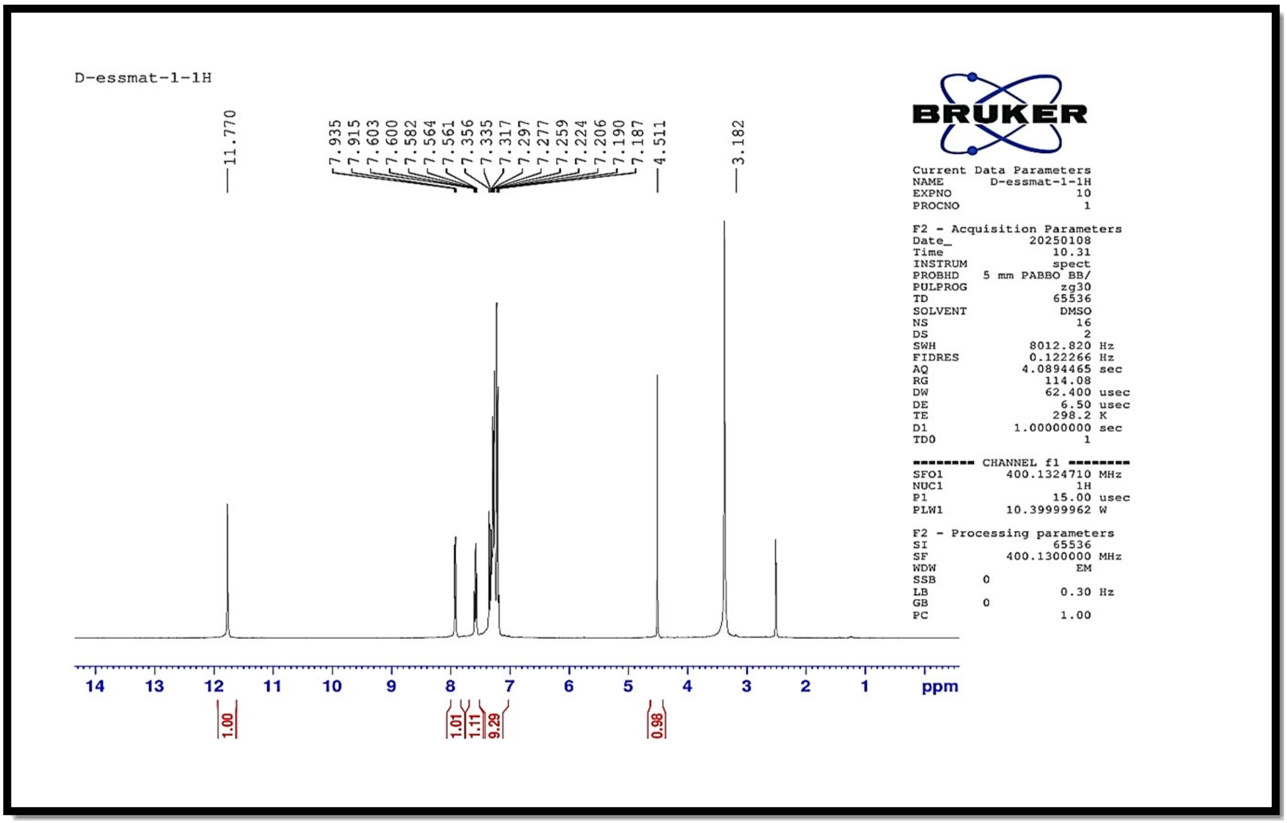

S1. ^{1}H-NMR spectrum for compound **5a**

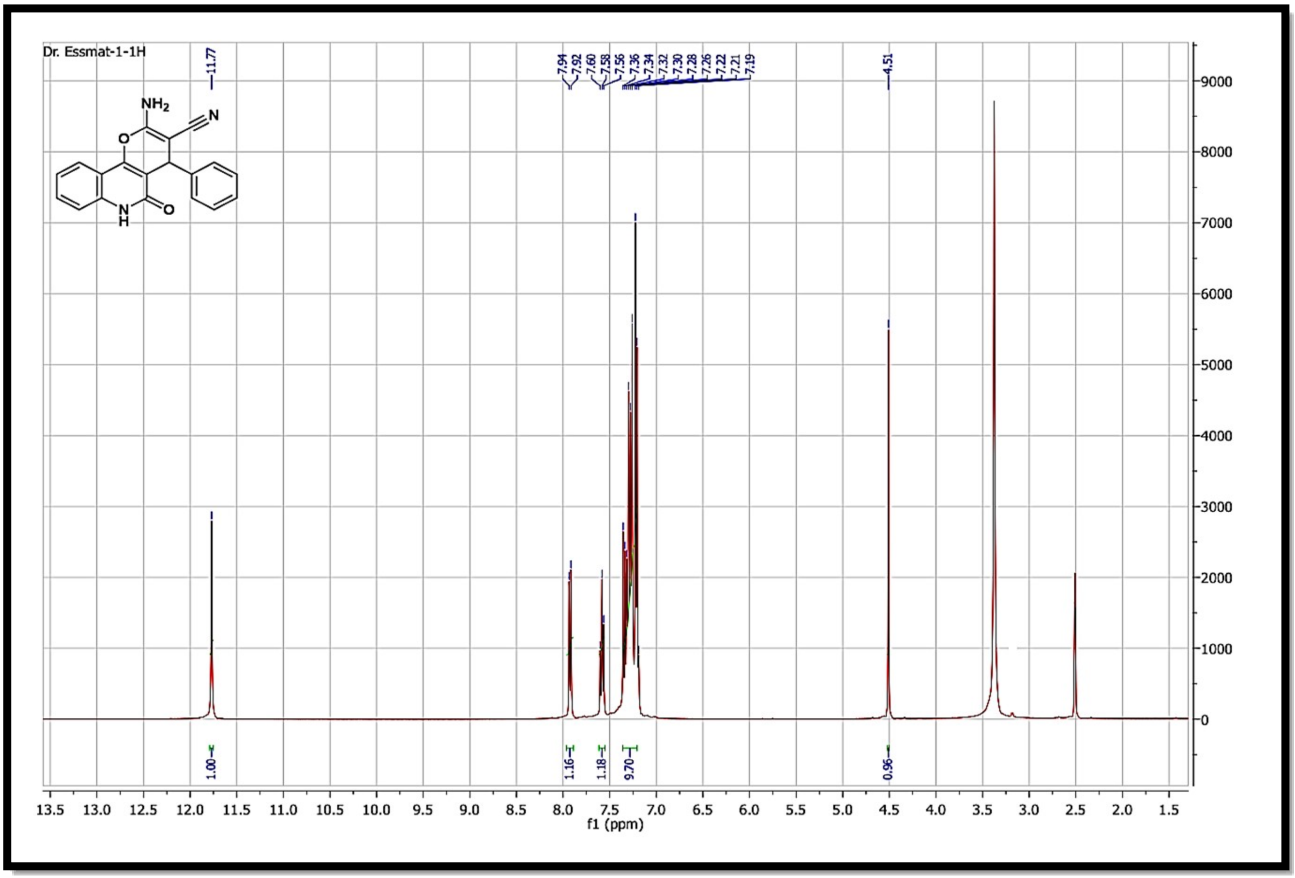

S2. Part of the ^{1}H-NMR spectrums for compound **5a**

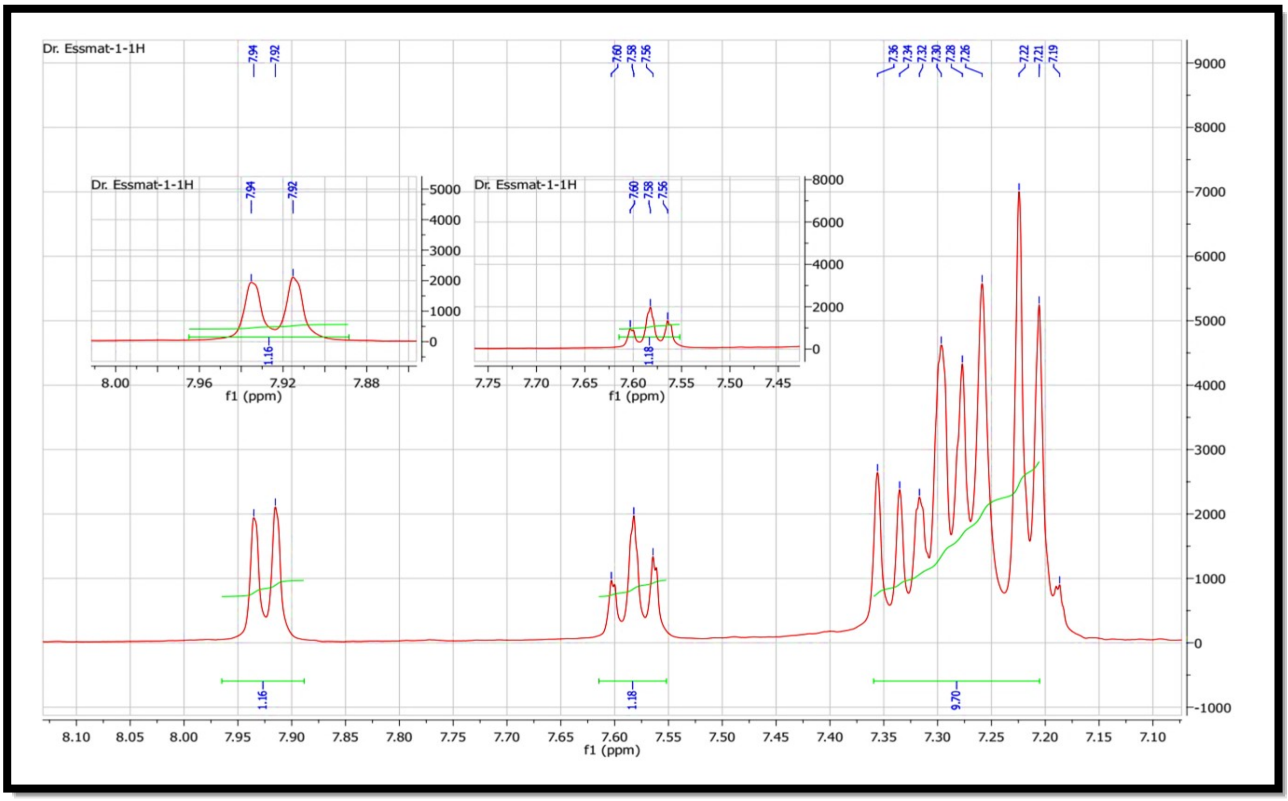

S3. Part of the ^{1}H-NMR spectrums for compound **5a**

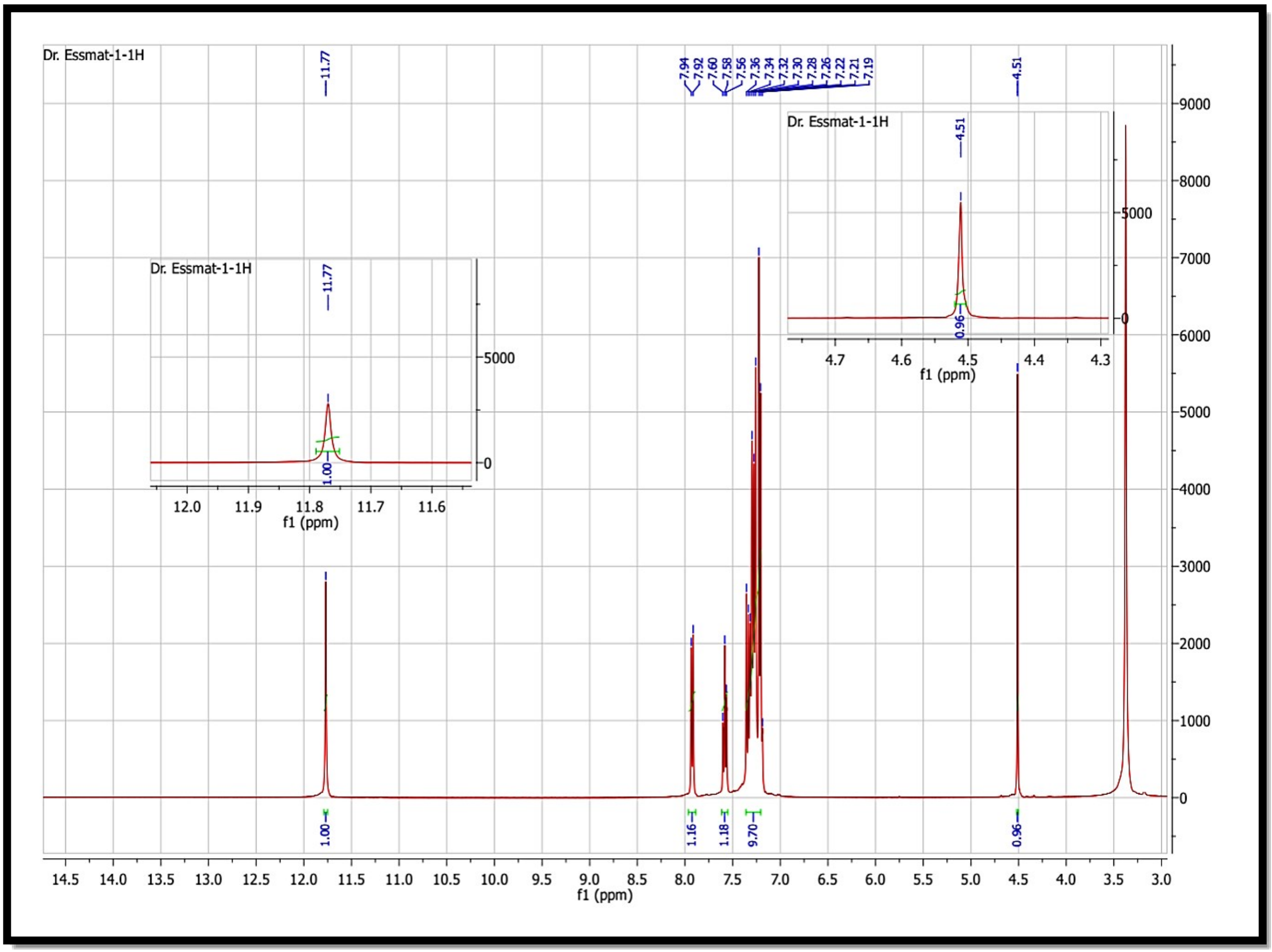

S4. Part of the ^{1}H-NMR spectrums for compound **5a**

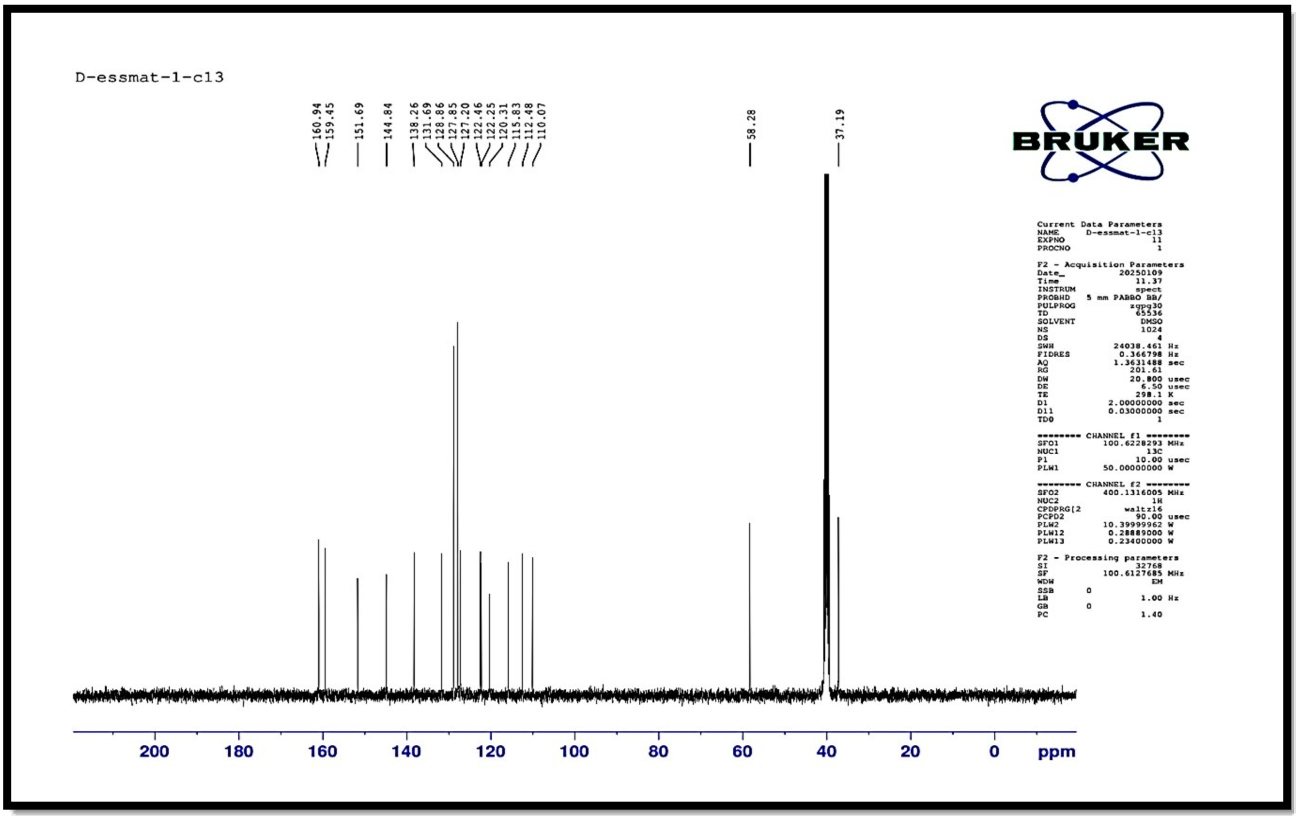

S5. ^{13}C-NMR spectrums for compound **5a**

Spectral data for compound 5b

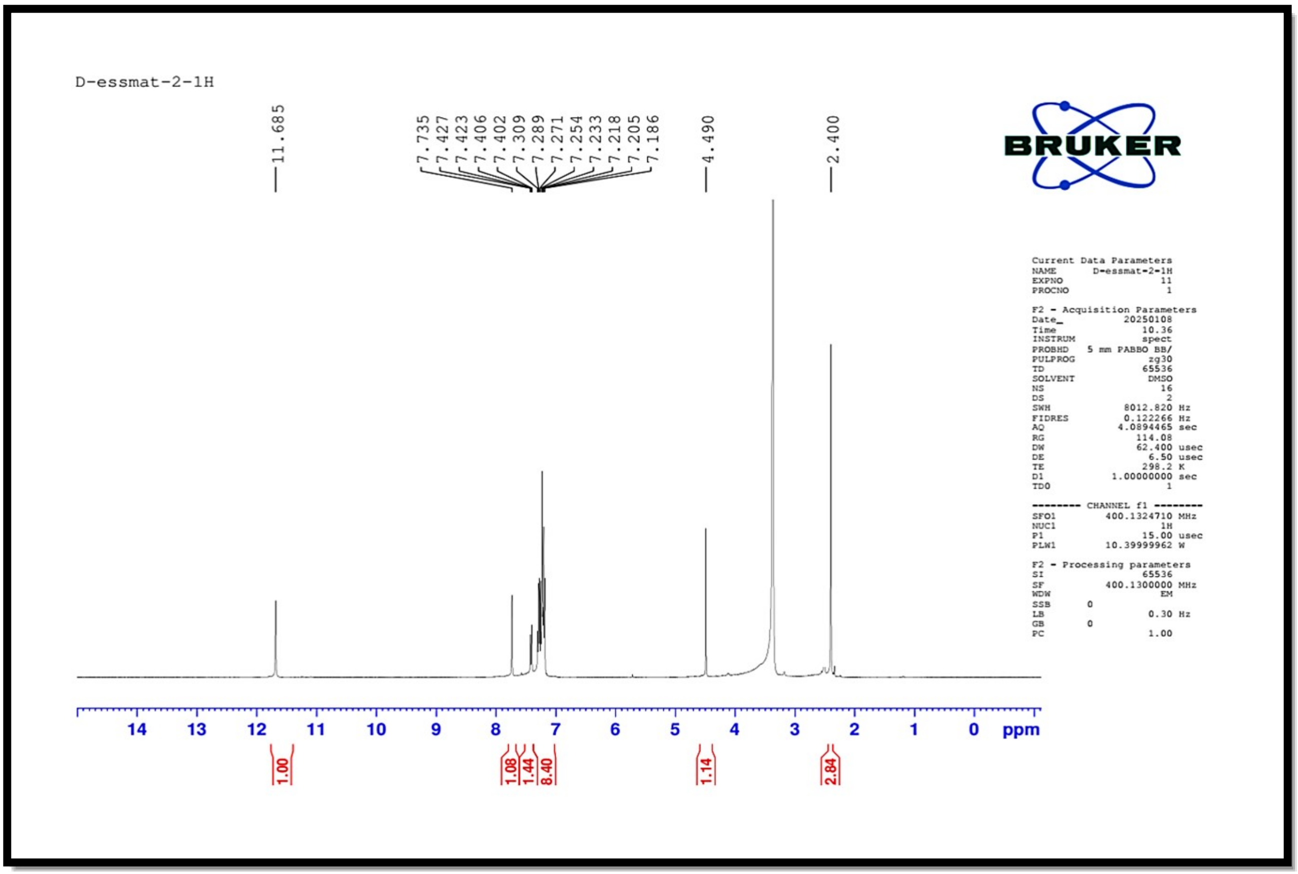

S6. ^{1}H-NMR spectrums for compound **5b**

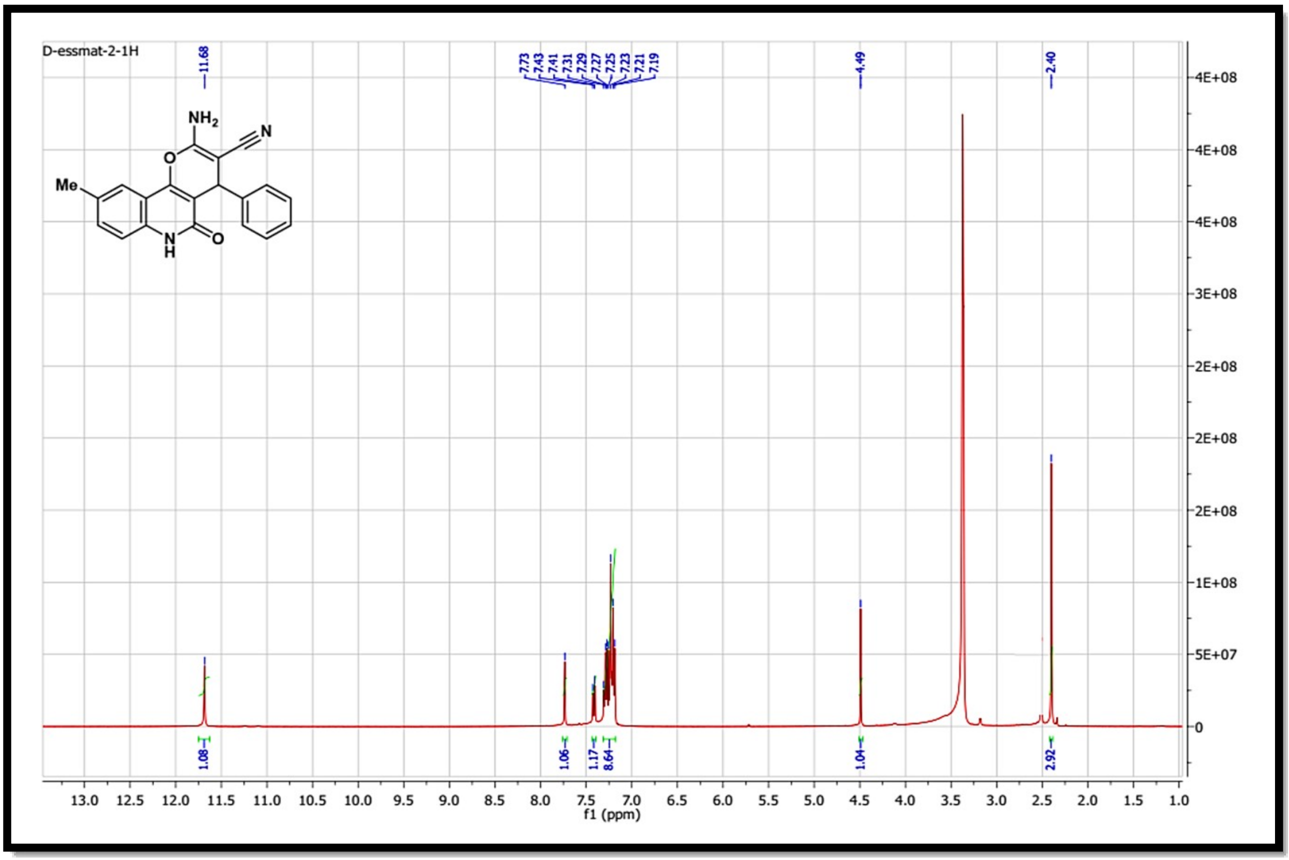

S7. Part of the ^{1}H-NMR spectrums for compound **5b**

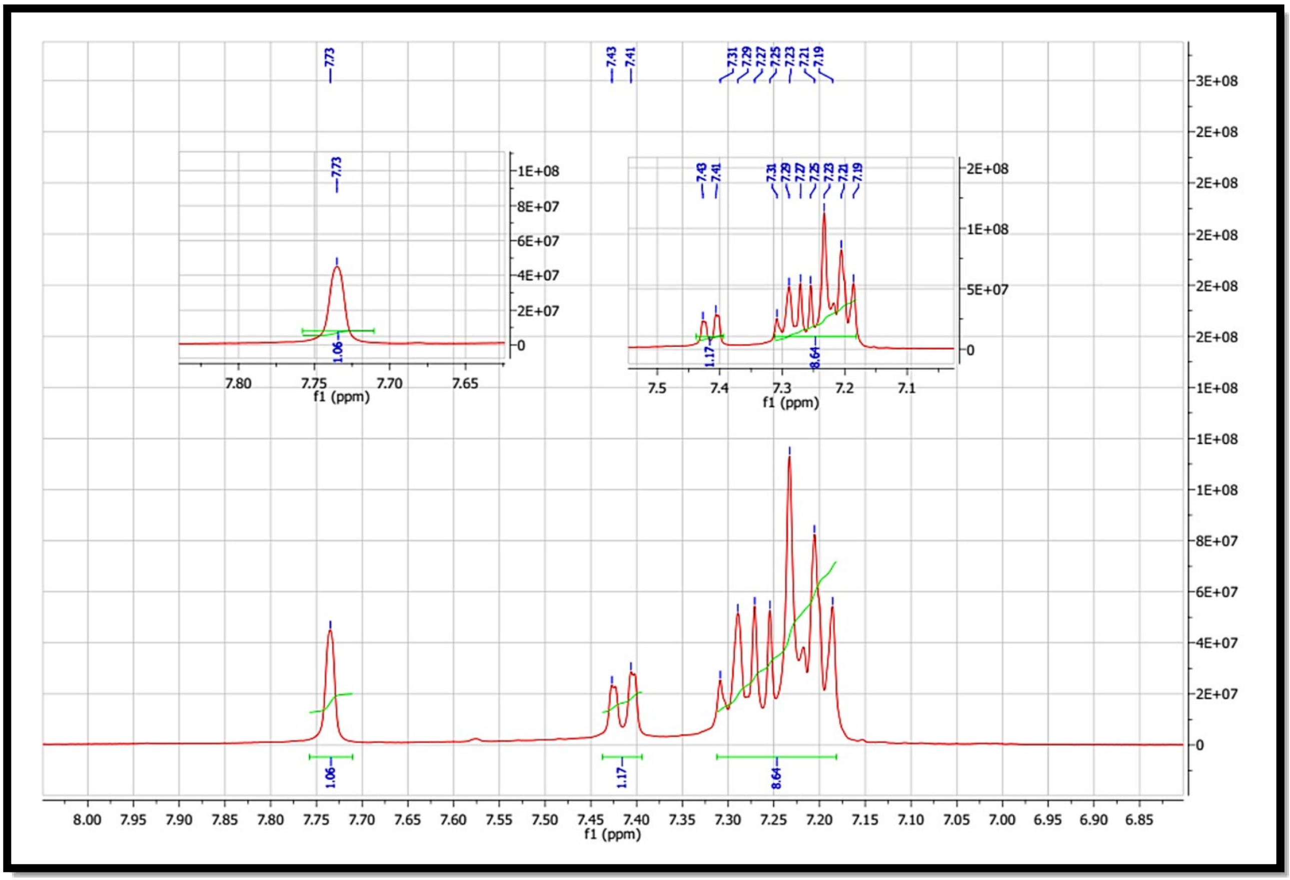

S8. Part of the ^{1}H-NMR spectrums for compound **5b**

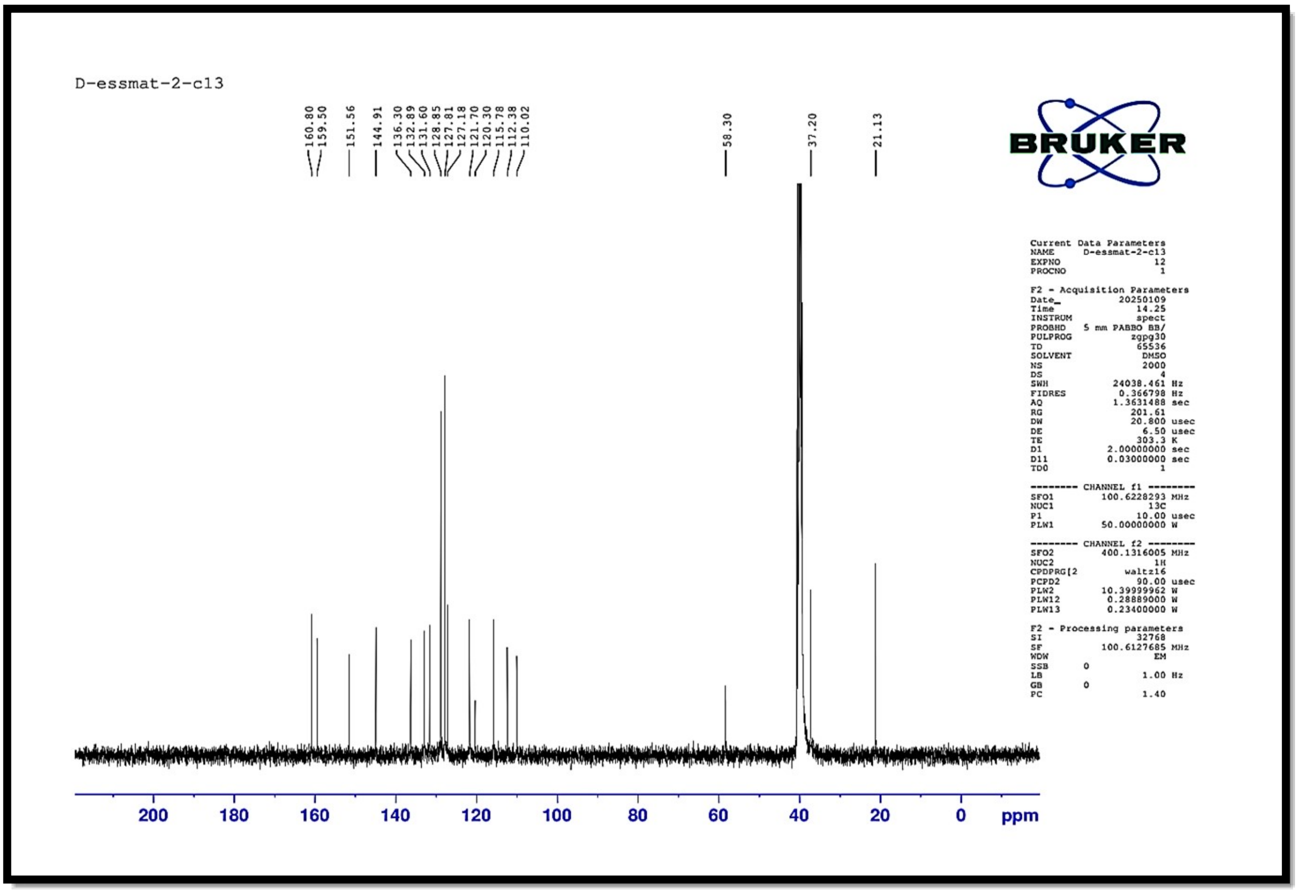

S9. ^{13}C-NMR spectrums for compound **5b**

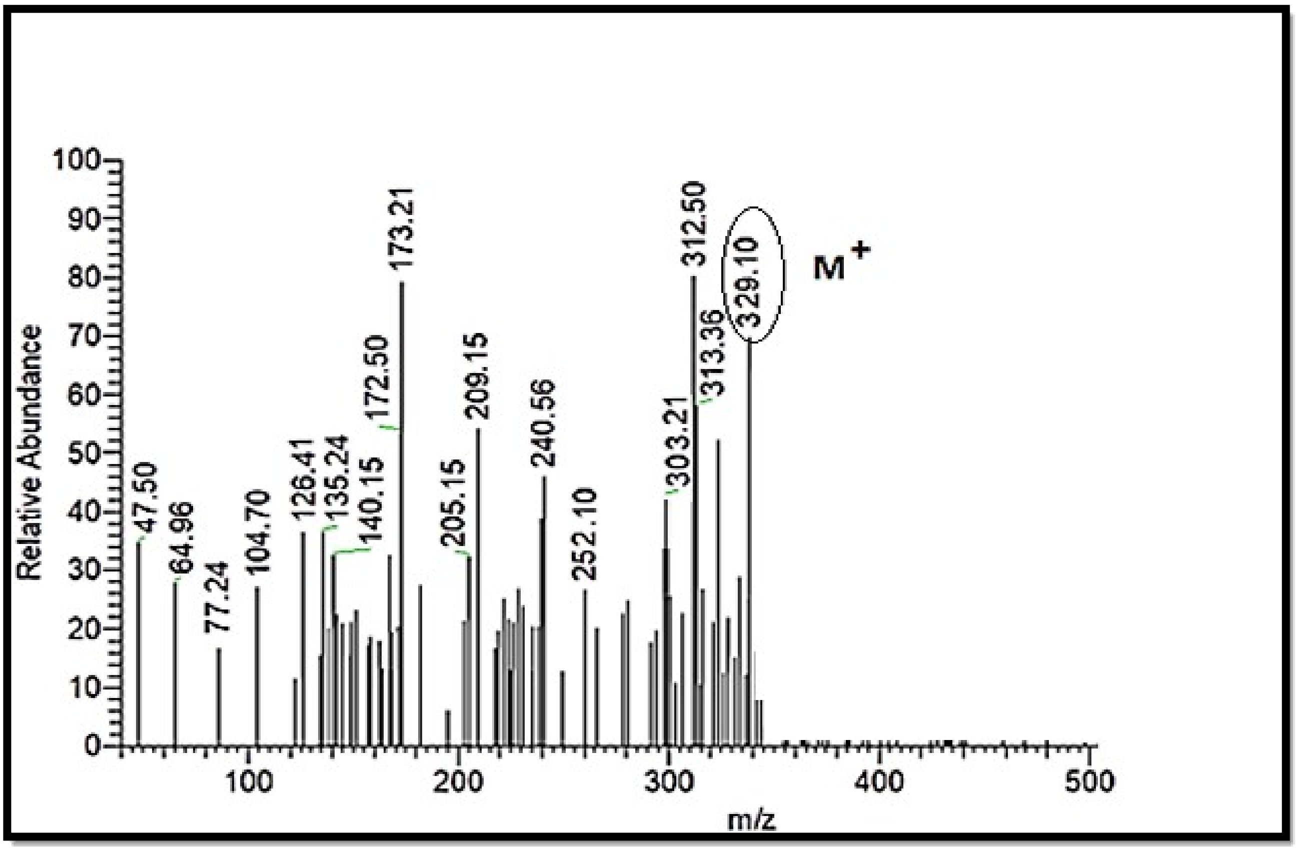

S10. Mass spectrometry for compound **5b**

Spectral data for compound 5c

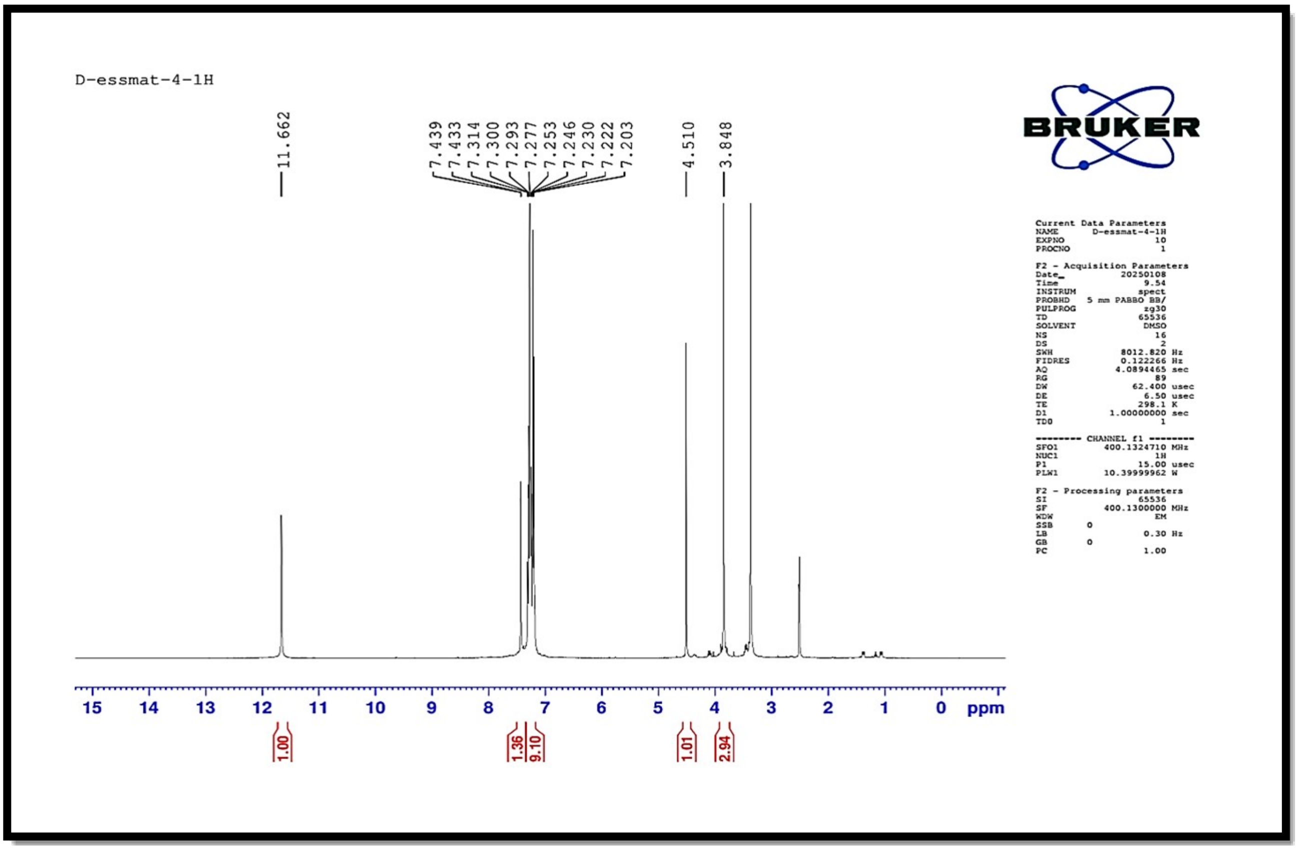

S11. ^{1}H-NMR spectrums for compound **5c**

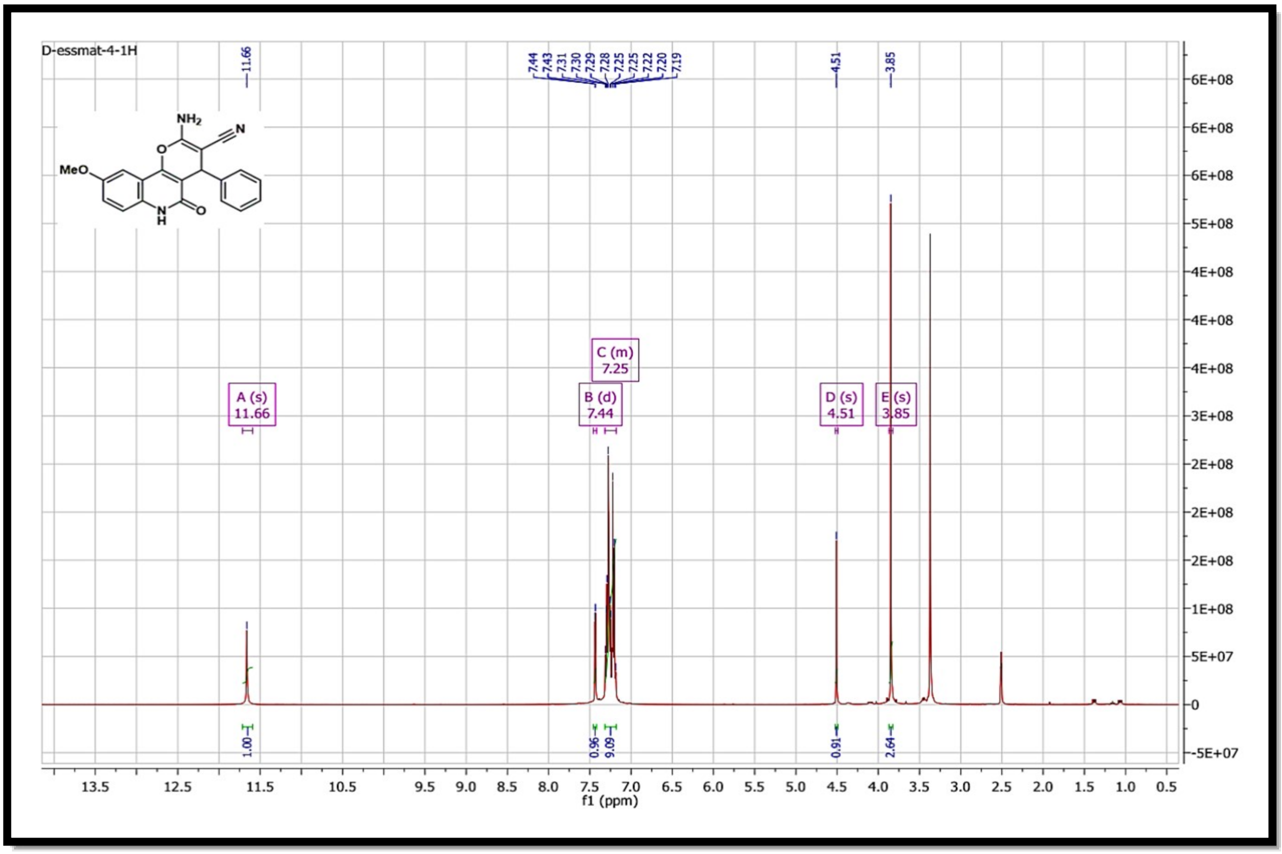

S12. Part of the ^{1}H-NMR spectrums for compound **5c**

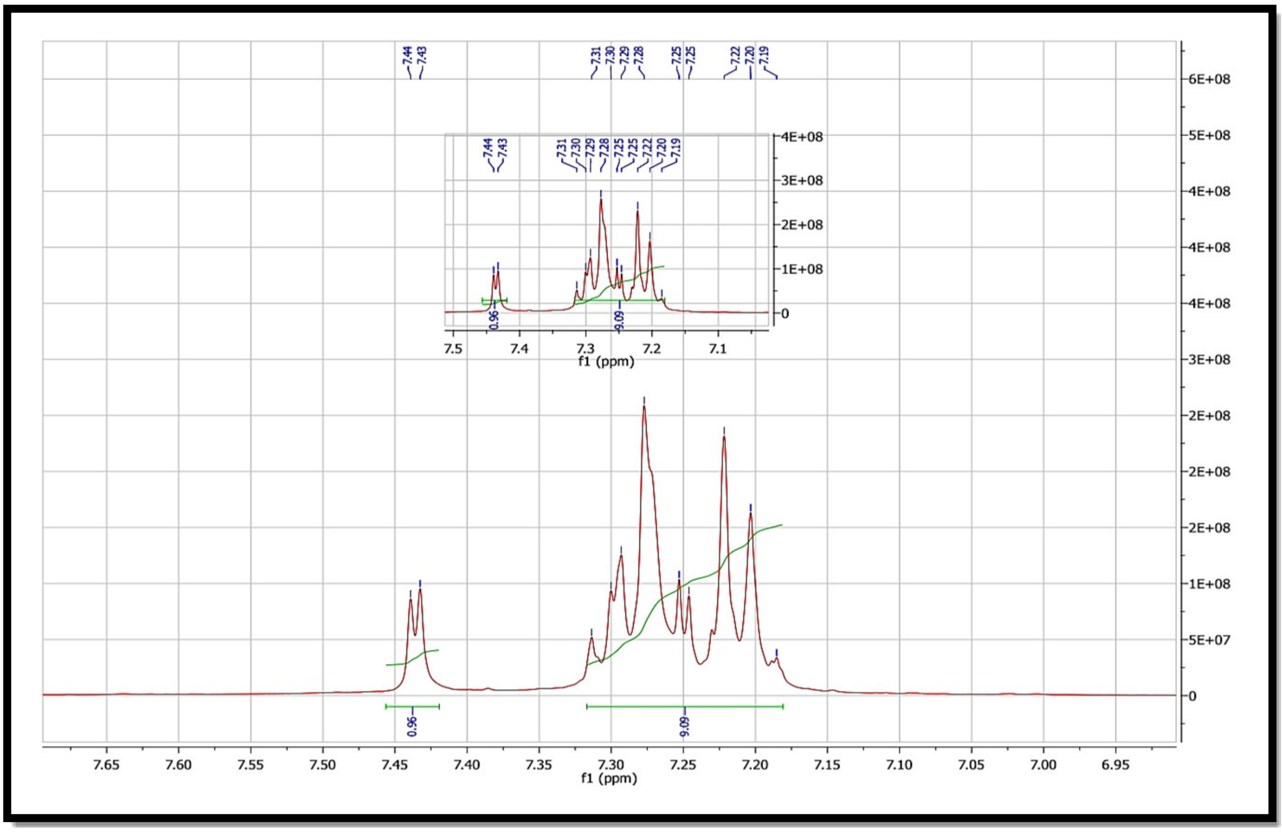

S13. Part of the ^{1}H-NMR spectrums for compound **5c**

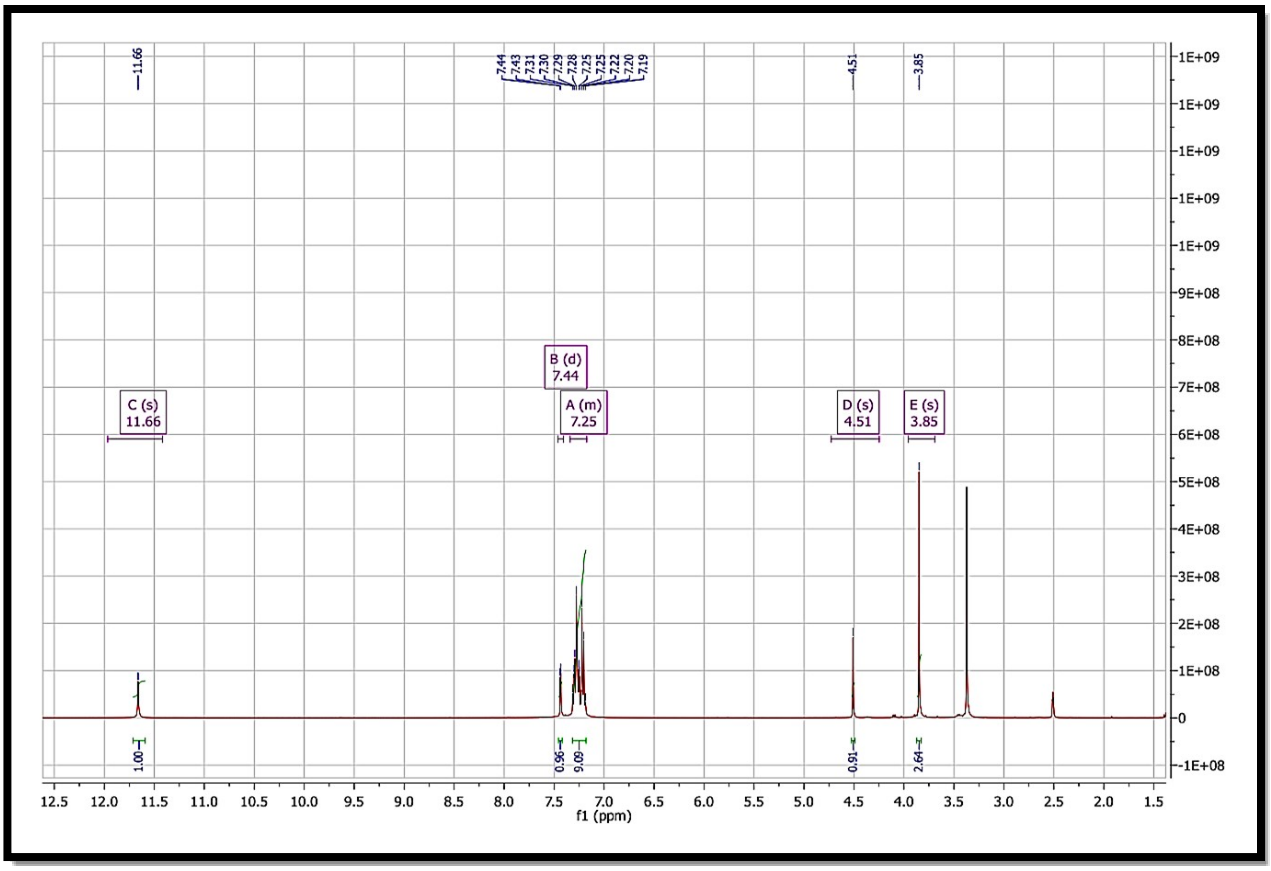

S14. Part of the ^{1}H-NMR spectrums for compound **5c**

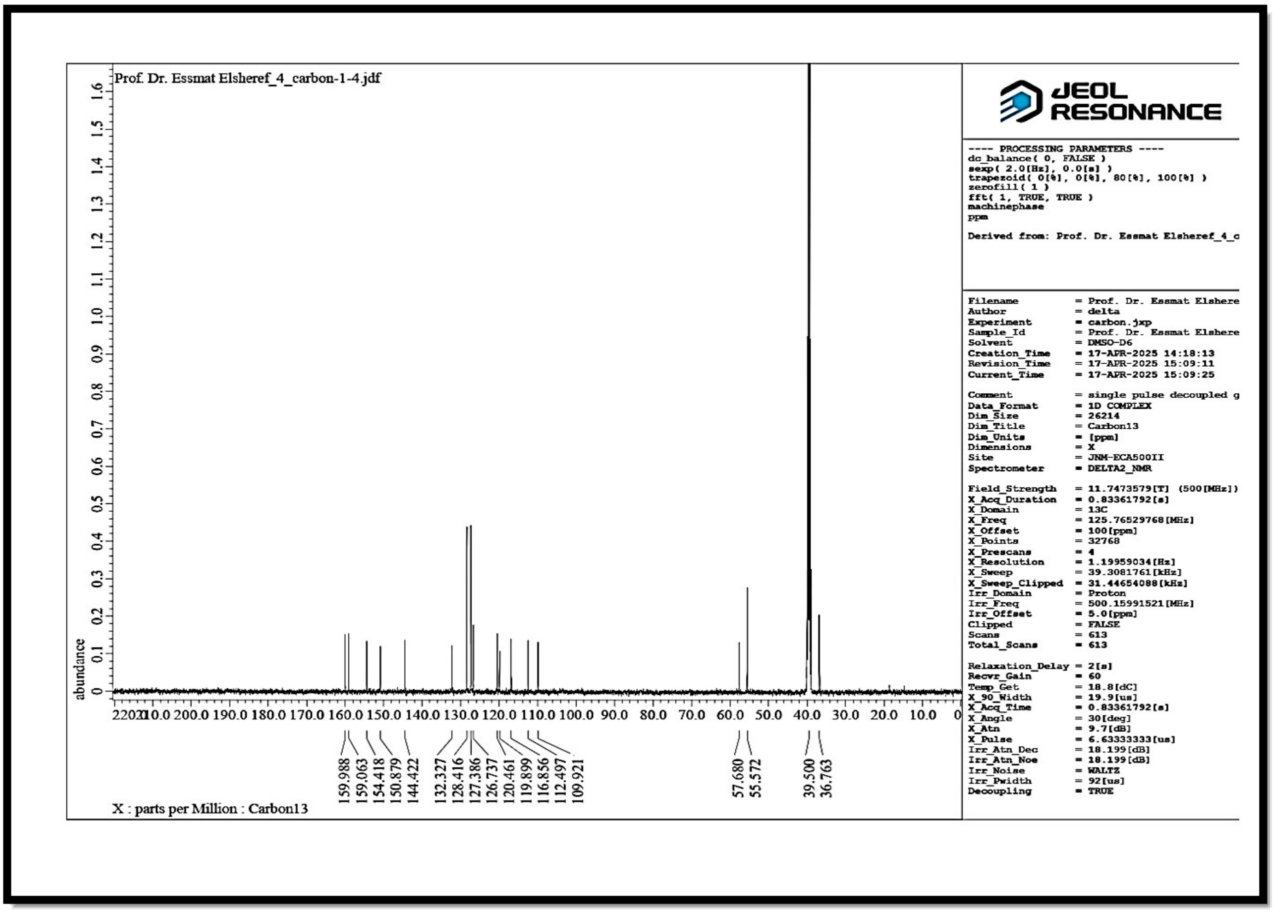

S15. ^{13}C-NMR spectrums for compound **5c**

Spectral data for compound 5d

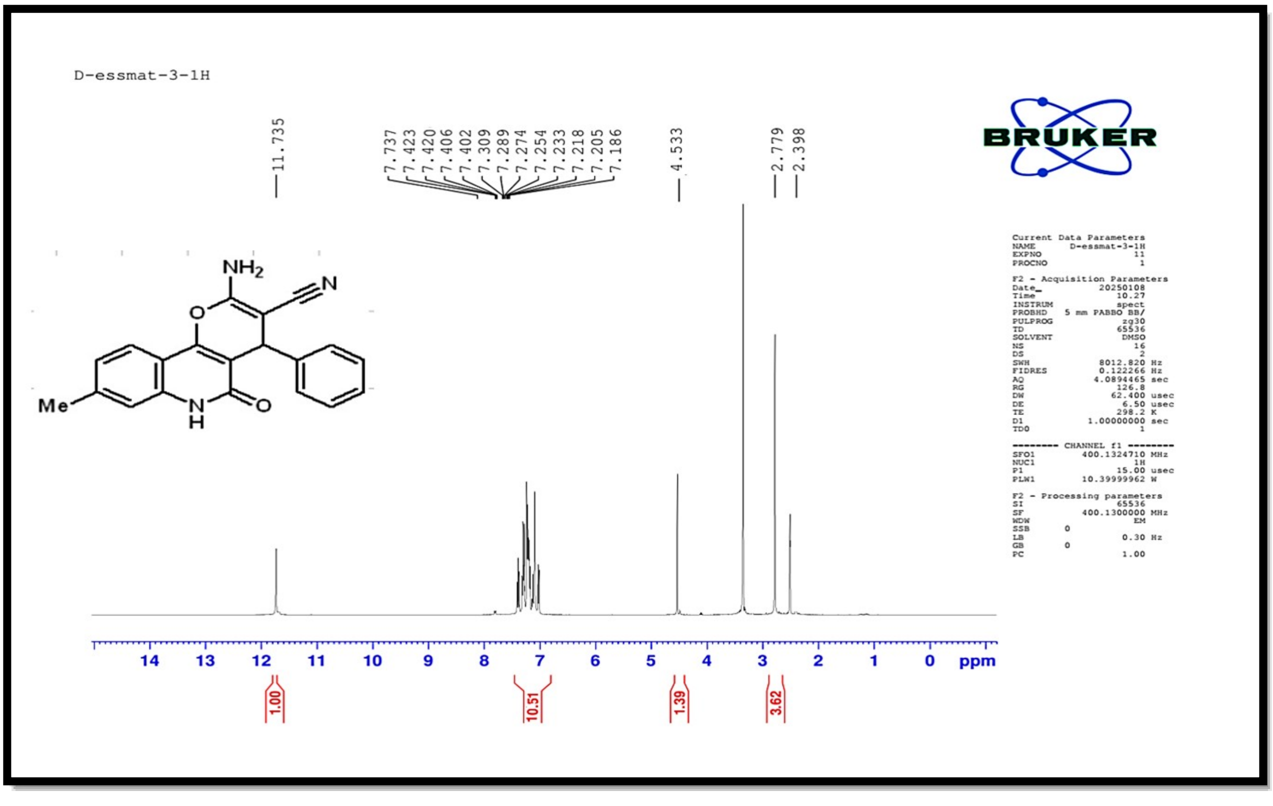

S16. ^{1}H-NMR spectrums for compound **5d**

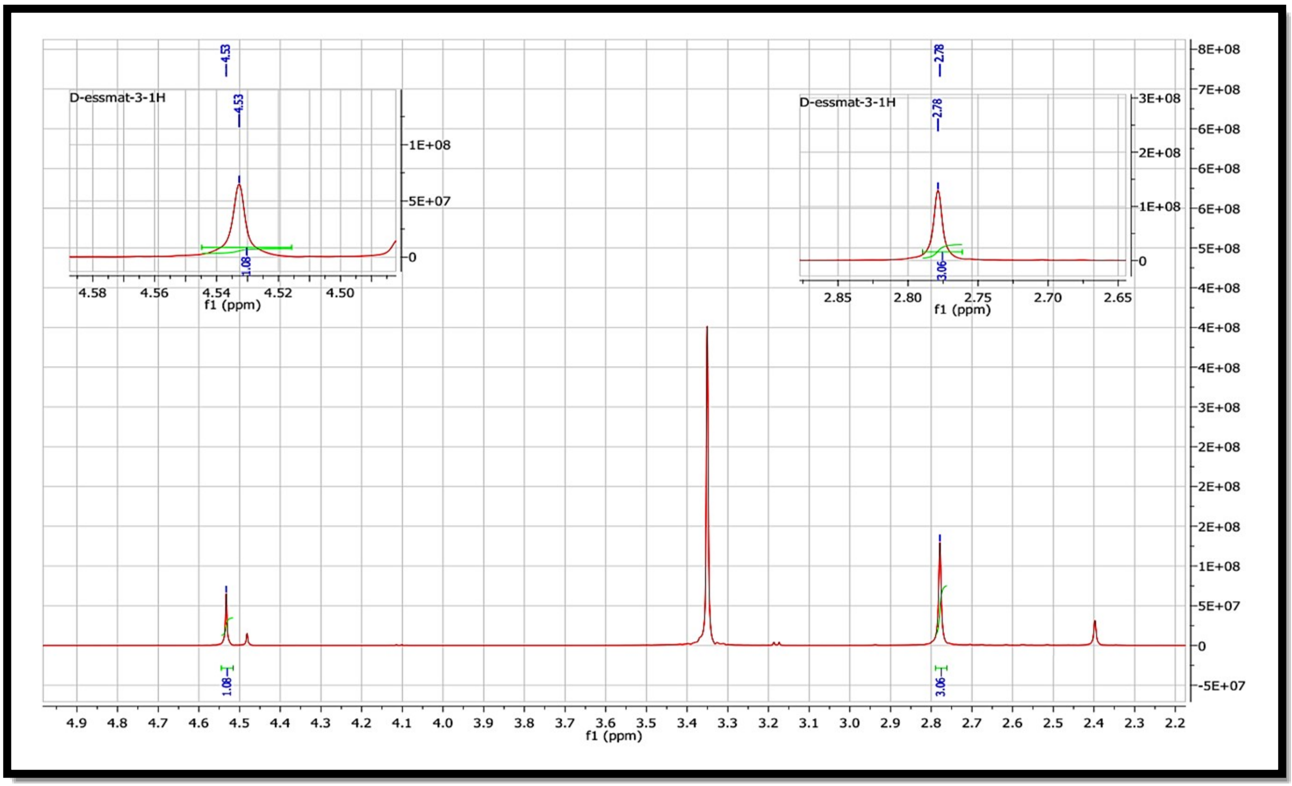

S17. Part of the ^{1}H-NMR spectrums for compound **5d**

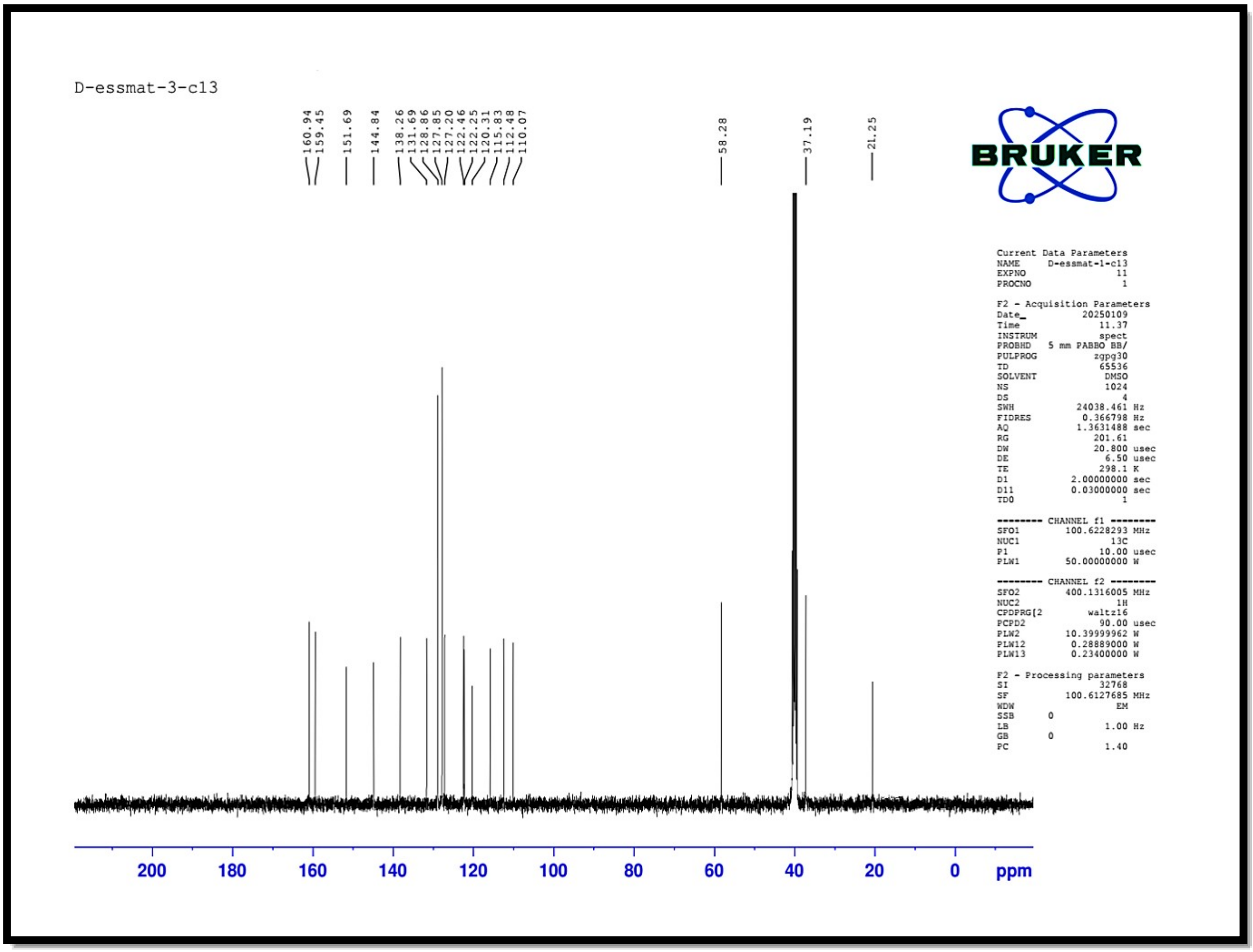

S18. ^{13}C-NMR spectrums for compound **5d**

Spectral data for compound 5e

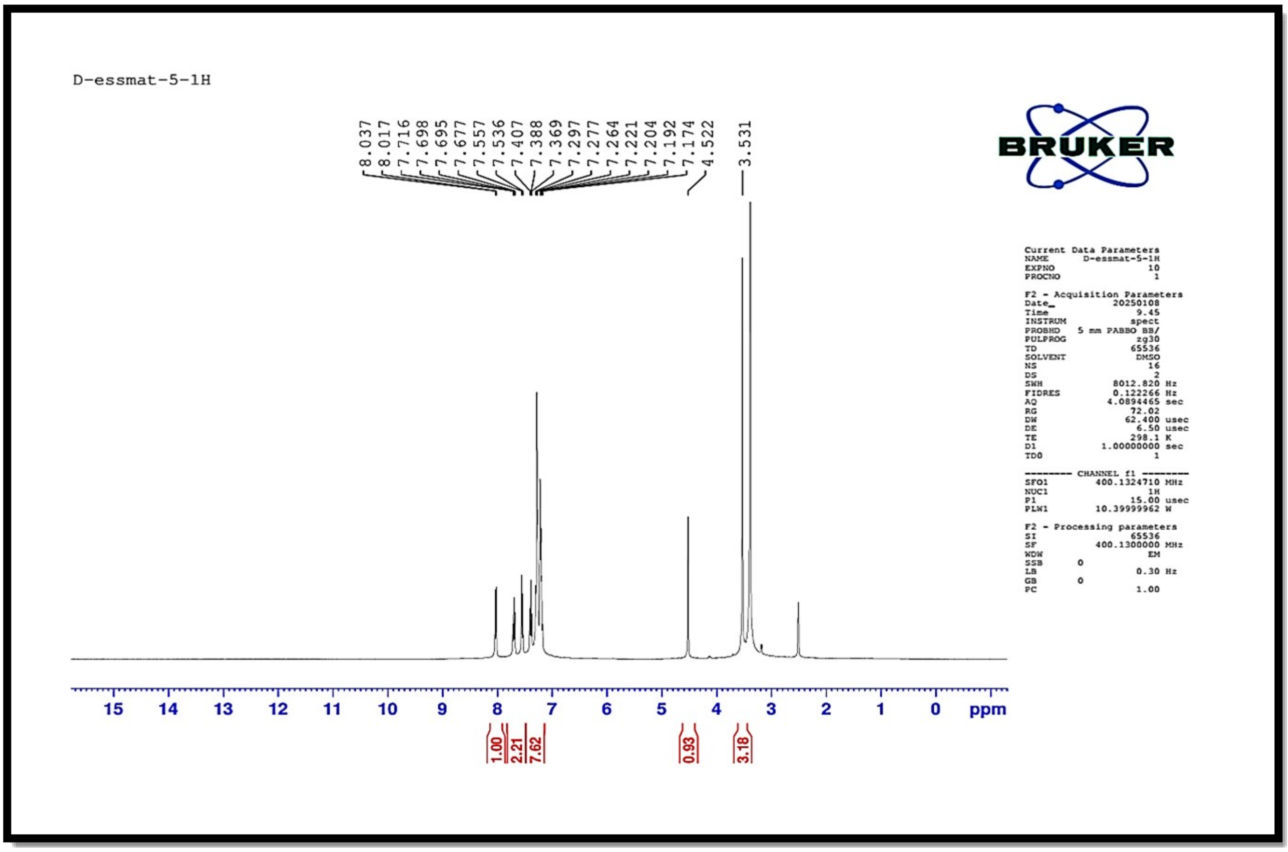

S19. ^{1}H-NMR spectrums for compound **5e**

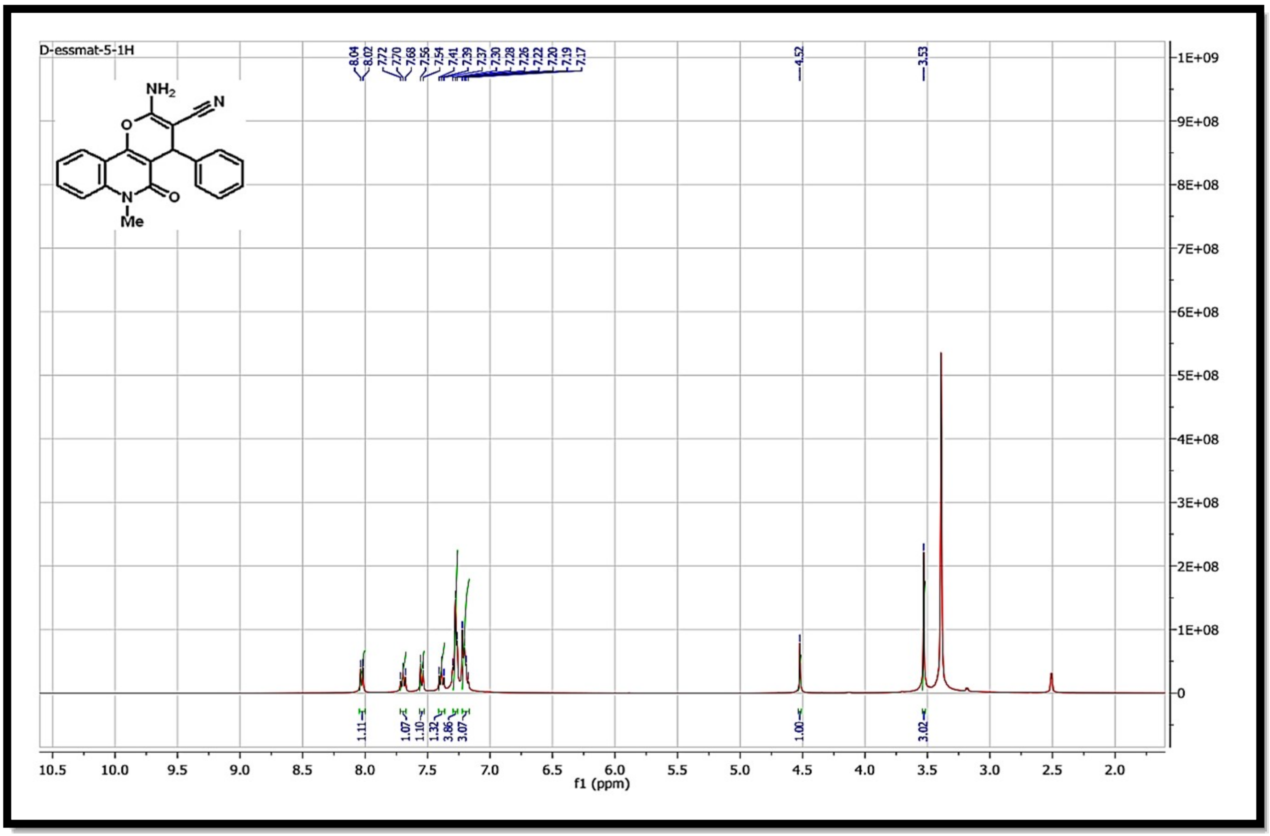

S20. Part of the ^{1}H-NMR spectrums for compound **5e**

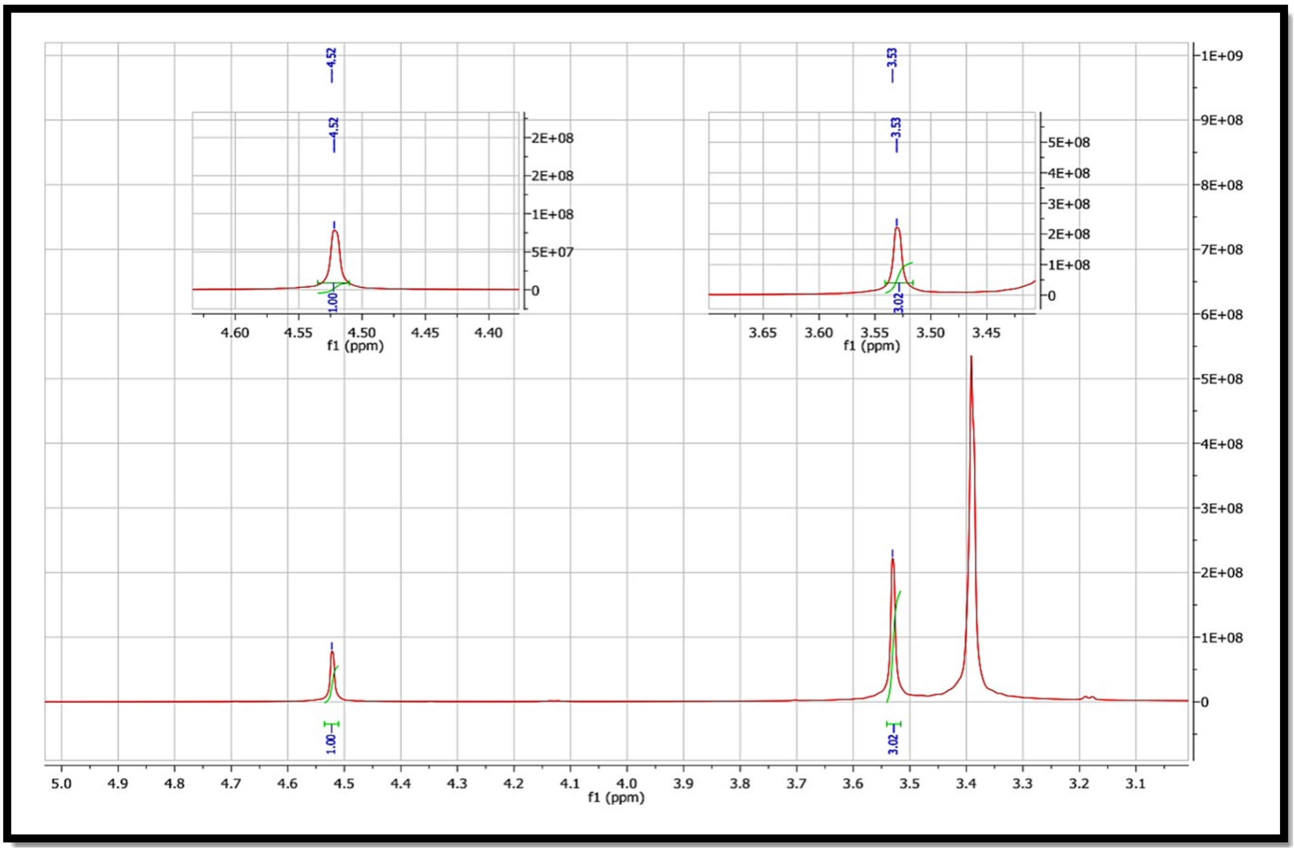

S21. Part of the ^{1}H-NMR spectrums for compound **5e**

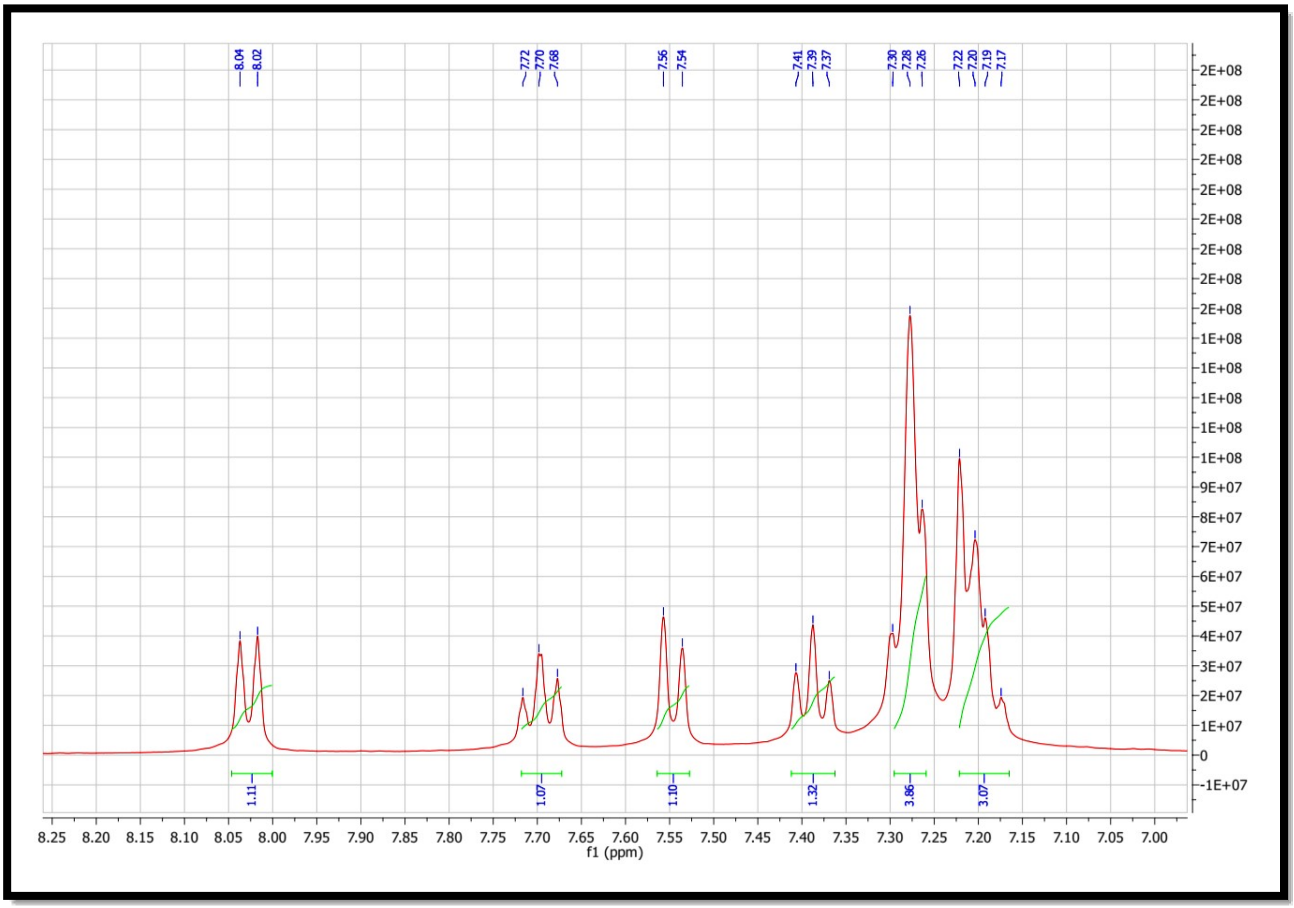

S22. Part of the ^{1}H-NMR spectrums for compound **5e**

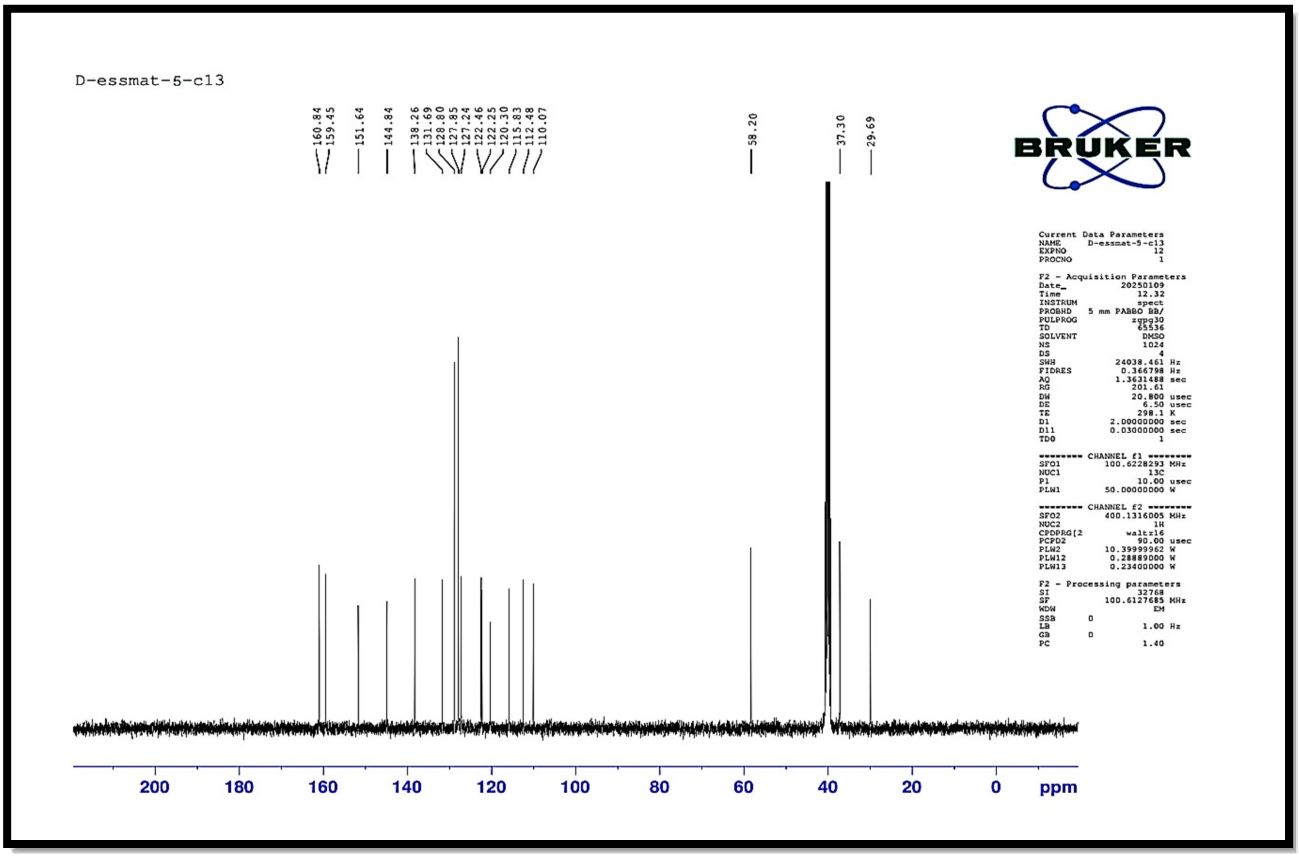

S23. ^{13}C-NMR spectrums for compound **5e**

Spectral data for compound 5f

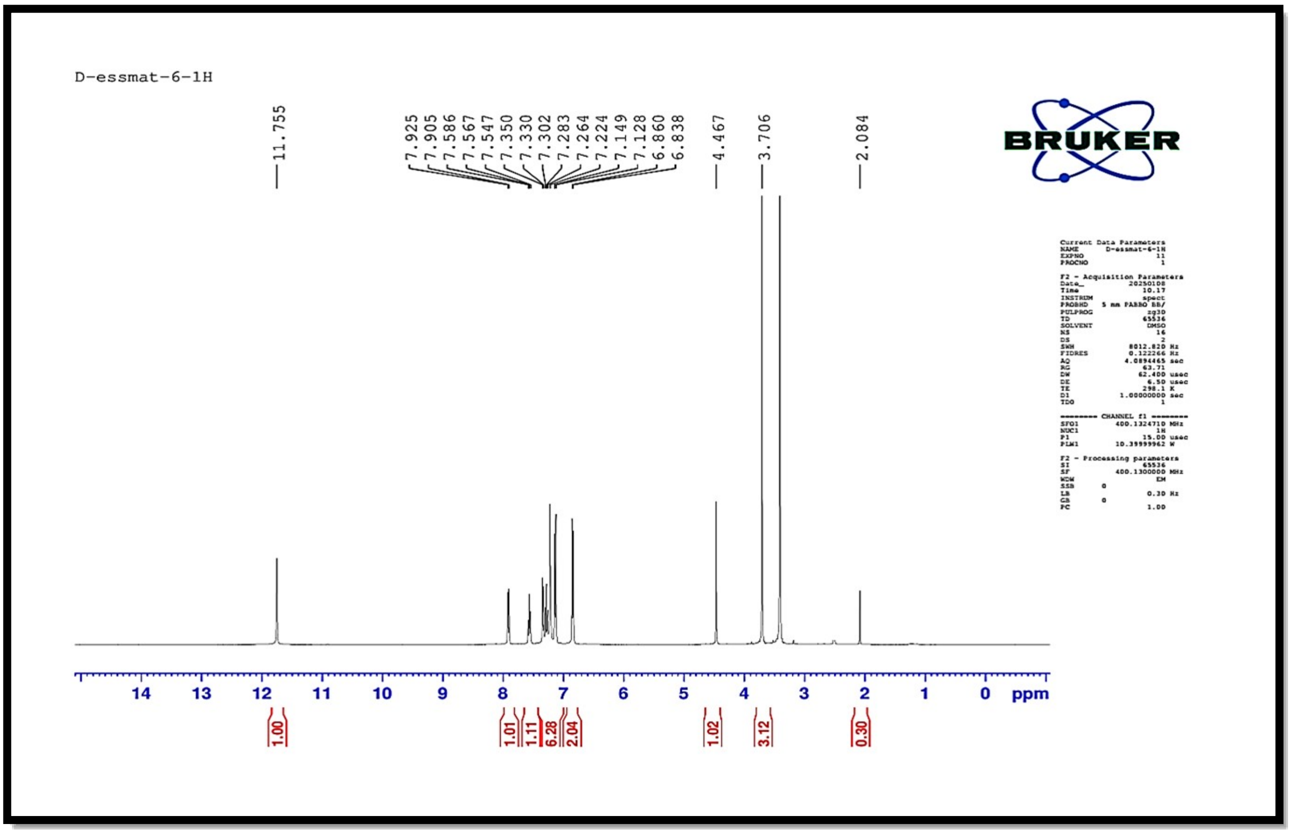

S24. ^{1}H-NMR spectrums for compound **5f**

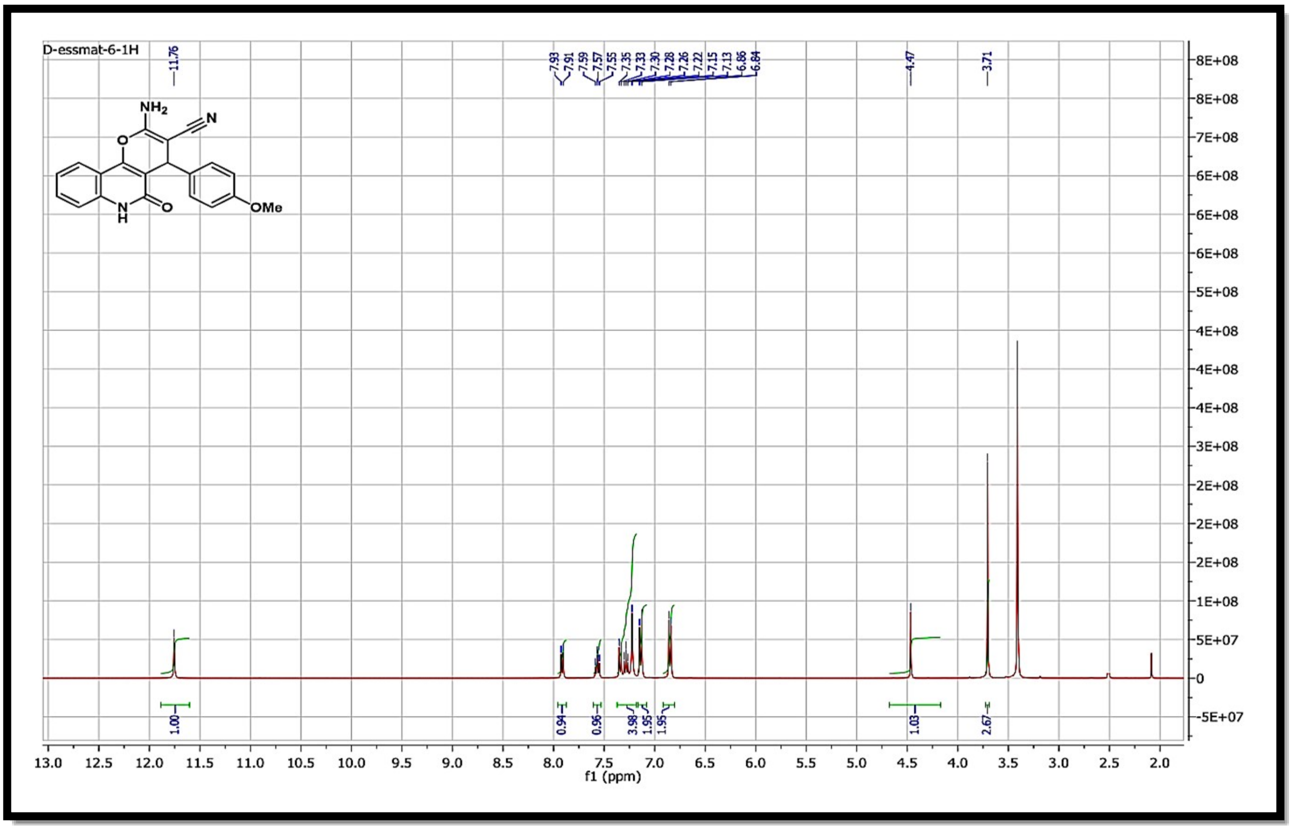

S25. Part of the ^{1}H-NMR spectrums for compound **5f**

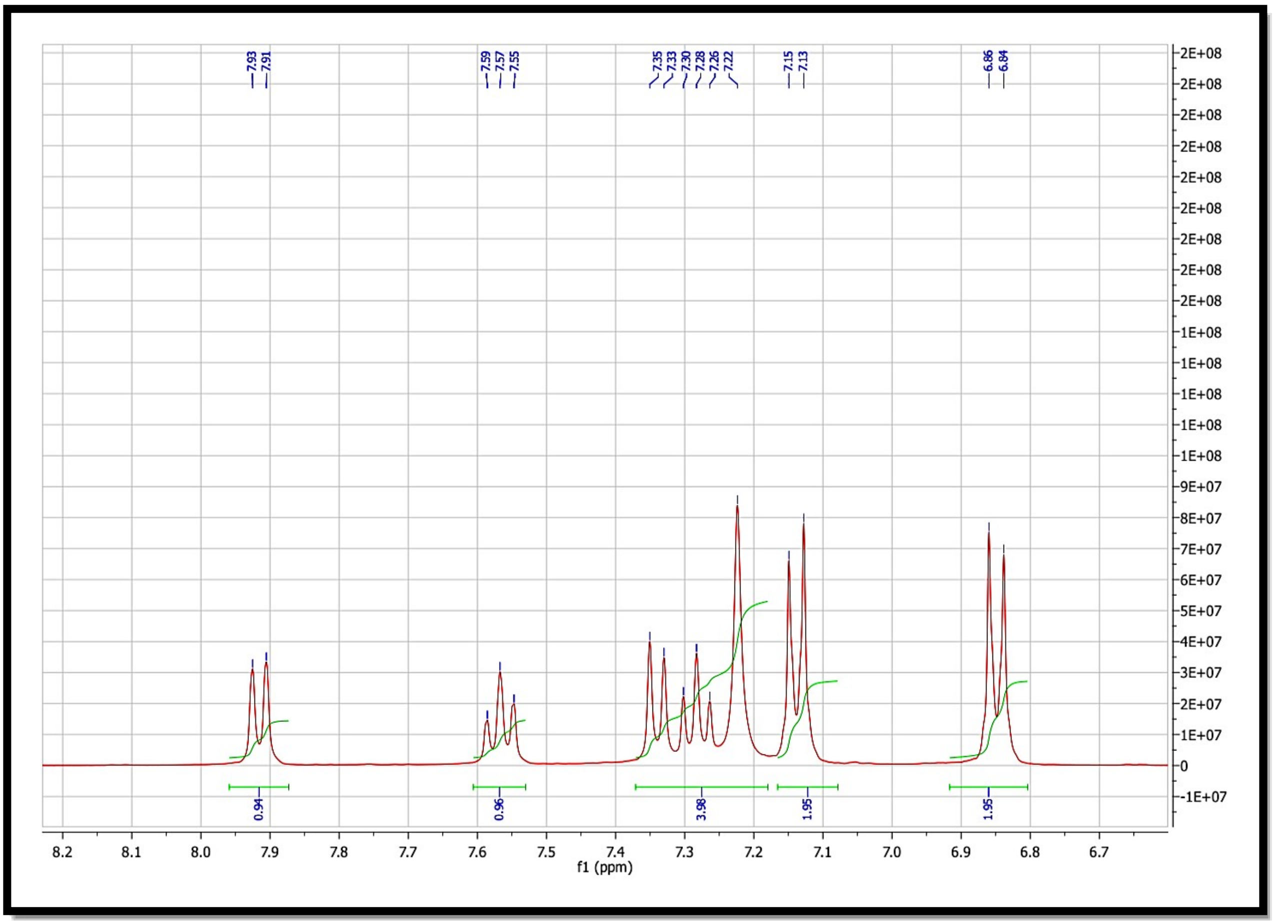

S26. Part of the ^{1}H-NMR spectrums for compound **5f**

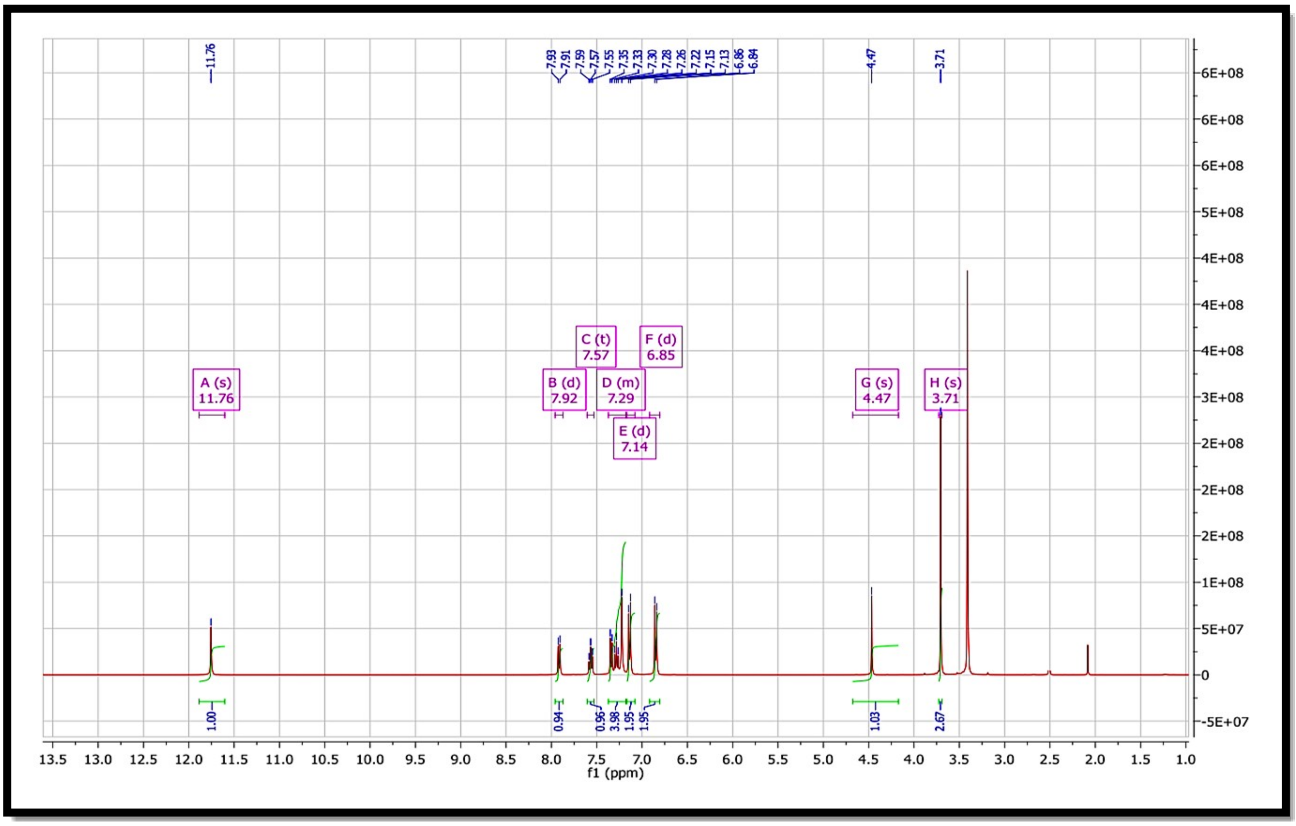

S27. Part of the ^{1}H-NMR spectrums for compound **5f**

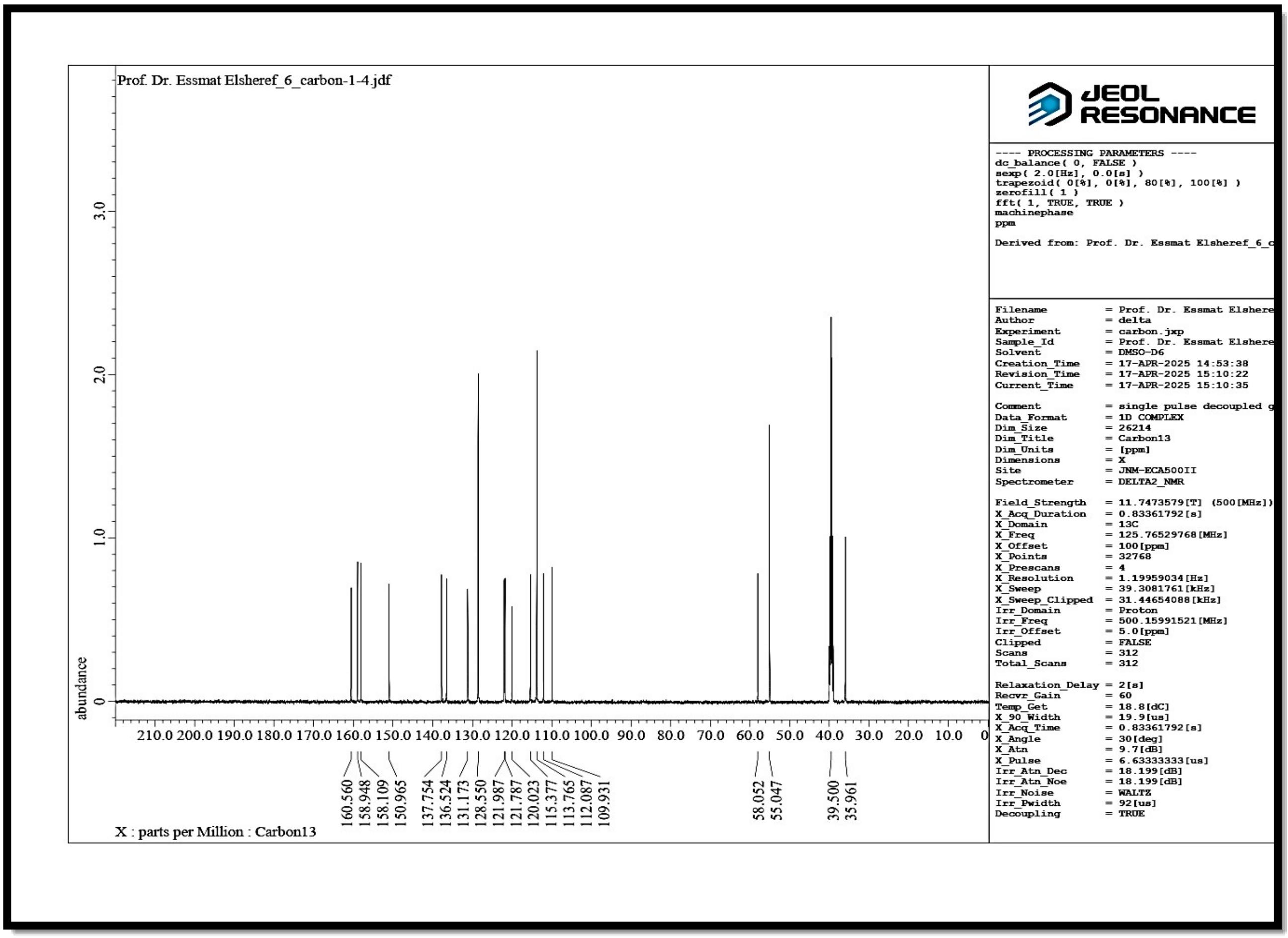

S28. ^{13}C-NMR spectrums for compound **5f**

Spectral data for compound 5g

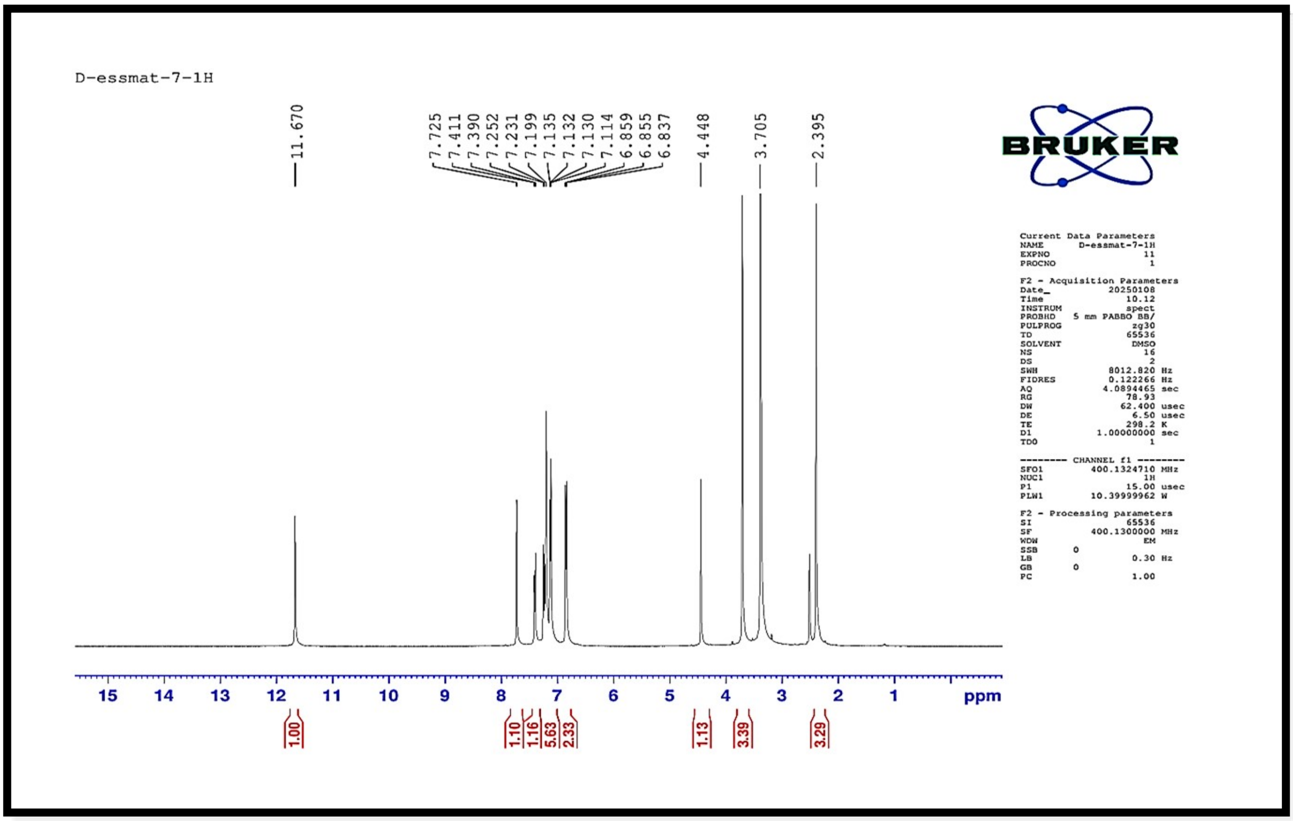

S29. 1H-NMR spectrums for compound **5g**

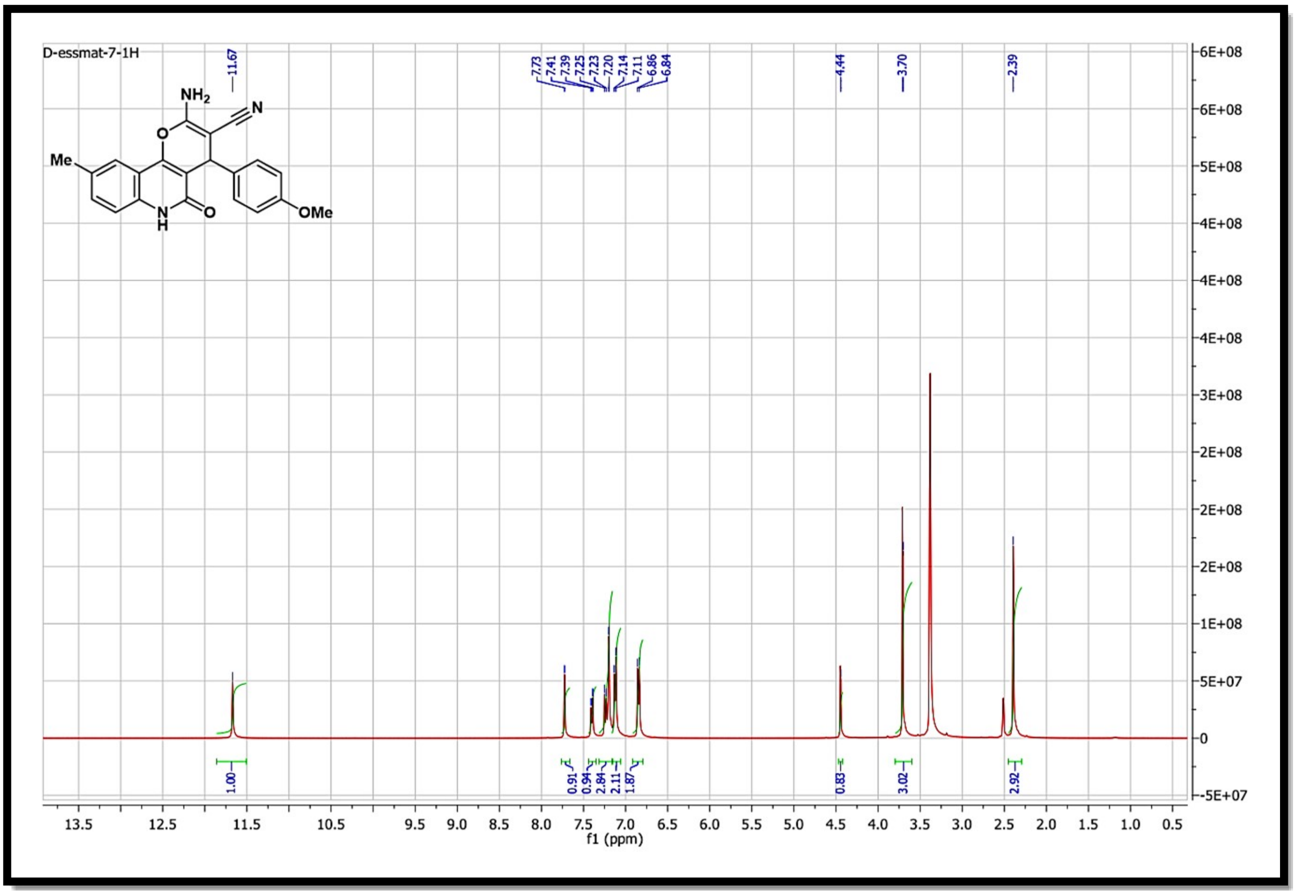

S30. Part of the 1H-NMR spectrums for compound **5g**

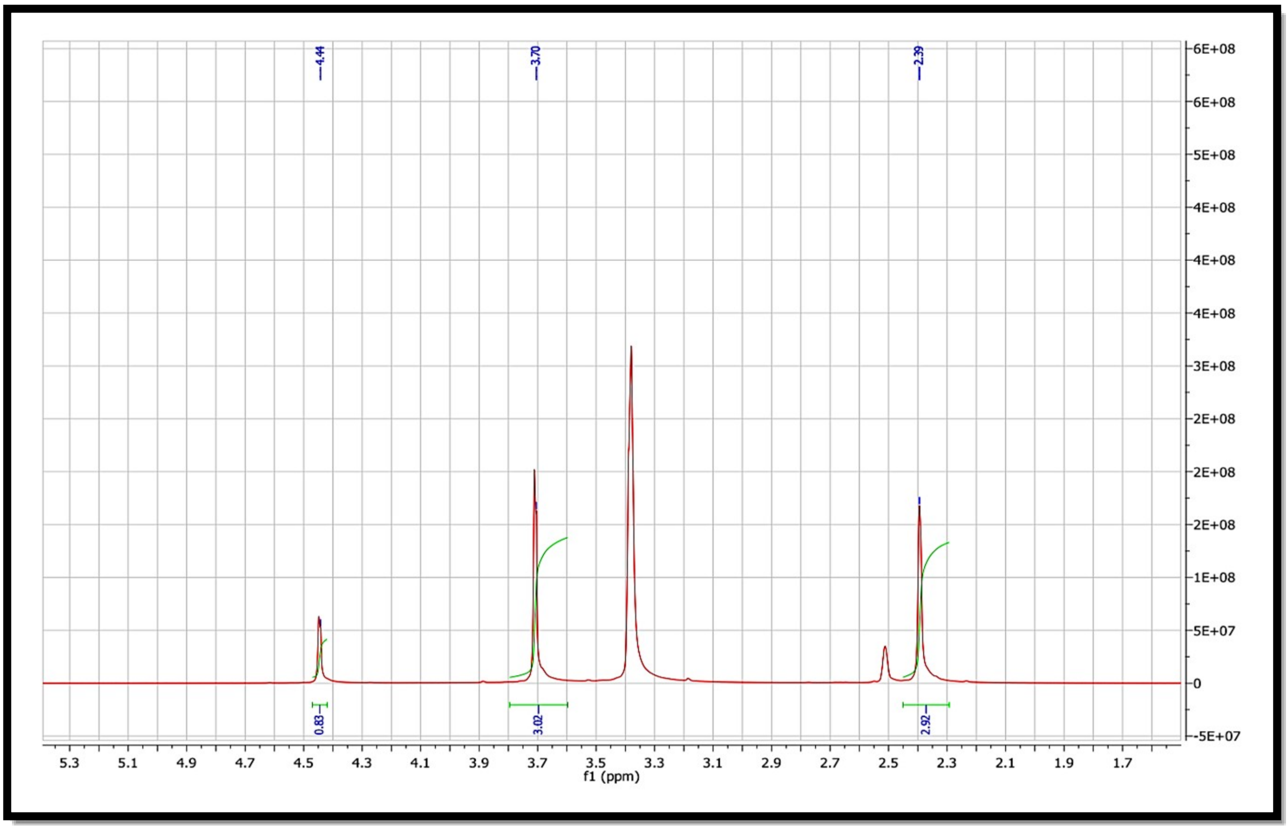

S31. Part of the ^{1}H-NMR spectrums for compound **5g**

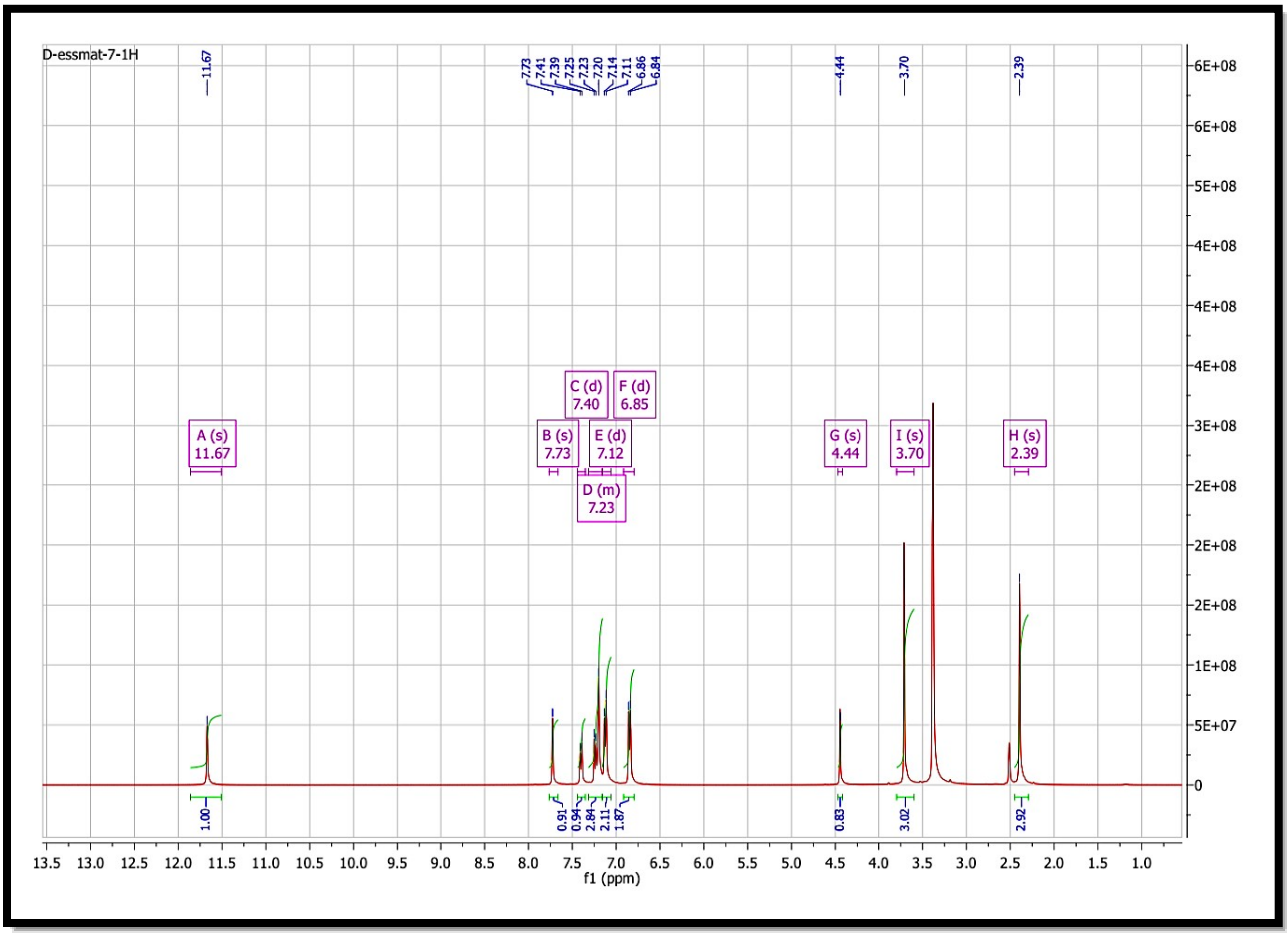

S32. Part of the ^{1}H-NMR spectrums for compound **5g**

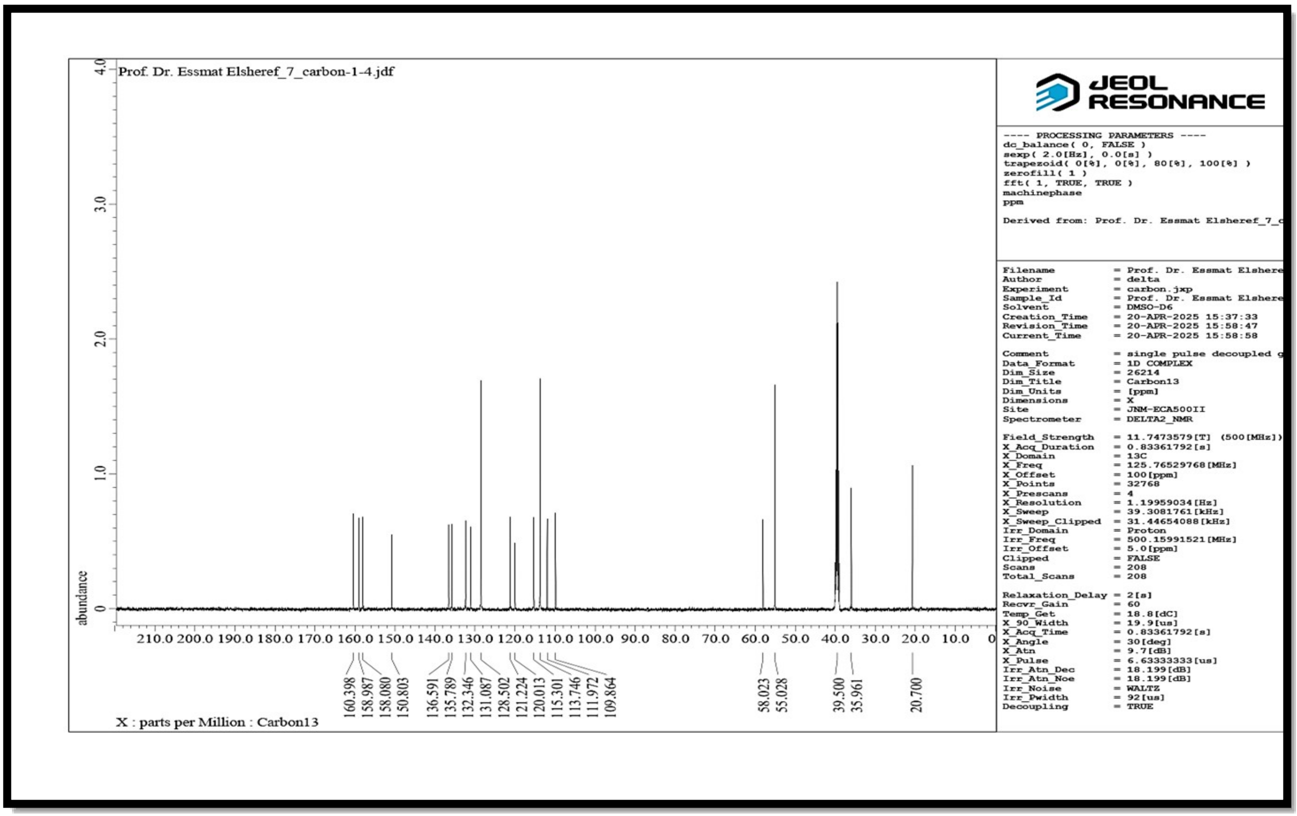

S33. ^{13}C-NMR spectrums for compound **5g**

Spectrums for compound **5h**

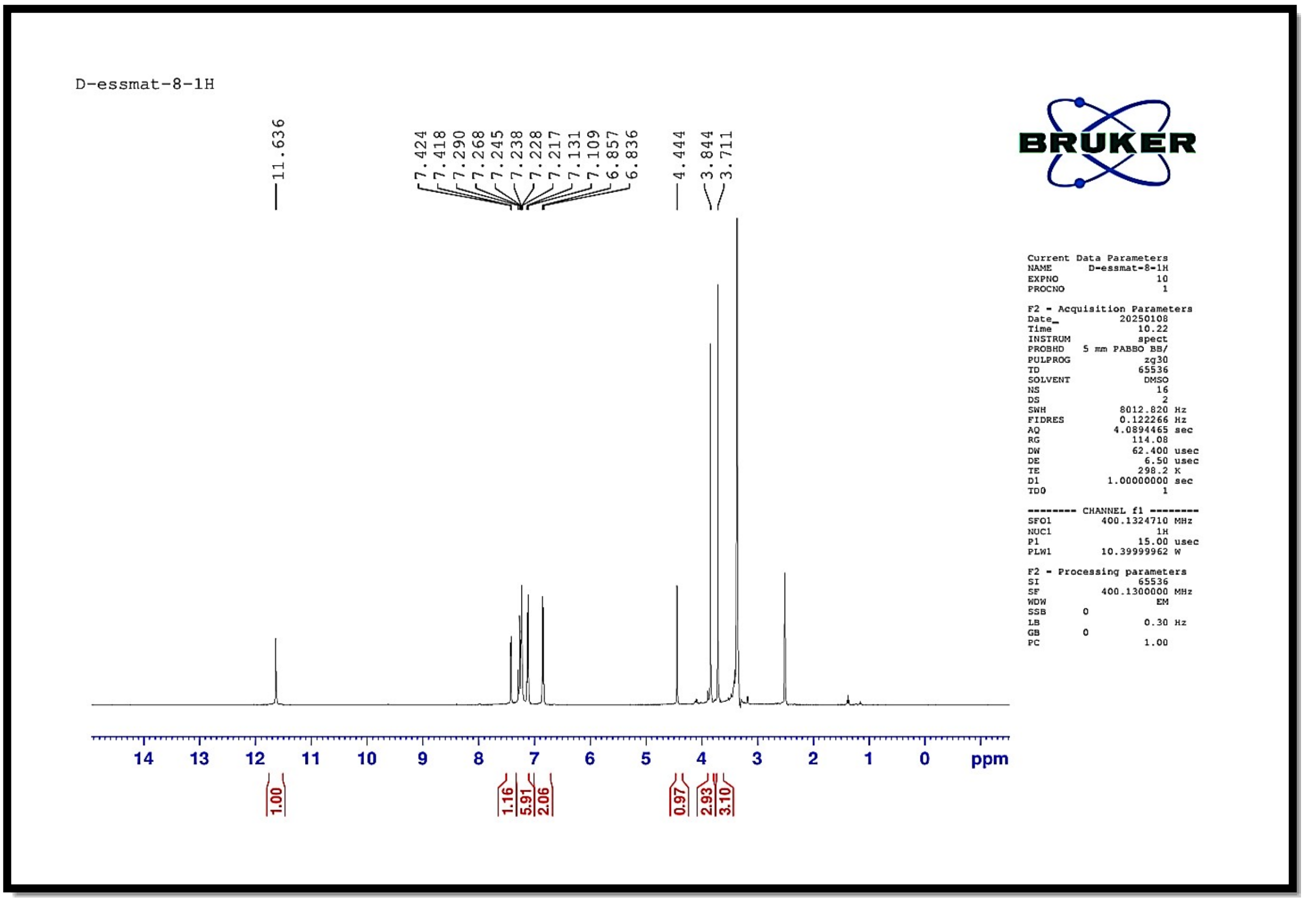

S34. ^{1}H-NMR spectrums for compound **5h**

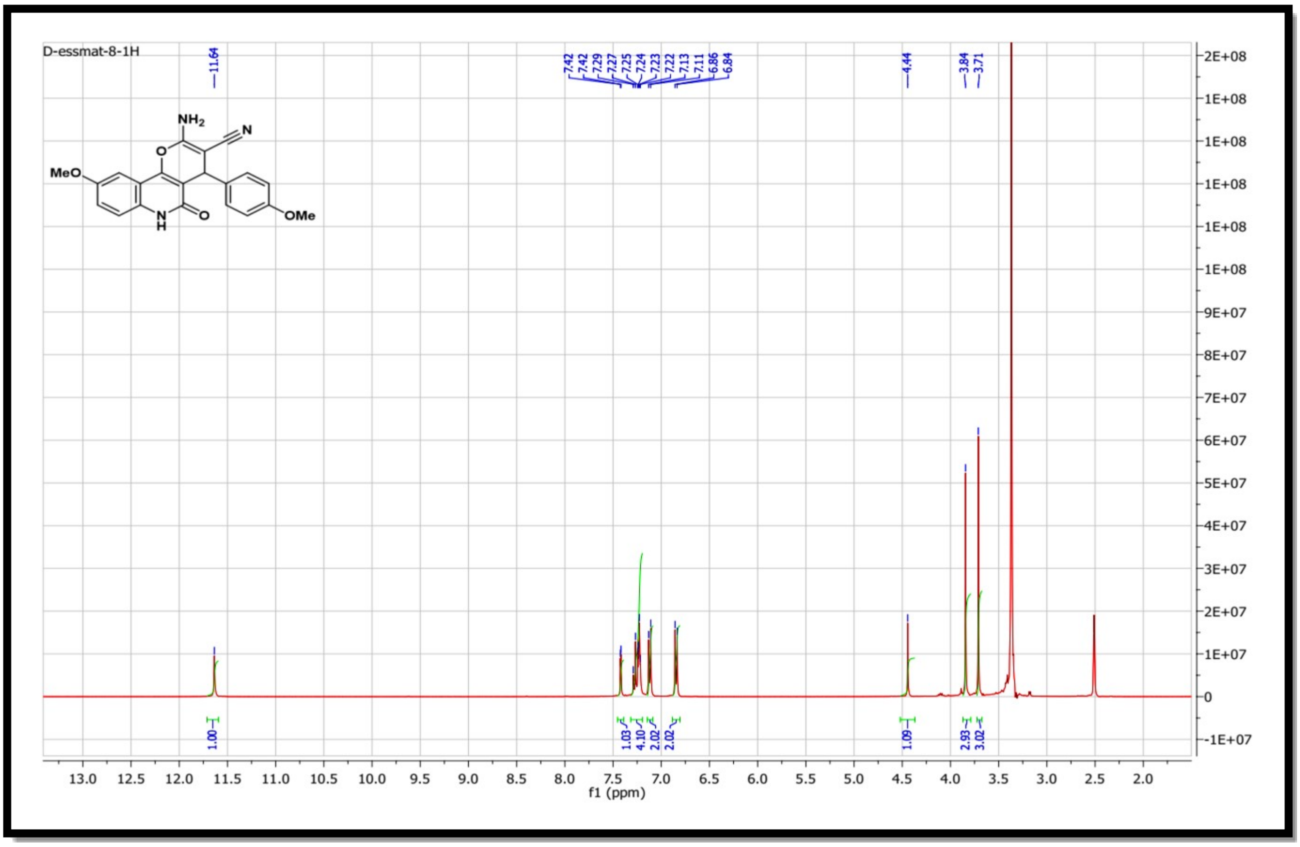

S35. Part of the ^{1}H-NMR spectrums for compound **5h**

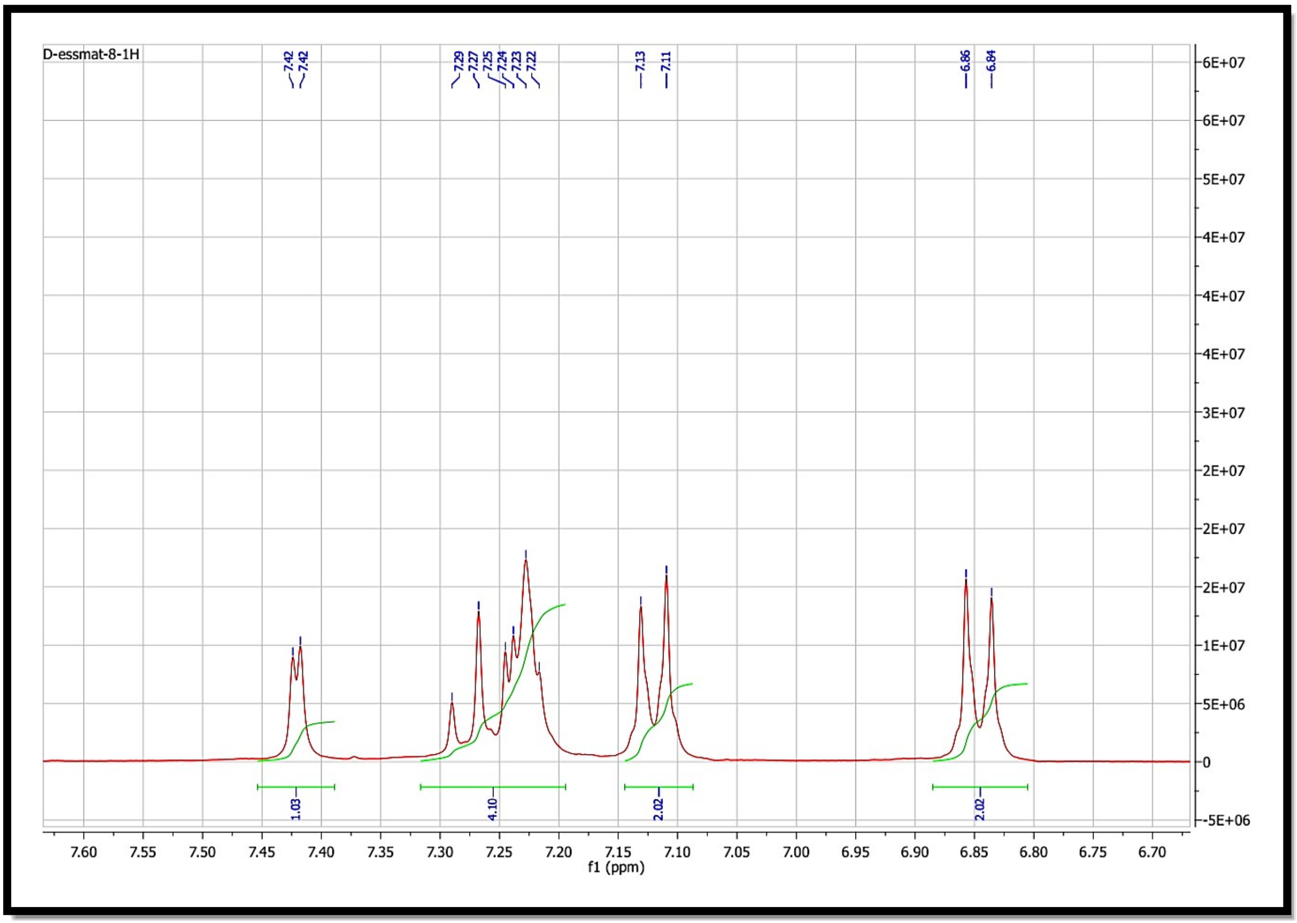

S36. Part of the ^{1}H-NMR spectrums for compound **5h**

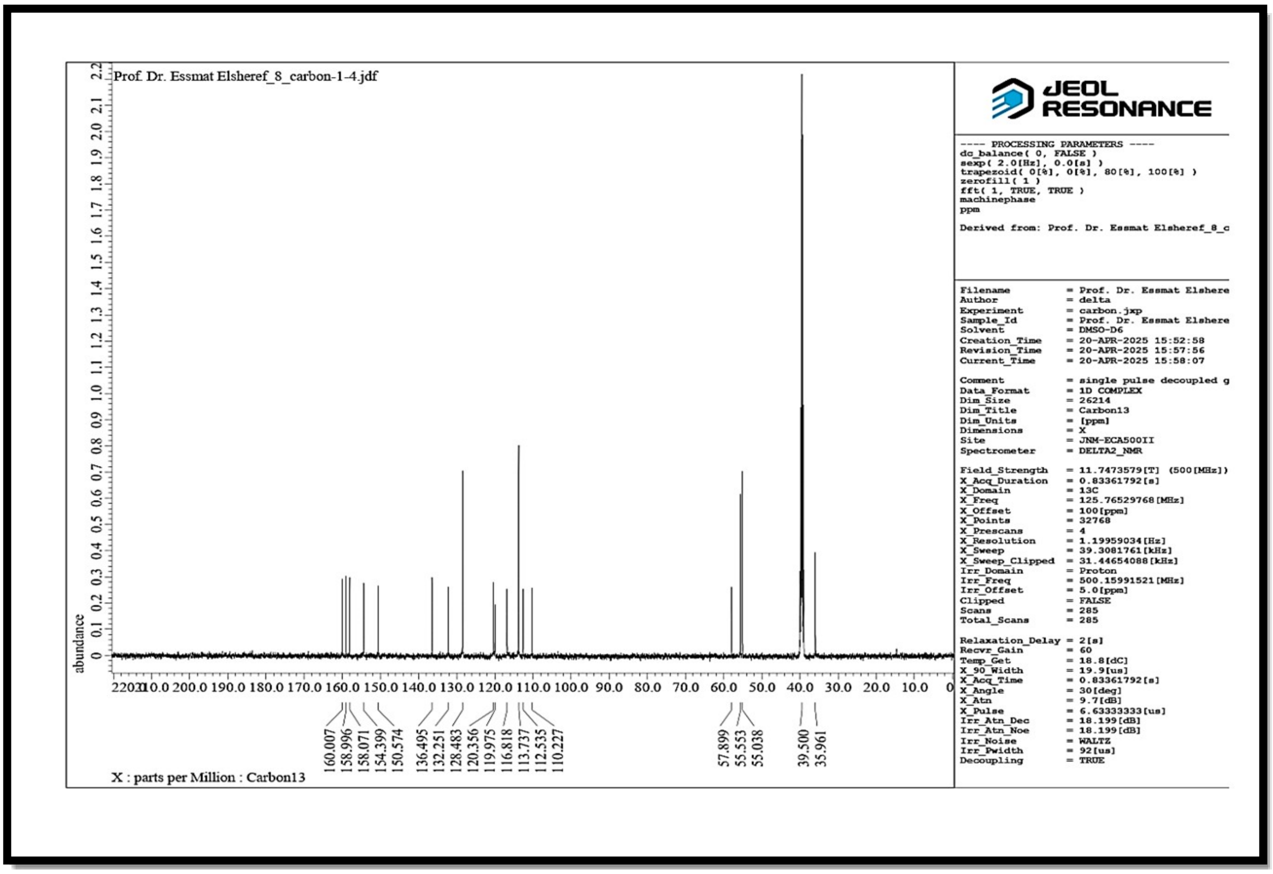

S37. ^{13}C-NMR spectrums for compound **5h**

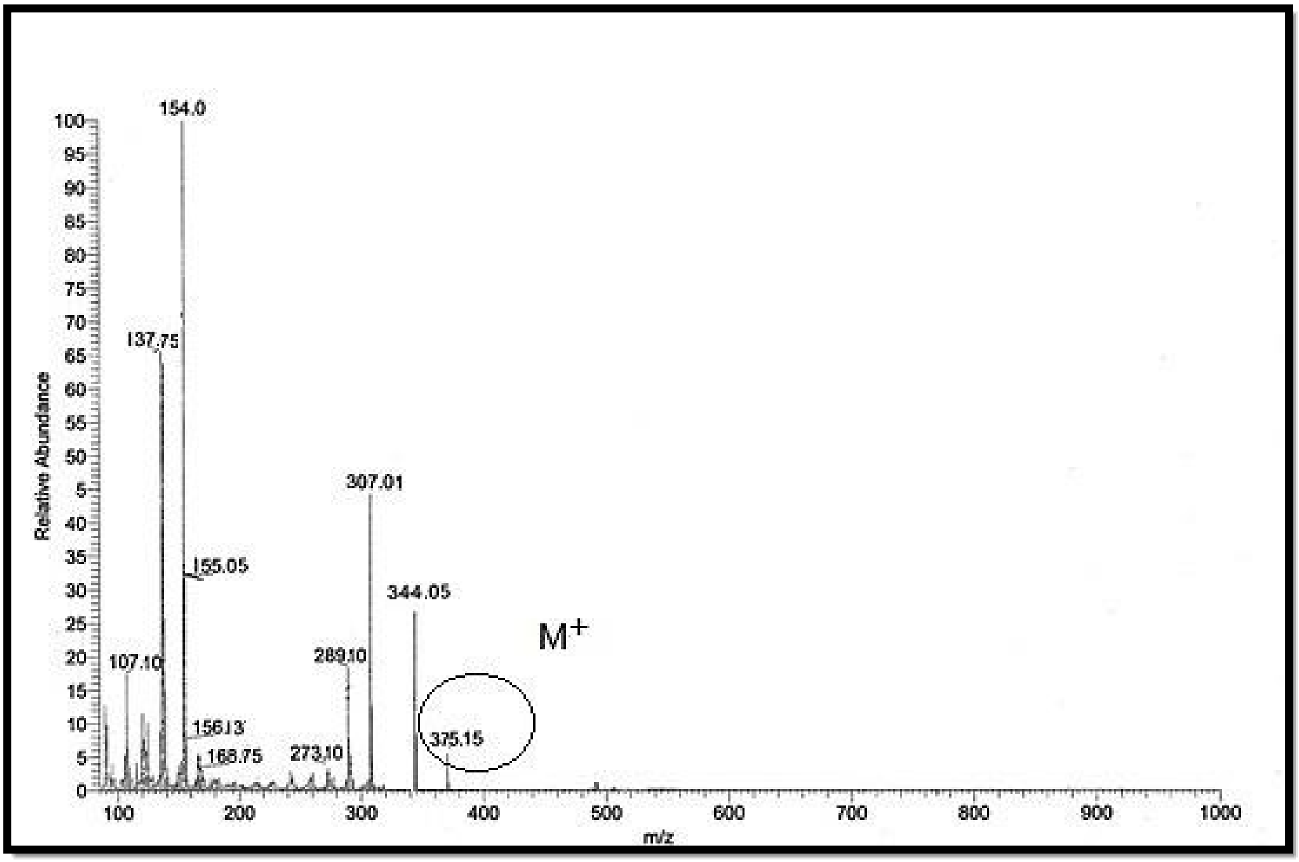

S38. Mass spectrometry for compound **5h**

Spectrums for compound **5i**

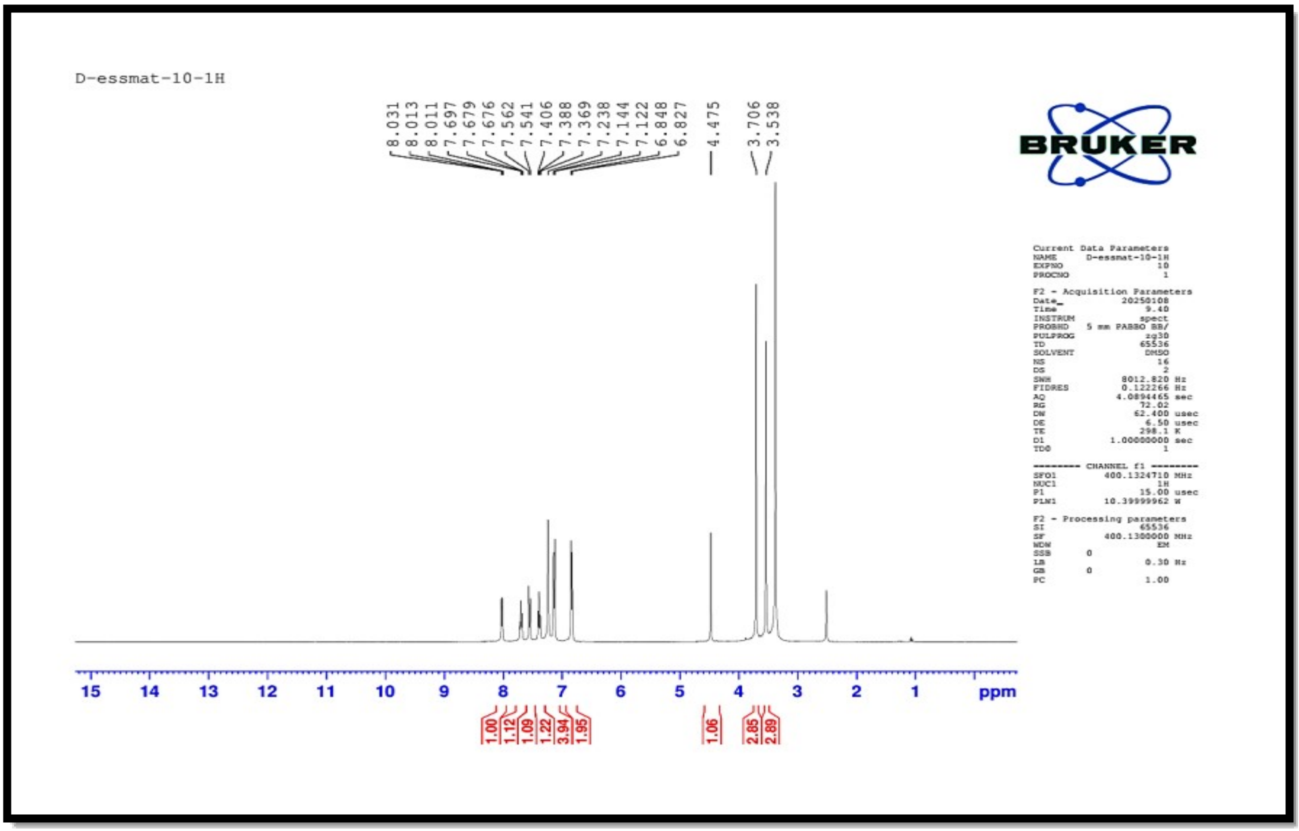

S39. ^{1}H-NMR spectrums for compound **5i**

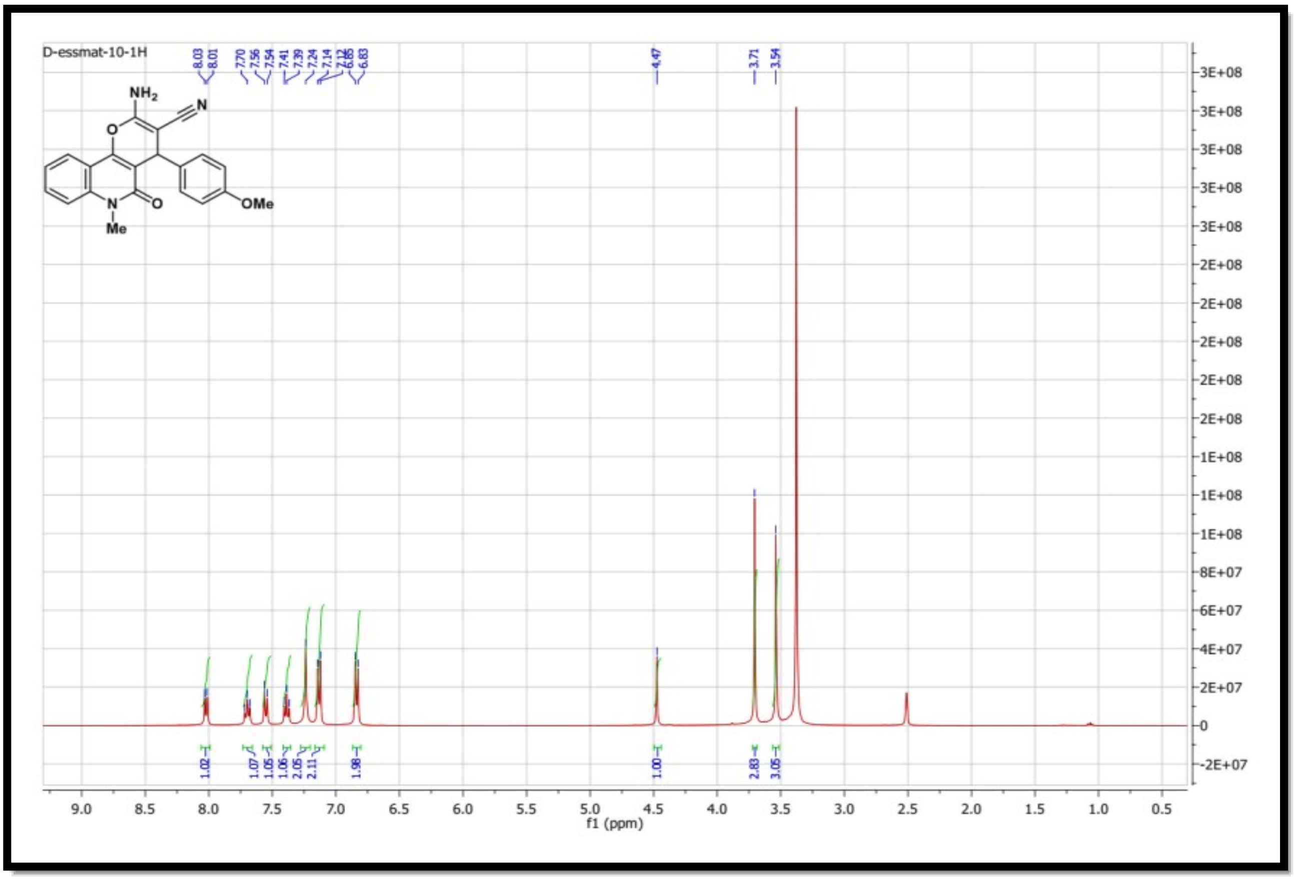

S40. ^{1}H-NMR spectrums for compound **5i**

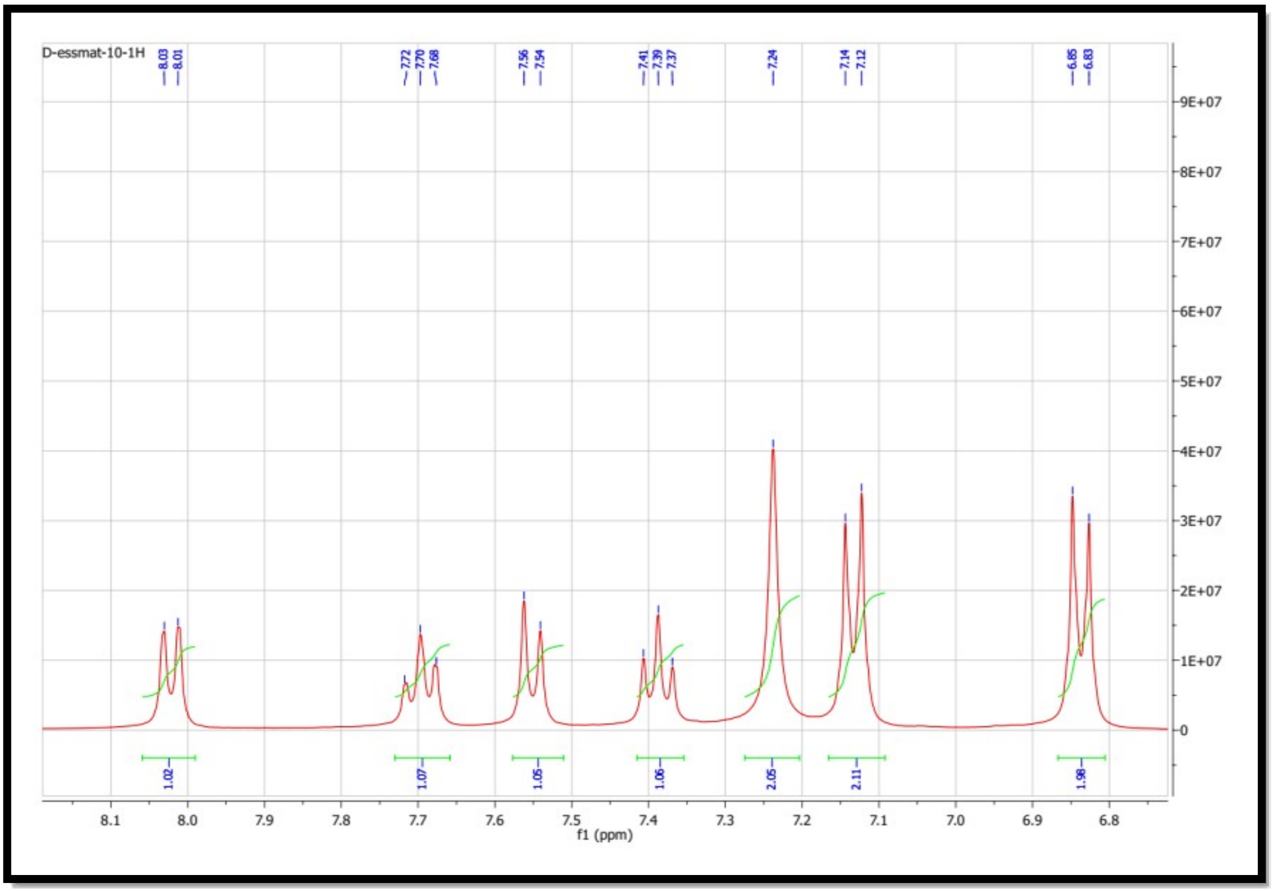

S41. Part of the ^{1}H-NMR spectrums for compound **5i**

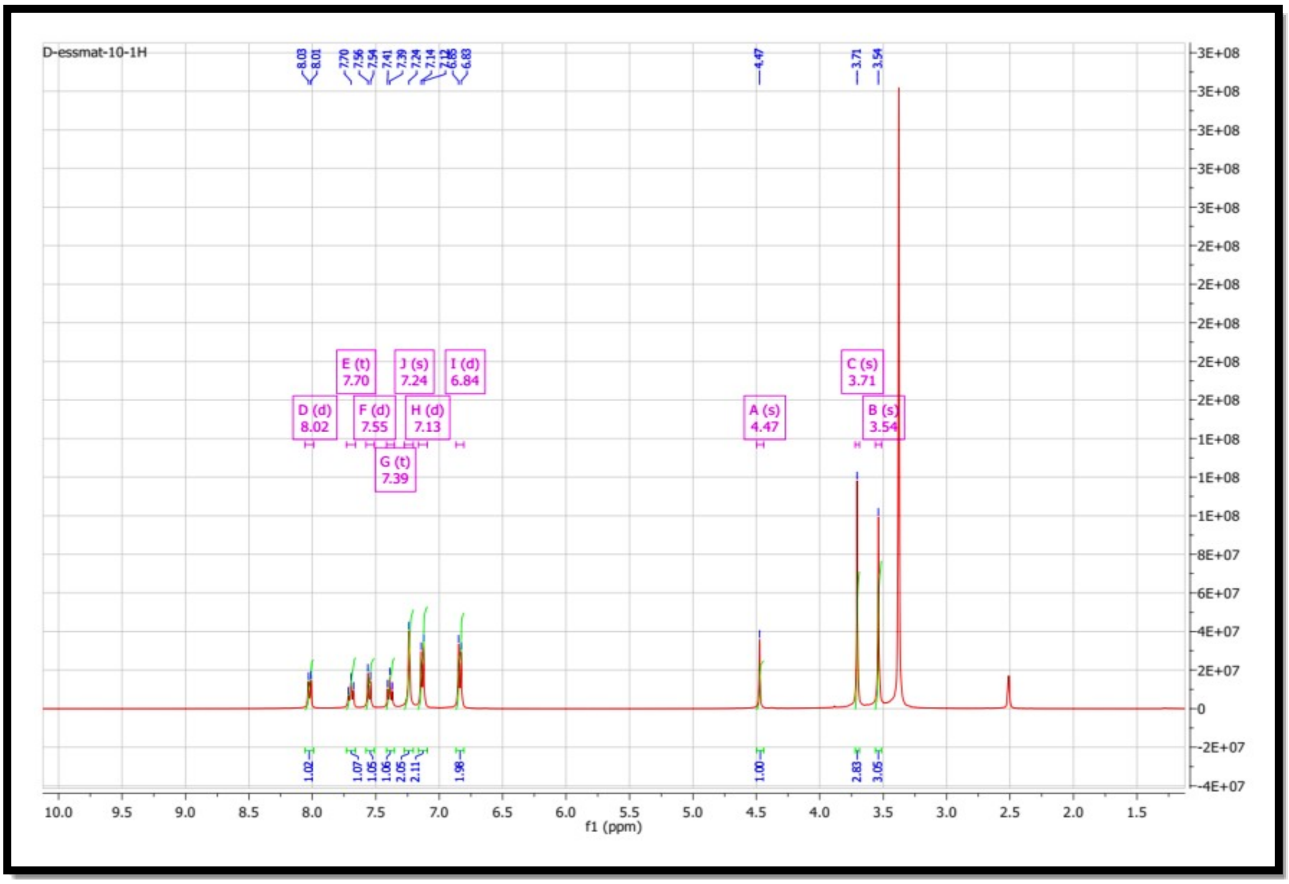

S42. Part of the ^{1}H-NMR spectrums for compound **5i**

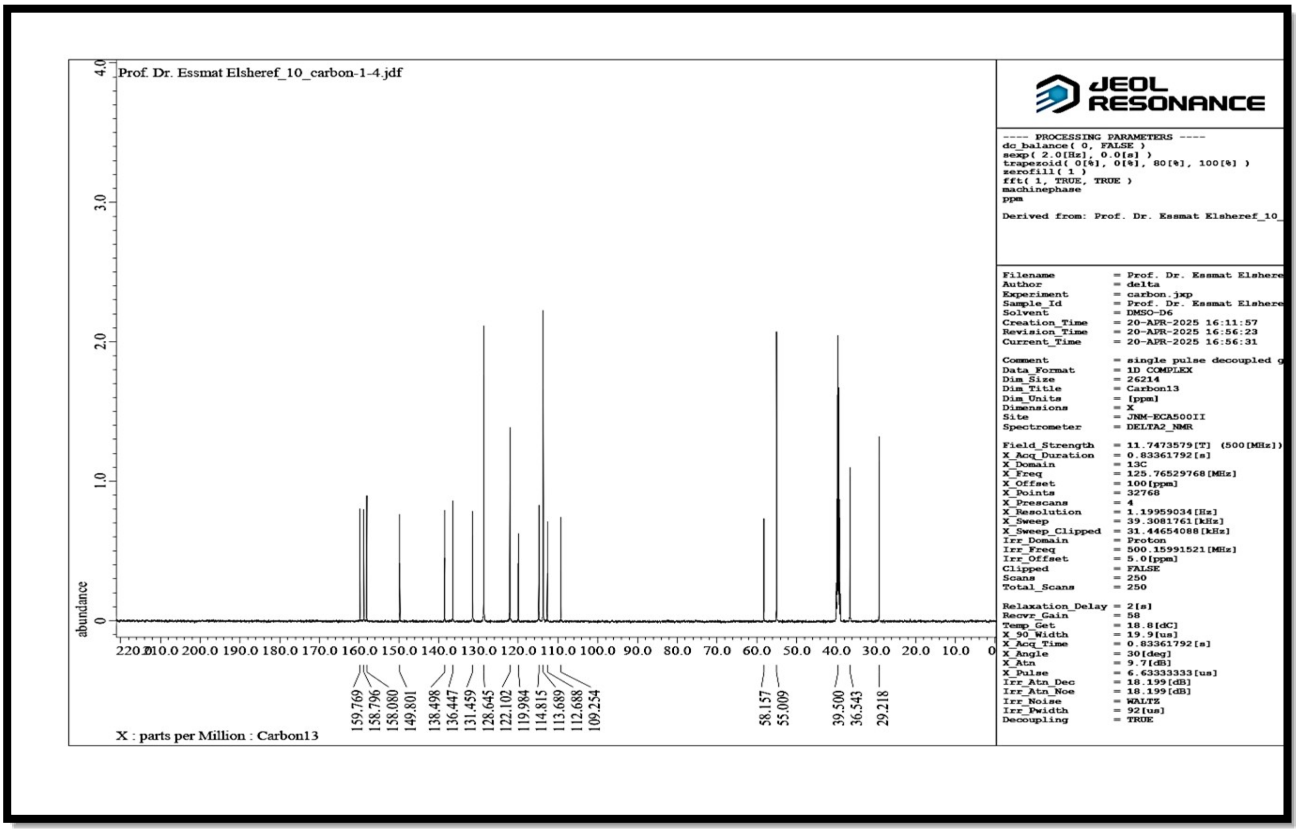

S43. ^{1}H-NMR spectrums for compound **5i**

Spectrums for compound **5j**

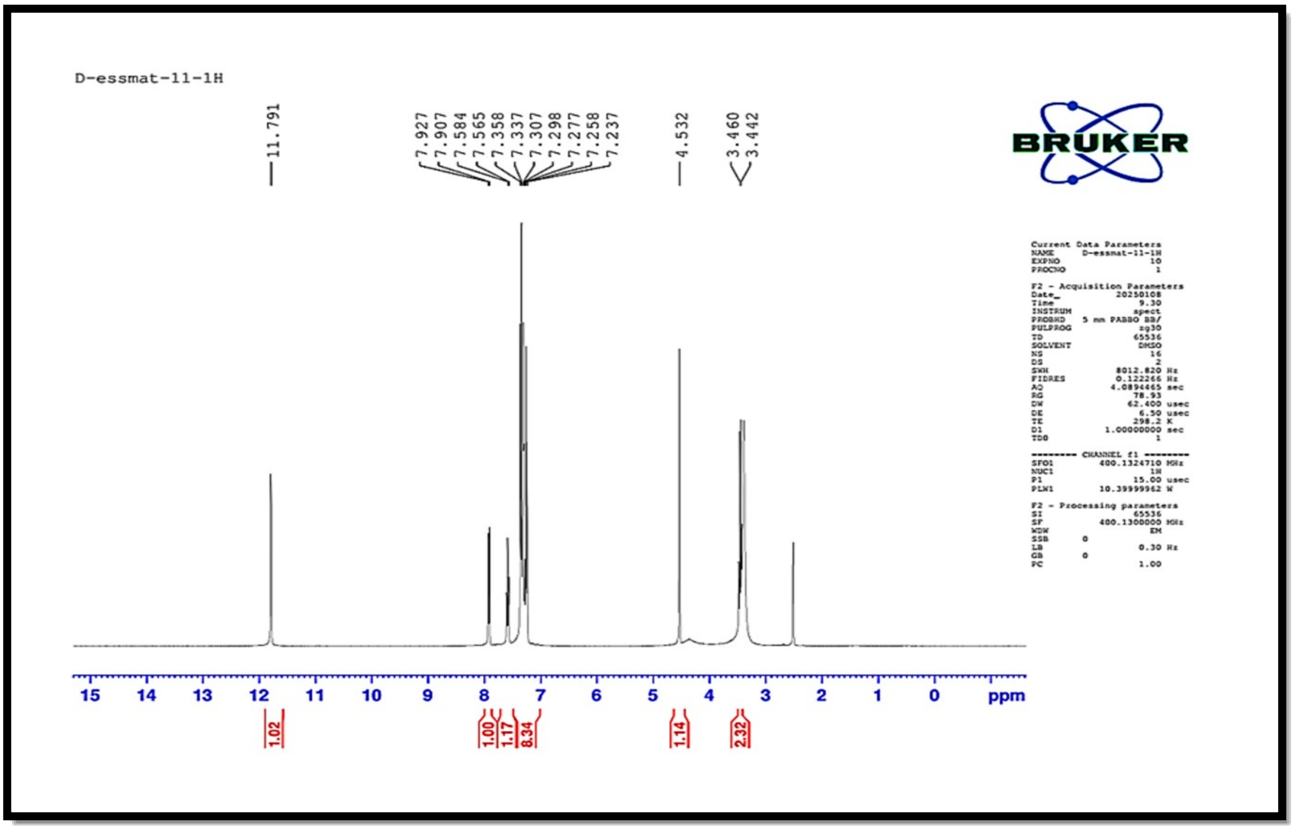

S44. ^{1}H-NMR spectrums for compound **5j**

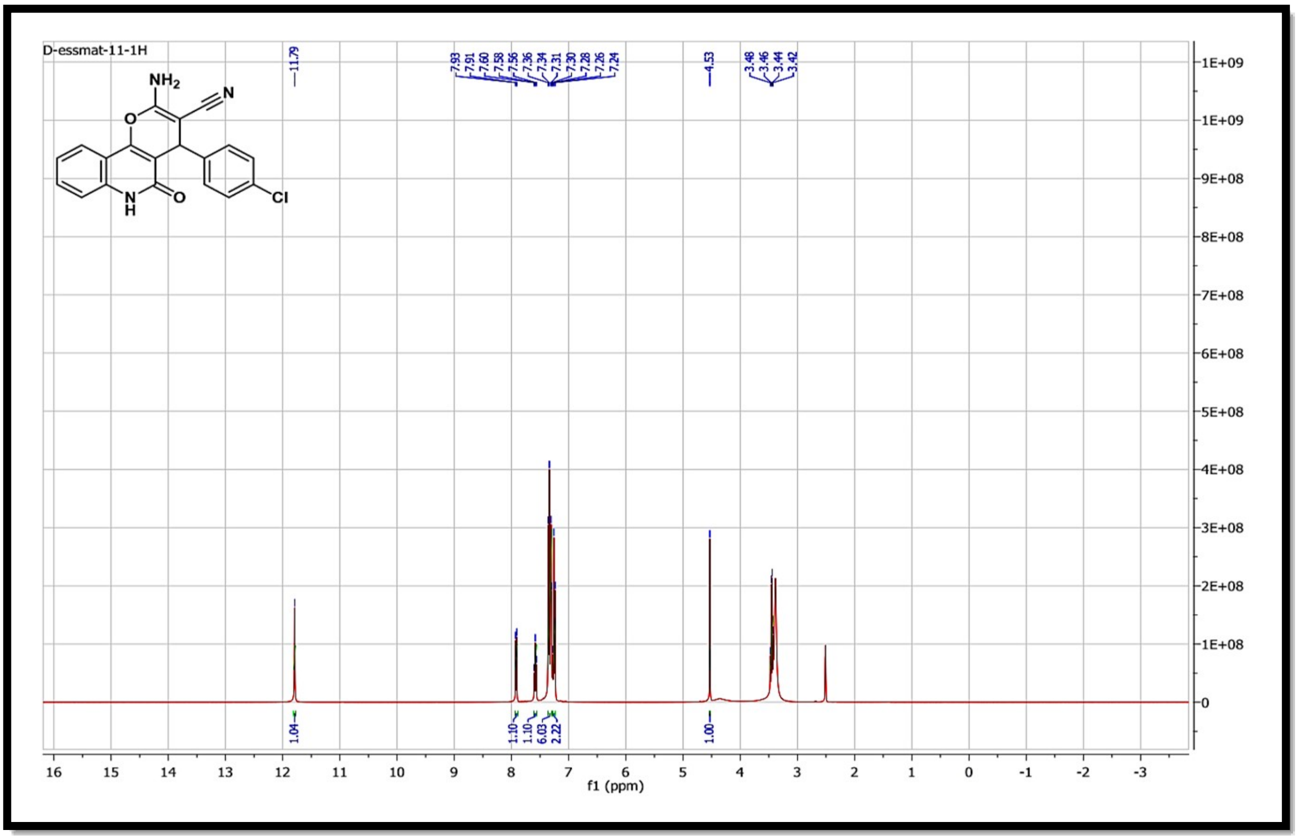

S45. ^{1}H-NMR spectrums for compound **5j**

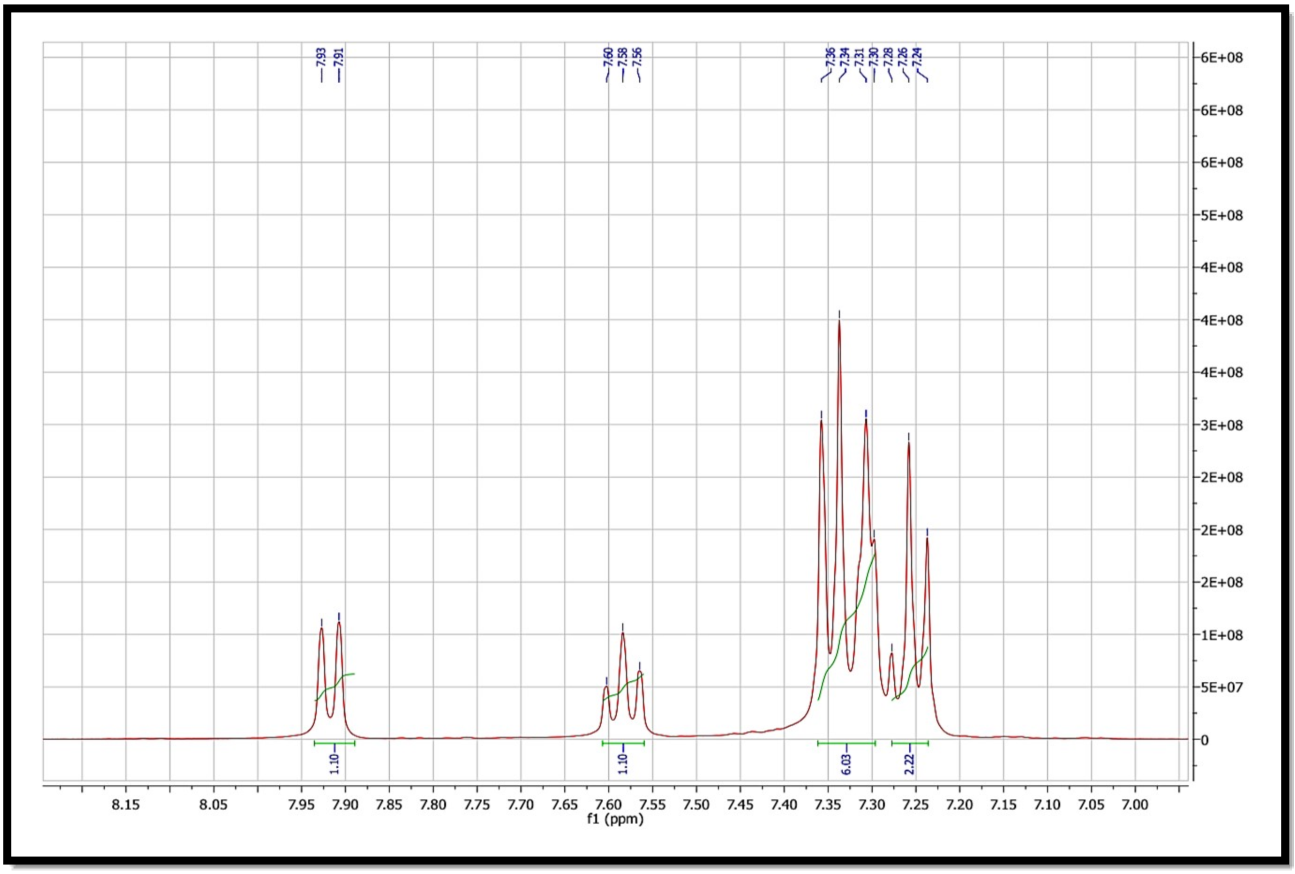

S46. Part of the ^{1}H-NMR spectrums for compound **5j**

Spectrums for compound **5k**

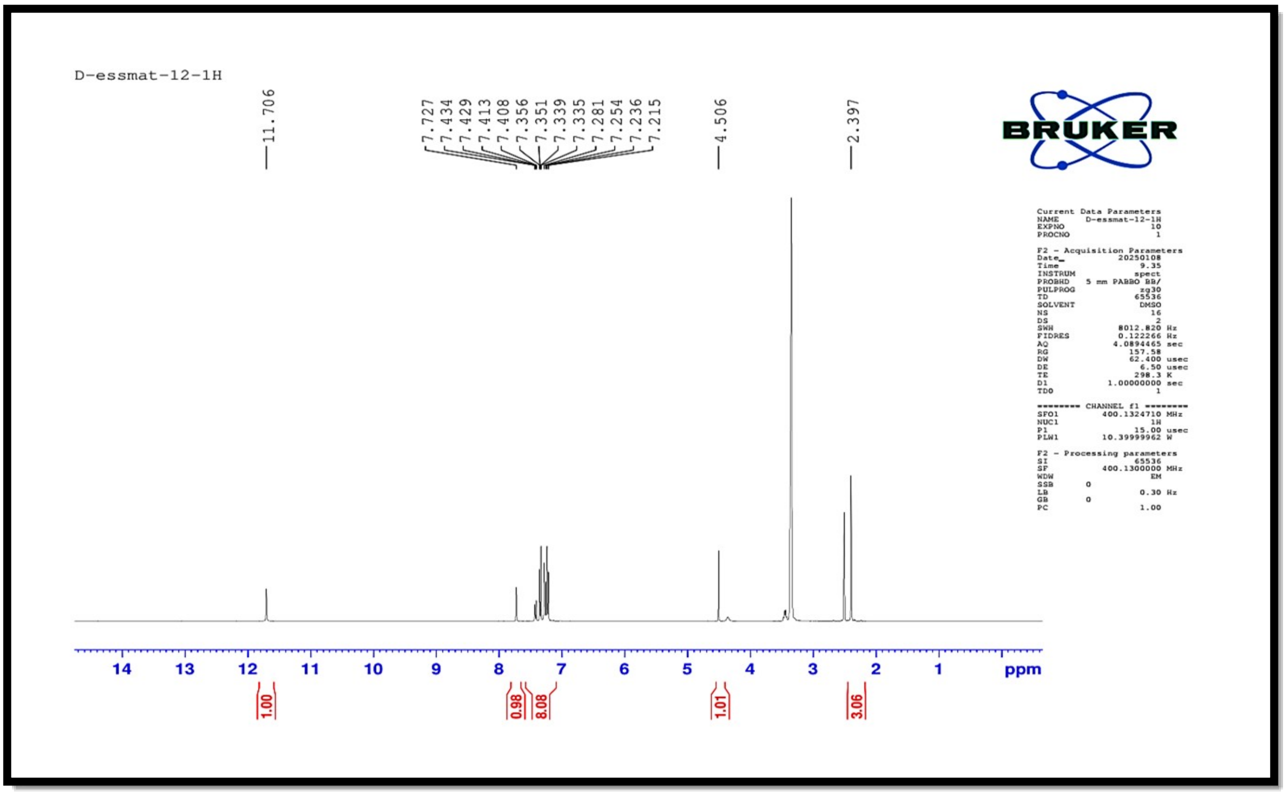

S47. ^{1}H-NMR spectrums for compound **5k**

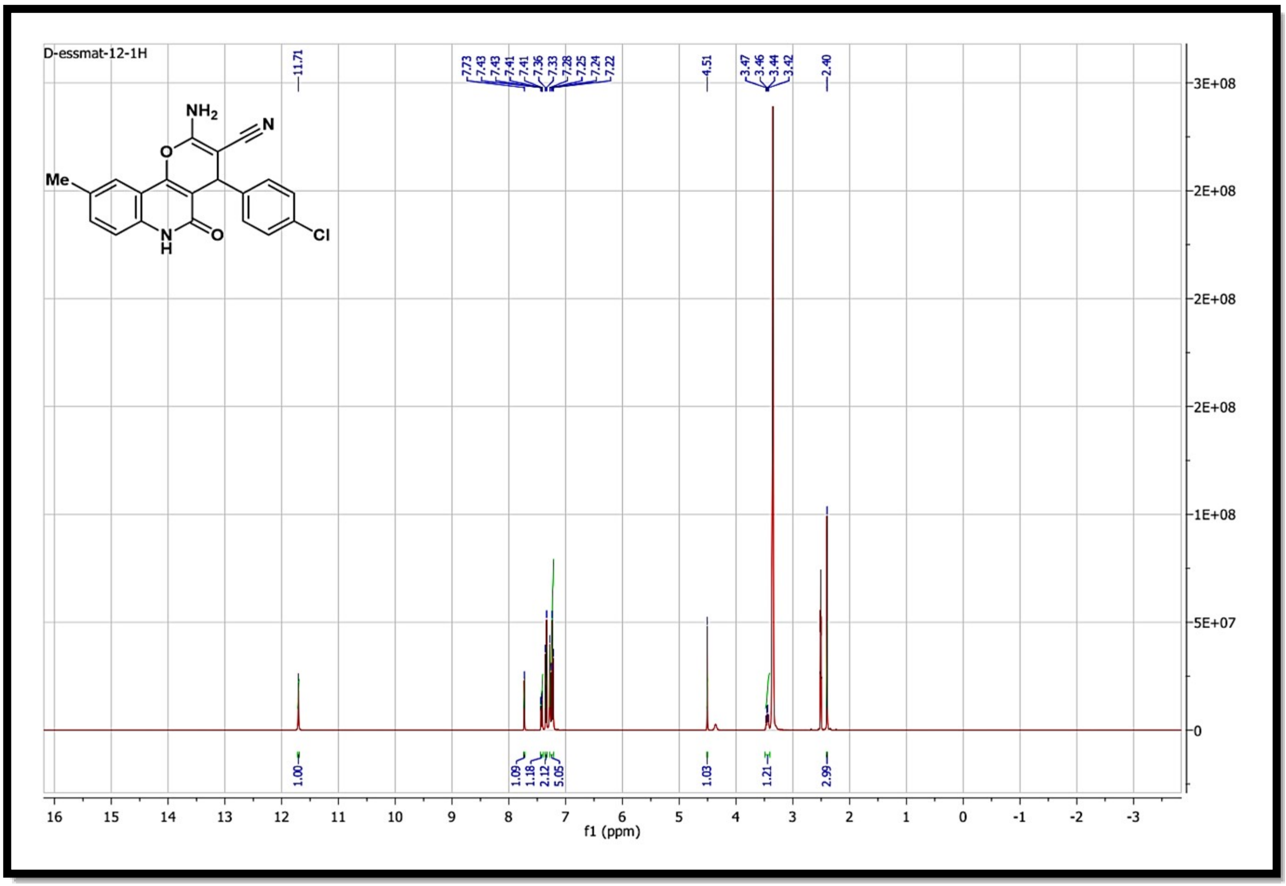

S48. ^{1}H-NMR spectrums for compound **5k**

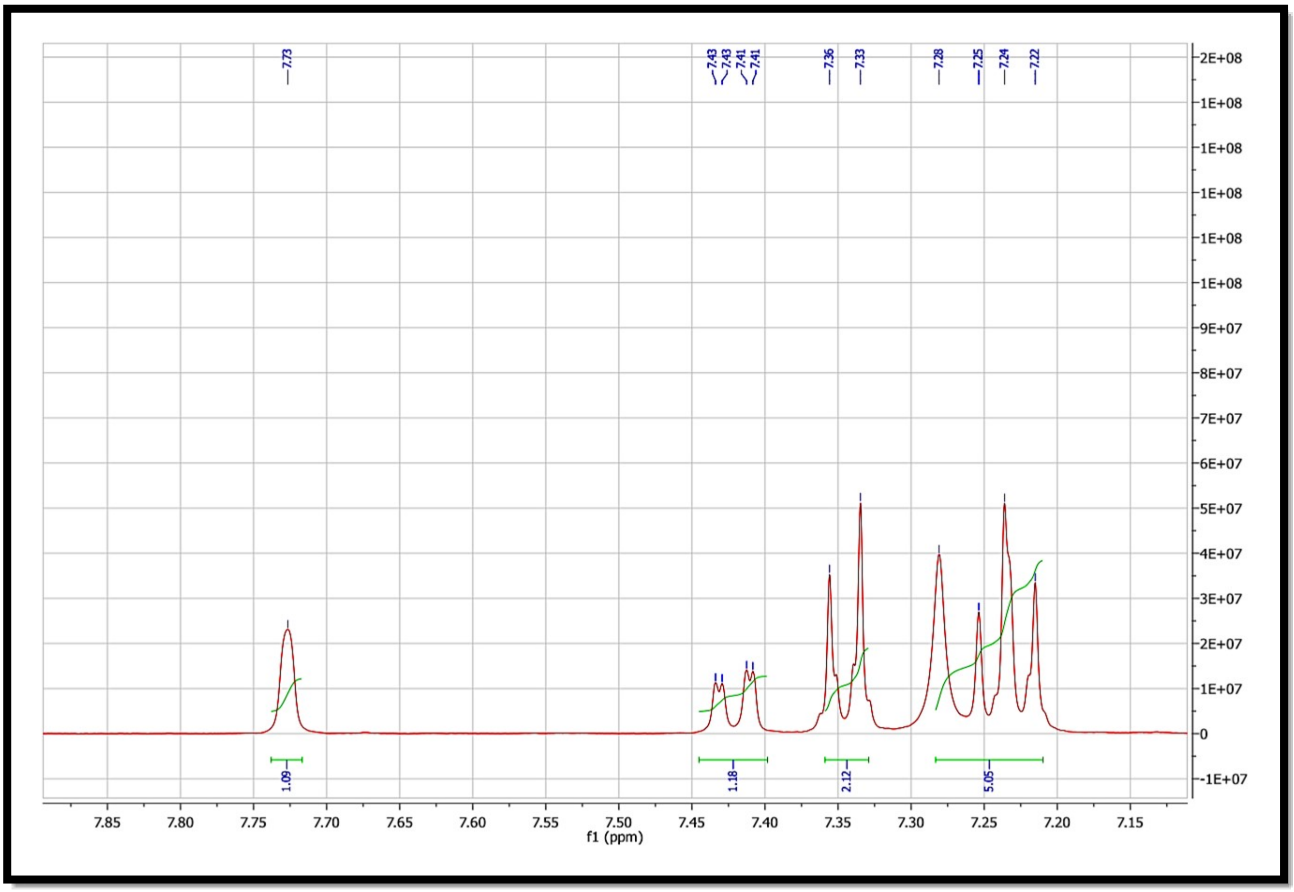

S49. Part of the ^{1}H-NMR spectrums for compound **5k**

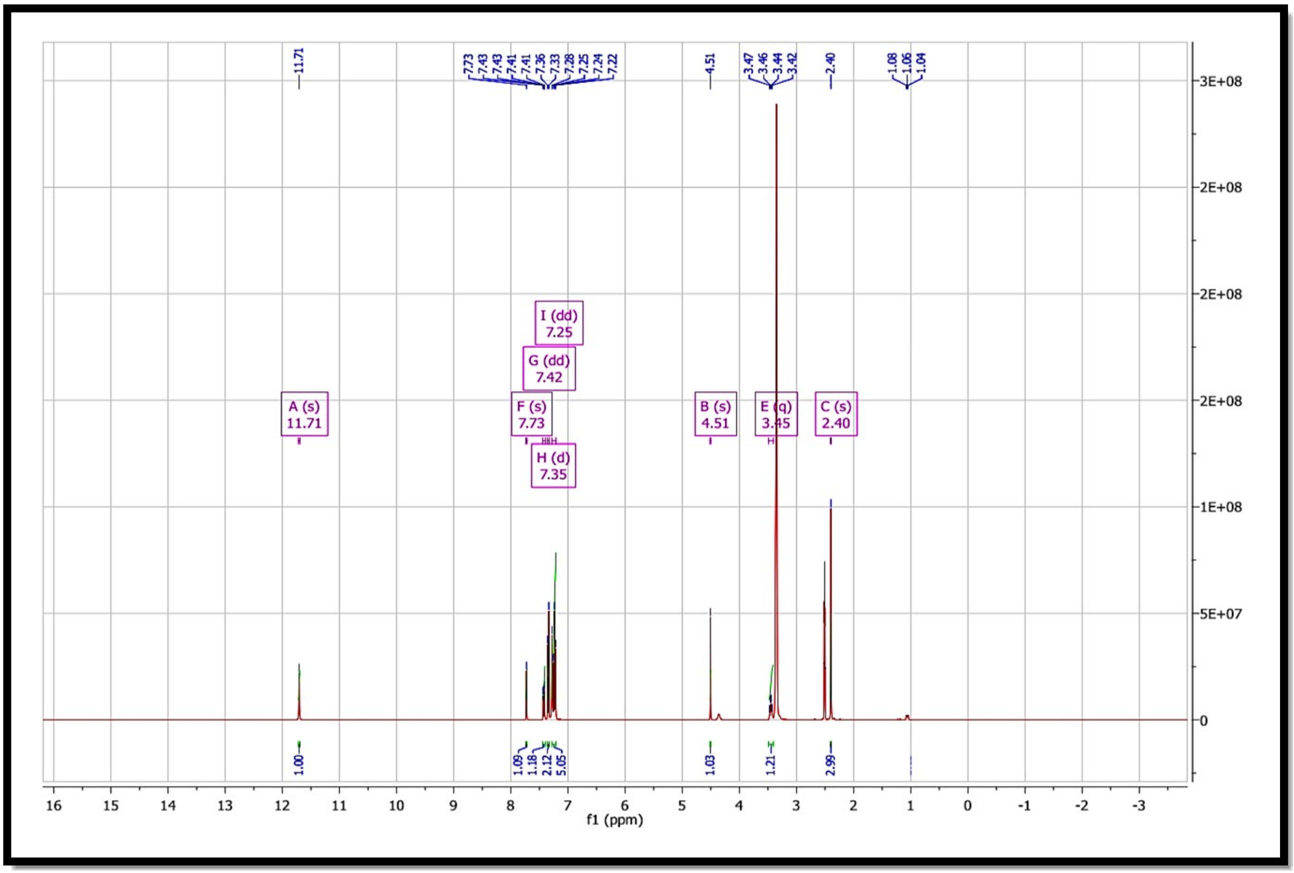

S50. Part of the ^{1}H-NMR spectrums for compound **5k**

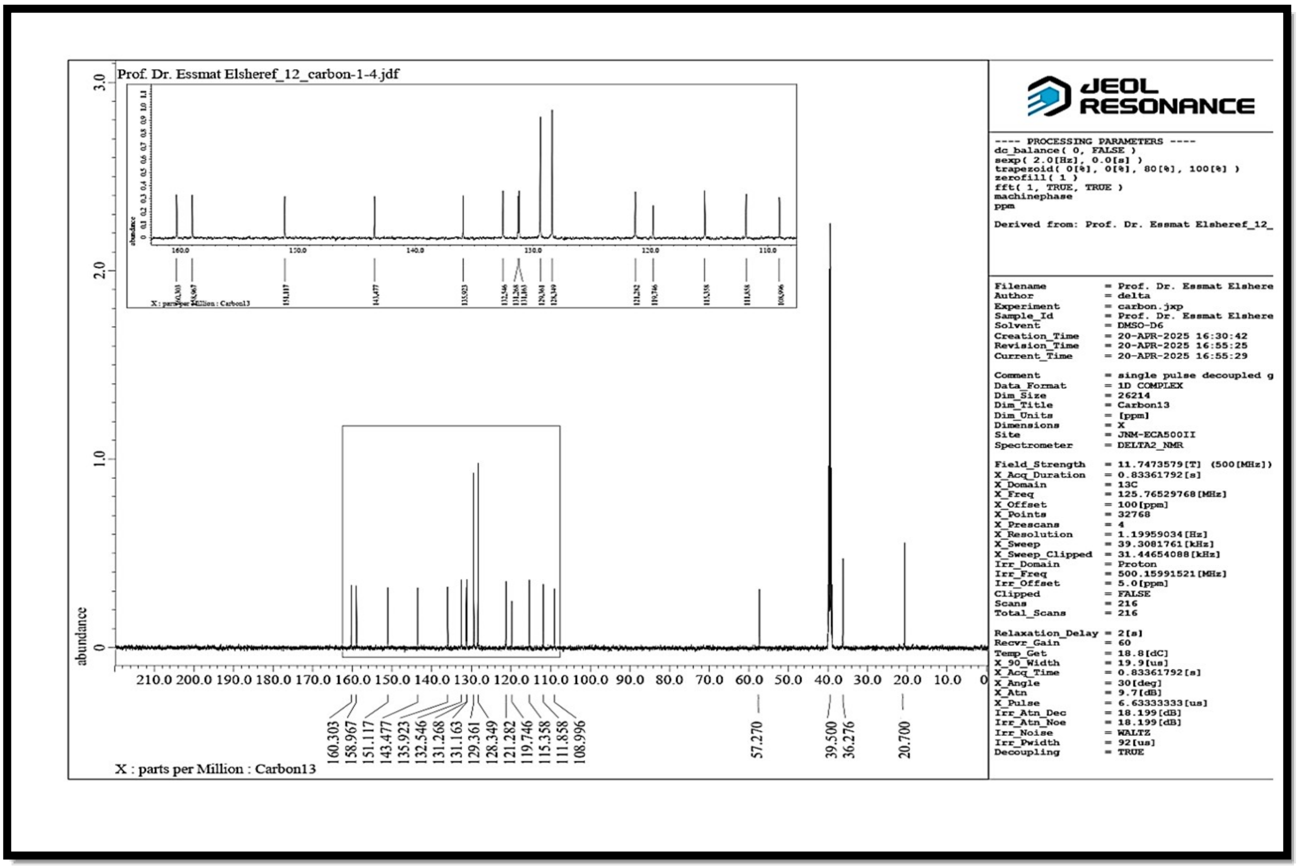

S52. ^{13}C-NMR spectrums for compound **5k**

Spectrums for compound **5l**

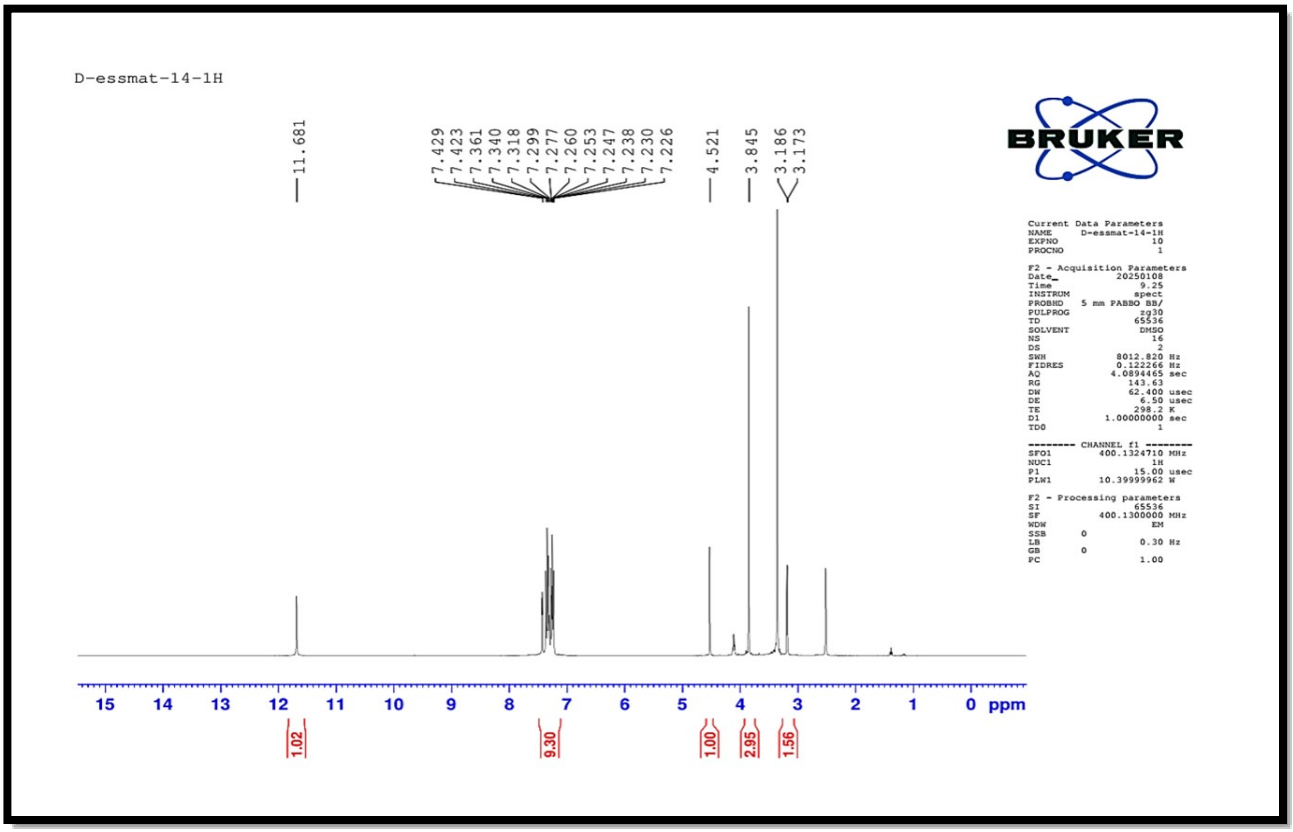

S52. ^{1}H-NMR spectrums for compound **5l**

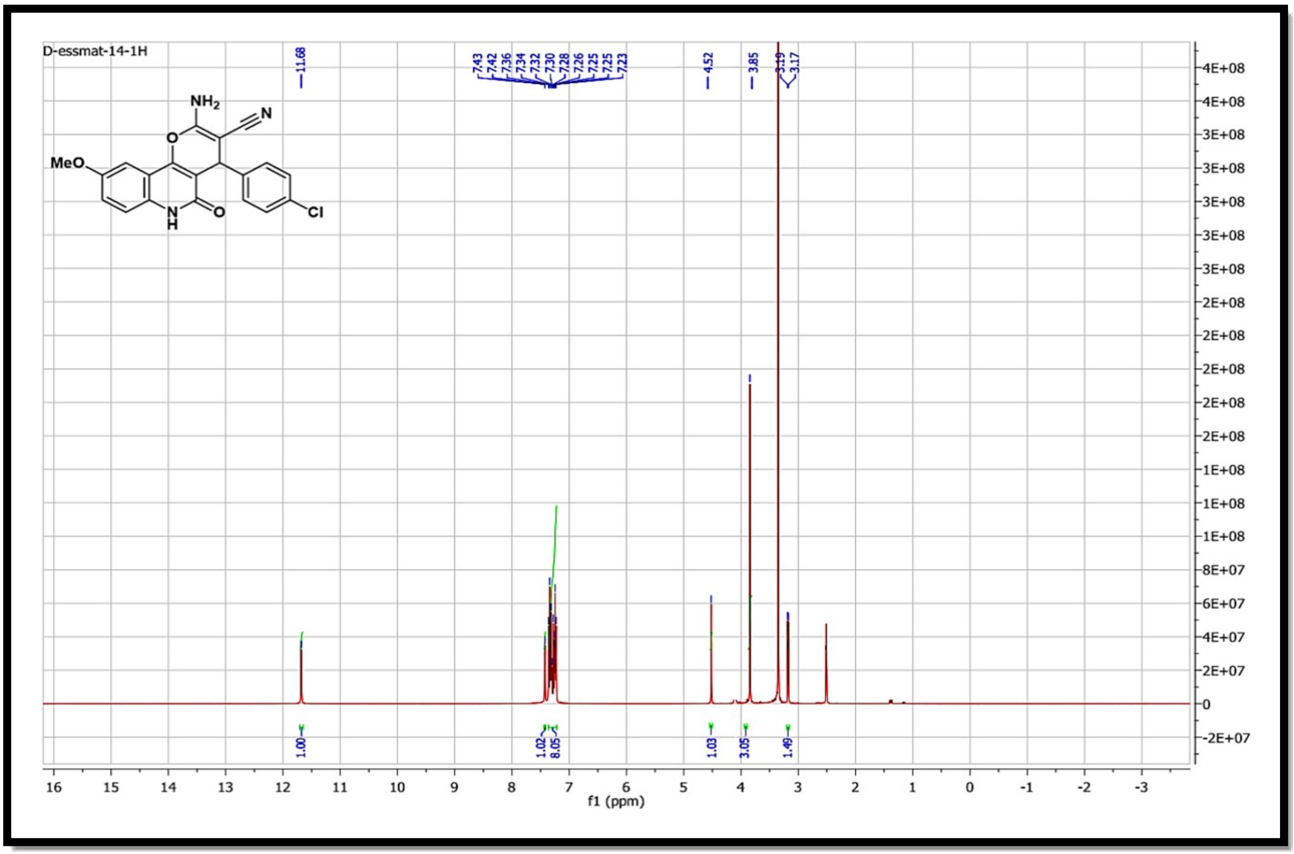

S53. ^{1}H-NMR spectrums for compound **5l**

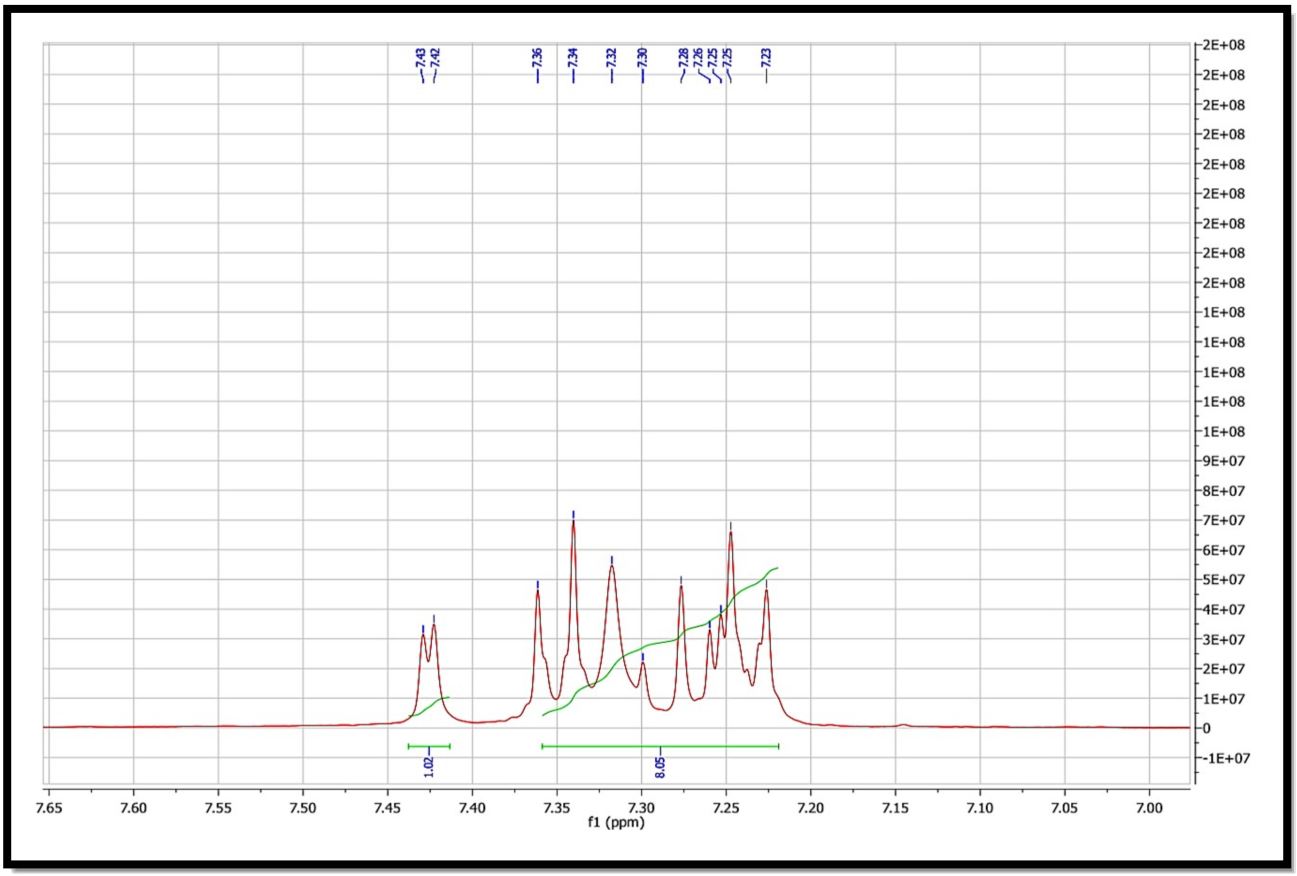

S54. Part of the ^{1}H-NMR spectrums for compound **5l**

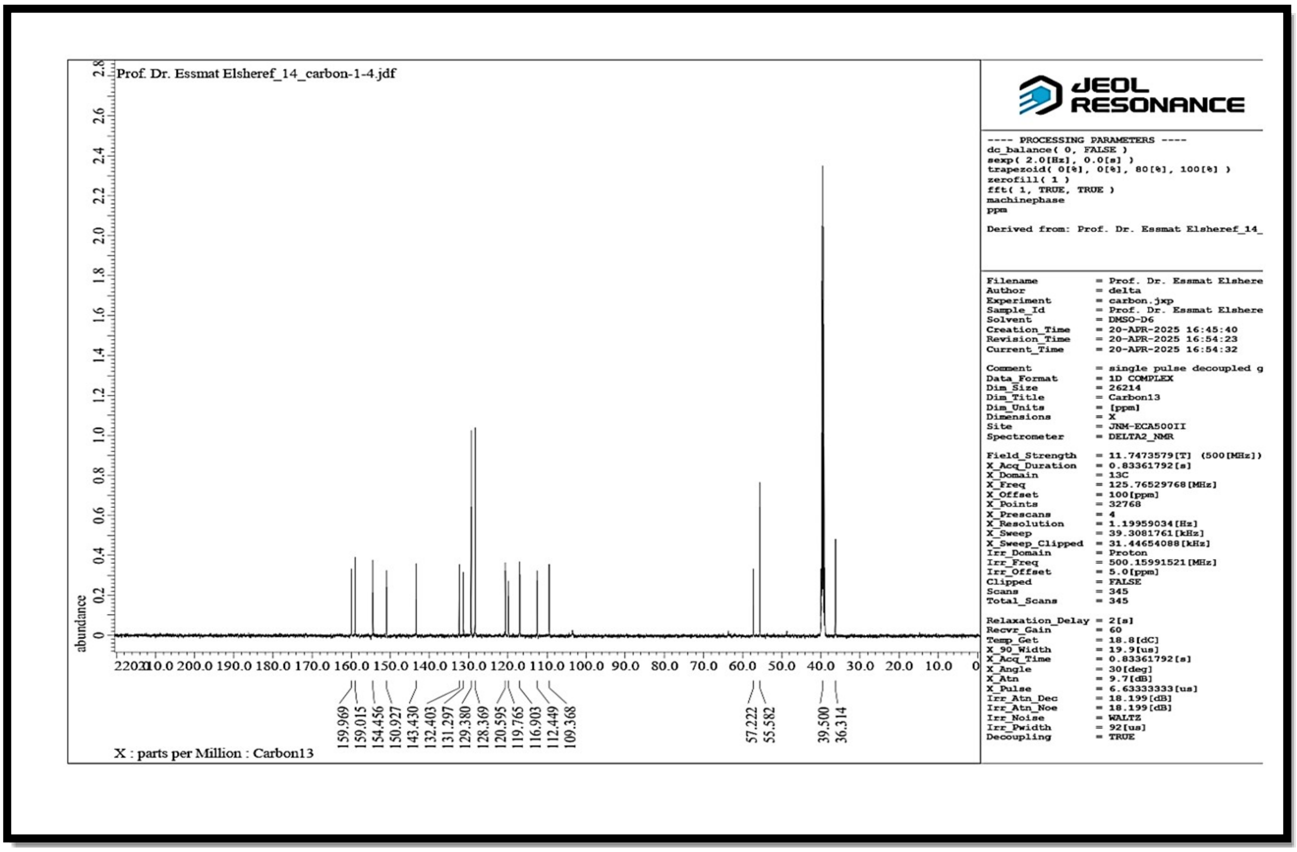

S55. ^{13}C-NMR spectrums for compound **5l**

Appendix A

4. Experimental

4.1. Chemistry

General details

All melting points were determined on Stuart electrothermal melting point apparatus and were uncorrected. All reactions were monitored with thin-layer chromatography (TLC) on Merck alumina-backed TLC plates and visualized under UV light. NMR spectra were measured using a Bruker AV-400 spectrometer), Zagazig University. Chemical shifts are expressed in δ (ppm) versus internal Tetramethylsilane (TMS) = 0 ppm for ^{1}H and ^{13}C. The chemical shifts (δ) are reported in parts per million (ppm) relative to Tetramethylsilane (TMS) as internal standard, and the coupling constants (J) are reported in Hertz (Hz). Splitting patterns are denoted as follows: singlet (s), doublet (d), multiplet (m), triplet (t), quartet (q), doublet of doublets (dd), doublet of triplets (dt), a triplet of doublets (td), and doublet of a quartet (dq). Mass spectra were recorded on a Finnigan Fab 70 eV at Al-Azhar University, Egypt. Elemental analyses were carried out on a Perkin Elmer device at the

Microanalytical Institute of Organic Chemistry, Karlsruhe Institute of Technology, Karlsruhe, Germany.

4.2. Biological evaluation

4.2.1 Cell Viability assay (MTT assay)

MTT assay was performed to investigate the effect of the synthesized compounds on mammary epithelial cells (MCF-10A). The cells were propagated in medium consisting of Ham's F-12 medium/ Dulbecco's modified Eagle's medium (DMEM) (1:1) supplemented with 10% foetal calf serum, 2 mM glutamine, insulin (10 µg/mL), hydrocortisone (500 ng/mL) and epidermal growth factor (20 ng/mL). Trypsin ethylenediamine tetra acetic acid (EDTA) was used to passage the cells after every 2-3 days. 96-well flat-bottomed cell culture plates were used to seed the cells at a density of 104 cells mL^{-1}. The medium was aspirated from all the wells of culture plates after 24 h followed by the addition of synthesized compounds (in 200 µL medium to yield a final concentration of 0.1% (v/v) dimethyl sulfoxide) into individual wells of the plates. Four wells were designated to a single compound. The plates were allowed to incubate at 37°C for 96 h. Afterwards, the medium was aspirated and 3-[4,5-dimethylthiazol-2-yl]-2,5-diphenyltetrazolium bromide (MTT) (0.4 mg/mL) in medium was added to each well and subsequently incubated for 3 h. The medium was aspirated and

150 μL dimethyl sulfoxide (DMSO) was added to each well. The plates were vortexed followed by the measurement of absorbance at 540 nm on a microplate reader. The results were presented as inhibition (%) of proliferation in contrast to controls comprising 0.1% DMSO.

4.2.2. Assay for antiproliferative effect

To explore the antiproliferative potential of compounds propidium iodide fluorescence assay was performed using different cell lines such as Panc-1 (pancreas cancer cell line), MCF-7 (breast cancer cell line), HT-29 (colon cancer cell line) and A-549 (epithelial cancer cell line), respectively. To calculate the total nuclear DNA, a fluorescent dye (propidium iodide, PI) is used which can attach to the DNA, thus offering a quick and precise technique. PI cannot pass through the cell membrane and its signal intensity can be considered as directly proportional to quantity of cellular DNA. Cells whose cell membranes are damaged or have changed permeability are counted as dead ones. The assay was performed by seeding the cells of different cell lines at a density of 3000-7500 cells/well (in 200μl medium) in culture plates followed by incubation for 24h at 37 °C in humidified 5%CO2/95% air atmospheric conditions. The medium was removed; the compounds were added to the plates at 10 μM concentrations (in 0.1% DMSO) in triplicates, followed by incubation for 48h. DMSO (0.1%) was used as control. After incubation, medium was removed followed by the addition of PI (25 μl, 50μg/mL in water/medium) to each well of the plates. At -80 °C, the plates were allowed to freeze for 24 h, followed by thawing at 25 ^{o}C. A fluorometer (Polar-Star BMG Tech) was used to record the readings at excitation and emission wavelengths of 530 and 620 nm for each well. The percentage cytotoxicity of compounds was calculated using the following formula:

$$\% \text{ Cytotoxicity} = \frac{A_C - A_{TC}}{A_C} \times 100$$

Where A*TC*= Absorbance of treated cells and AC= Absorbance of control. Erlotinib was used as positive control in the assay.

4.2.3. EGFR inhibitory assay

Baculoviral expression vectors including pBlueBacHis2B and pFASTBacHTc were used separately to clone 1.6 kb cDNA coding for EGFR cytoplasmic domain (EGFR-CD, amino acids 645–1186). 5′ upstream to the EGFR sequence comprised a sequence that encoded (His)6. Sf-9 cells were infected for 72h for protein expression. The pellets of Sf-9 cells were solubilized in a buffer containing sodium vanadate (100 μM), aprotinin (10 μg/mL), triton (1%), HEPES buffer(50mM), ammonium molybdate (10 μM), benzamidine HCl (16 μg/mL), NaCl (10 mM),leupeptin (10 μg/mL) and pepstatin (10 μg/mL) at 0°C for 20 min at pH 7.4, followed by centrifugation for 20 min. To eliminate the nonspecifically bound material, a Ni-NTA super flow packed column was used to pass through and wash the crude extract supernatant first with 10mM and then with 100 mM imidazole. Histidine-linked proteins were first eluted with 250 and then with 500 mM imidazole subsequent to dialysis against NaCl (50 mM), HEPES (20 mM), glycerol (10%) and 1 μg/mL each of aprotinin, leupeptin and pepstatin for 120 min. The purification was performed either at 4 °C or on ice. To record autophosphorylation level, EGFR kinase assay was carried out on the basis of DELFIA/Time-Resolved Fluorometry. The compounds were first dissolved in DMSO absolute, subsequent to dilution to appropriate concentration using HEPES (25 mM) at pH 7.4. Each compound (10 μL) was incubated with recombinant enzyme (10 μL, 5 ng for EGFR, 1:80 dilution in 100 mM HEPES) for 10 min at 25°C, subsequent to the addition of 5X buffer (10 μL, containing 2 mM $MnCl_2$, 100 μM Na_3VO_4, 20 mM HEPES and 1 mM DTT) and ATP-$MgCl_2$ (20 μL, containing 0.1 mM ATP and 50 mM $MgCl_2$) and incubation for 1h. The negative and positive controls were included in each plate by the incubation of enzyme either with or without ATP-$MgCl_2$. The liquid was removed after incubation and the plates were washed thrice using a wash buffer. The Europium-tagged antiphosphotyrosine antibody (75 μL, 400 ng) was added to each well followed by incubation of 1h and then washing of the plates using buffer. The enhancement solution was added to each well and the signal was recorded at excitation and emission wavelengths of 340 at 615 nm. The autophosphorylation percentage inhibition by compounds was calculated using the following equation:

$$100\% - [(negative\ control)/(positive\ control) - (negative\ control)]$$

Using the curves of percentage inhibition of eight concentrations of each compound, IC50 was calculated. The majority of signals detected by antiphosphotyrosine antibody were from EGFR because the enzyme preparation contained low impurities.

4.2.4. BRAF^{V600E} inhibitory assay

V^{600E} mutant BRAF kinase assay was performed to investigate the activity of tested compounds against BRAF. Mouse full-length GST-tagged BRAF^{V600E} (7.5 ng, Invitrogen, PV3849) was pre-incubated with drug (1 µL) and assay dilution buffer (4 µL) for 60 min at 25°C. In assay dilution buffer, a solution (5 µL) containing $MgCl_2$ (30 mM), ATP (200 µM), recombinant human full length (200 ng) and *N*-terminal His-tagged MEK1 (Invitrogen) was added to start the assay, subsequent to incubation for 25 min at 25°C. The assay was stopped using 5X protein denaturing buffer (LDS) solution (5 µL). To further denature the protein, heat (70° C) was applied for 5 min. 4-12% precast Nu-Page gel plates (Invitrogen) were used to carry out electrophoresis (at 200 V). 10 µL of each reaction was loaded into the precast plates and electrophoresis was allowed to proceed. After completion of electrophoresis, the front part of the precast gel plate (holding hot ATP) was cut and afterwards cast-off. The dried gel was developed using a phosphor screen. A reaction without active enzyme was used as negative control while that containing no inhibitor served as positive control. To study the effect of compounds on cell-based pERK1/2 activity in cancer cells, commercially available ELISA kits (Invitrogen) were used according to manufacturer's instructions.

4.2.5. HER-2 inhibitory assay

The ADP-Glo™ Kinase Assay was used for kinase activity detection, related kinases information: HER-2 (ab60866, Abcam), with a concentration of 100 ng/mL. Target compounds were dissolved in DMSO to obtain drug solutions with different concentrations, and the compound concentrations in the final reaction system (100 µL) were 1 nM, 20 nM, 40 nM, 80 nM, and 100 nM, respectively. 10µL of the solution containing compounds was transferred to a 96-well plate, then added 40 µL of 1 ×

kinase buffer (50 mM HEPES, pH 7.5) to each well, and mixed the mixture in the 96-well plate on a shaker for 10 min. Next, added 25 µL of ADP-Glo™ reagent to the above wells, mixed and incubated for another 40 min. Distributed 10 µL kinase detection reagent to the reaction and incubated for 30 min. Finally, the full-wavelength microplate reader was used to record the OD value.

4.3. Statistical analysis

Computerized Prism 5 program was used to statistically analyzed data using one-way ANOVA test followed by Tukey's as post ANOVA for multiple comparison at P ≤.05. Data were presented as mean ± SEM.
